# Direct conversion of human umbilical cord mesenchymal stem cells into retinal pigment epithelial cells for treatment of retinal degeneration

**DOI:** 10.1038/s41419-022-05199-5

**Published:** 2022-09-12

**Authors:** Xiaoman Zhu, Zhiyang Chen, Li Wang, Qingjian Ou, Zhong Feng, Honglei Xiao, Qi Shen, Yingao Li, Caixia Jin, Jing-Ying Xu, Furong Gao, Juan Wang, Jingfa Zhang, Jieping Zhang, Zhiguo Xu, Guo-Tong Xu, Lixia Lu, Haibin Tian

**Affiliations:** 1 Department of Ophthalmology of Tongji Hospital and Laboratory of Clinical and Visual Sciences of Tongji Eye Institute, Tongji University School of Medicine, Shanghai, 200065 China; 2 Department of Ophthalmology, Shanghai General Hospital (Shanghai First People’s Hospital), Shanghai Jiao Tong University, Shanghai, 200080 China; 3 Department of Physiology and Pharmacology, TUSM, Shanghai, 200092 China; 4 Huzhou college, Zhejiang, 313000 China; 5 The collaborative Innovation Center for Brain Science, Tongji University, Shanghai, 200092 China

**Keywords:** Stem-cell research, Mesenchymal stem cells

## Abstract

Age-related macular degeneration (AMD) is a major vision-threatening disease. Although mesenchymal stem cells (MSCs) exhibit beneficial neural protective effects, their limited differentiation capacity in vivo attenuates their therapeutic function. Therefore, the differentiation of MSCs into retinal pigment epithelial (RPE) cells in vitro and their subsequent transplantation into the subretinal space is expected to improve the outcome of cell therapy. Here, we transdifferentiated human umbilical cord MSCs (hUCMSCs) into induced RPE (iRPE) cells using a cocktail of five transcription factors (TFs): CRX, NR2E1, C-MYC, LHX2, and SIX6. iRPE cells exhibited RPE specific properties, including phagocytic ability, epithelial polarity, and gene expression profile. In addition, high expression of PTPN13 in iRPE cells endows them with an epithelial-to-mesenchymal transition (EMT)-resistant capacity through dephosphorylating syntenin1, and subsequently promoting the internalization and degradation of transforming growth factor-β receptors. After grafting into the subretinal space of the sodium iodate-induced rat AMD model, iRPE cells demonstrated a better therapeutic function than hUCMSCs. These results suggest that hUCMSC-derived iRPE cells may be promising candidates to reverse AMD pathophysiology.

## Introduction

Age-related macular degeneration (AMD) is a leading cause of visual impairment worldwide. Epidemiological investigation found that within the age of 45–85 years, the incidences of early AMD and late AMD were 8.0 and 0.4%, respectively [1]. The pathogenesis of AMD involves a variety of factors, and its etiology is complex. It is characterized by visual function damage caused by apoptosis and loss of function of retinal pigment epithelial cells (RPE) and retinal photoreceptor cells [2]. AMD often leads to severe visual impairment and eventual blindness, which seriously affect the quality of life of patients.

In recent years, stem cell-based treatments have been applied in the clinic and show obvious therapeutic outcomes [3–8]. Mesenchymal stem cells (MSCs) refer to a limited number of cells that can self-renew and differentiate into various cells. Bone marrow-derived MSCs, umbilical cord-derived MSCs (UCMSCs), and adipose-derived MSCs are easily obtained and cultured and have been widely used in research and clinical applications. Previous studies have reported that MSCs have therapeutic effects in AMD [9, 10]. We have also demonstrated that MSC subretinal space transplantation can significantly inhibit photoreceptor cell apoptosis and delay the loss of vision in animal AMD model [11, 12]. Furthermore, we found that different subsets of human UCMSCs (hUCMSCs) have differential therapeutic functions [13]. However, the differentiation ability of MSCs transplanted into the subretinal space in rat AMD models is still controversial [14, 15], and transplantation site also affects the outcomes of MSC-based therapy for retinal degeneration [12, 16]. Therefore, the differentiation of MSCs into RPE cells in vitro and subsequent transplantation into the subretinal space is expected to improve the outcome of cell therapy.

Small molecules and/or RPE-conditioned medium were used to induce the differentiation of MSCs into RPE cells in vitro [17–19]. Whereas, the environment in retina of AMD patients is hostile. Clinical evidence demonstrates that RPE cells undergo epithelial-to-mesenchymal transition (EMT) in AMD [20–22], and the level of transforming growth factor-β (TGF-β), an inducer of EMT, is elevated in the retina of patients with AMD compared with that in normal control eyes [23, 24], suggesting that transplanted RPE cells derived from MSCs are very likely to undergo EMT, subsequently reducing the therapeutic effect of transplanted cells. Therefore, if RPE cells derived from MSCs exhibit anti-EMT ability, the efficacy of MSC-based therapies will be greatly improved.

It has been reported that key transcription factors can transdifferentiate one kind of cells into another lineage of cells [25]. Based on this evidence, we screened a combination of key transcription factors that were able to transdifferentiate hUCMSCs into RPE-like cells, termed induced RPE (iRPE) cells. The iRPE cells had characteristics comparable to those of induced pluripotent stem cell-derived RPE (iPSC-RPE) cells and had anti-EMT properties. Furthermore, the therapeutic function of iRPE cells in sodium iodate (SI)-induced rat AMD model and their anti-EMT mechanisms were investigated.

## Materials and methods

### Animal model

Six-week-old female SD rats (Laboratory Animal Center of Tongji University) were used in this study. All animal procedures were performed according to the institutional guidelines and the Guide for the Care and Use of Laboratory Animals issued by the NIH and the guidelines of the animal experimentation ethics committee of Tongji University (Approved NO. TJAA09620210), and in accordance with the Association for Research in Vision and Ophthalmology Statement for the use of Animals in Ophthalmic and Vision Research. Rats were randomly allocated to experimental groups and no blinding method was used for cell transplantation. There was no animal exclusion criterion. Rats were sacrificed at week 4 or 6 post-transplantation.

### Cell cultures

#### hUCMSC culture

The hUCMSCs were obtained from the Eastern Union Stem Cell and Gene Engineering Co., Ltd. (China). Informed consent was obtained by Eastern Union Stem Cell and Gene Engineering Co., Ltd. Cells were cultured in DMEM/F12 (Gibco, California, USA) containing 10% FBS (Applied StemCell, California, USA) at 37 °C, 5% CO_2_.

#### Induction of adipogenesis, osteogenesis, and chondrogenesis

hUCMSCs were induced to differentiate into adipocytes, osteoblasts and chondrocytes. For adipogenesis, the cells were cultured in adipogenic induction medium (DMEM supplemented with 10% FBS, 10^−7^ M dexamethasone (Sigma, St. Louis, MO), 100 μΜ indomethacin (Sigma), 100 μM 3-isobutyl-1-methyl-xanthine (Sigma) and 10 mg/L insulin (Invitrogen)), and the medium was refreshed every 2 days. Two weeks later, the cells were fixed with 4% paraformaldehyde (PFA) and stained with Oil-Red O (Sigma). For osteogenesis, the cells were maintained in osteogenic induction medium (DMEM supplemented with 10% FBS, 10 mM β-glycerol phosphate (Sigma), 50 μM L-ascorbate-2-phosphate (Sigma) and 5 ng/mL recombinant human bone morphogenic protein-2 (HumanZyme, Chicago, IL)) and the medium was changed with fresh one every 2 days. One week later, the cells were examined for alkaline phosphatase (AKP) activity by vector blue alkaline phosphatase substrate kit III (Vector, Burlingame, CA). For chondrogenesis, the cells were cultured in chondrogenic induction medium includes DMEM supplemented with 10% FBS, 10 ng/mL TGF-β1 (PeproTech, Cranbury, NJ, USA), 0.1 mol/L dexamethasone (Invitrogen), 50 mg/L L-ascorbate-2-phosphate (Sigma) and 50 g/L ITS (Invitrogen). The cells were cultured for two weeks and fixed in 4% PFA, and stained with toluidine blue sodium borate (Sigma).

#### Generation of human iPSCs

Human iPSCs were generated according to a published report [26] with some modifications. Briefly, 5 × 10^5^ hUCMSCs were electroporated with 3 μg of each episomal plasmid, including pCXLE-hSK, pCXLE-hUL, and pCXLE-hOCT3/4-shp53-F (expressing SOX2, KLF4, L-MYC, LIN28, OCT3/4 and p53-targeting shRNA, Addgene, Cambridge, MA, USA) using the following conditions: 900 μV, 500 ms and 1 pulse. After electroporation, cells were reseeded on a Matrigel (BD Bioscience, San Jose, USA) -coated cell culture dish and cultured in DMEM/F12 containing 10% FBS. Two days later, medium was changed into mTeSR^TM^1 medium (STEMCELL Technologies Inc., Vancouver, Canada,) containing 0.25 mM Sodium butyrate (Sigma, St. Louis, MO, USA). The iPSC colonies were picked out by cloning ring and subcultured in mTeSR^TM^1 medium.

#### Differentiation of iPSCs into RPE cells

Differentiation was conducted according to the previous report with modification. Briefly, once growing to confluency, iPSCs were cultured in differentiation medium containing DMEM/F12, 15% knockout serum replacement, 1% nonessential amino acids, 2 mM glutamine, 50 U/ml penicillin, 50 mg/ml streptomycin (all from Invitrogen, Carlsbad, CA, USA), and 10 mM nicotinamide (Sigma) in 6-well culture dishes (Costar, Corning Inc., Corning, NY) pretreated with Poly (2-hydroxyethyl methacrylate) (Sigma). 20 ng/ml Activin A (PeproTech Inc, Rocky Hill, NJ) was supplemented during the third and fourth weeks. After 8 weeks in suspension, pigmented areas were isolated by a surgical blade no. 15 and 30–50 clusters were cultured in dishes precoated with Matrigel and cultured 6 weeks in differentiation medium. For subculture, iPSC-RPE cells were dissociated with 0.25% trypsin/0.53 mM EDTA, and cultured in differentiation medium in dishes precoated with Matrigel.

#### ARPE19 cells and HELA cells

ARPE19 cells (ATCC, Research Triangle Park, NC, USA) were cultured in DMEM/F12 containing 10% FBS, and HELA cells (ATCC) were cultured in RPMI-1640 (Invitrogen) plus 10% FBS.

### Viral infection

For generating retroviruses, human cDNAs of *six6*, *nr2e1*, *lhx2*, *crx, and mitf-a* were obtained by PCR amplification from iPSCs differentiated cultures-derived mRNA templates (obtained from iPSCs differentiating into RPE cultures from second to fourth weeks) and cloned into pMXs vectors (Addgene). pMXs-sox2, pMXs-c-myc, pMXs-klf4, and pMXs-gfp were bought from Addgene. The packaging plasmids are pCMV-VSVG and gag/pol (Addgene). HEK293FT cells were seeded at a density between 5.0–7.0 × 10^4^ cells/cm^2^ and transfected by Lipofectamine 2000 (Invitrogen) with each transcription-factor retrovirus. 8 h later, media were refreshed. Individual supernatants containing virus were harvested at 48 and 72 h post-transfection and filtered with 0.45 μm PVDF membrane (Millipore, Boston, USA). hUCMSCs were plated in 10 cm culture dishes at 100,000 cells per well. The next day, cells were infected with an equal ratio of a combination of retroviruses. After transfection for 7 days, clones constituted by cells with polygonal morphology were observed in the cultures. Clones were picked out by cloning ring (Sigma) and subcultured with DMEM/F12 supplemented with 10% FBS. The transdifferentiation efficiency was evaluated by number of clones per 100,000 cells after giemsa staining (Sigma). For syntenin1-Flag-iRPE cells preparation, *flag* was cloned into corresponding vectors to produce pMXs-syntenin1-flag virus and used to transfect iRPE cells.

### Quantitative real-time polymerase chain reaction (qRT-PCR)

Total RNA was extracted and reverse transcription was performed using Primescript™ RT Master Mix kit (Takara, Shiga, Japan). qRT-PCR was performed in a Chromo4 instrument cycler (Bio-Rad, Hercules, USA) using Superreal Premix plus kit (Tiangen Biotech, Beijing, China). PCR amplification was carried out with the following cycling parameters: denaturation at 95 °C for 5 min, followed by 40 cycles of 95 °C for 30 s, 60 °C for 30 s. Primer sequences (Synthesized by Sangon Biotech Co., Ltd., Shanghai, China) were listed in Tables S1, 2.

### Immunostaining

For immunofluorescence analysis, fixed cells, cryosections from eyes and matrigel containing transplanted cells in nude mice, were permeabilized with 0.1% Triton X-100 (Sigma) for 2 min, washed with PBS, and then blocked with 2% bovine serum albumin (BSA, Sigma) in PBS. The sections were incubated with the primary antibodies (1:200) against ZO-1, FN1, CRALBP, and BEST1 (Proteintech, Rosemont, IL, USA); against TYRP1, α-SMA, pSMAD2, pSMAD3, Rhodopsin, PEDF, and Ki67 (Abcam, Cambridge, UK); against pp38 (CST, Danvers, MA, USA) overnight at 4 °C. They were then washed three times with PBS, followed by incubation with the fluorescent secondary antibodies (1:1000, Invitrogen) overnight. 4,6 diamidino-2-phenylindole dihydrochloride (DAPI, Sigma) was used to indicate the nucleus. The samples were then examined by fluorescence microscope (Olympus IX73, Tokyo, Japan). Antibodies were listed in Table S3.

### Western blotting

The cells were lysed by RIPA buffer containing protease and phosphatase inhibitor (Sigma). The protein extracts (10 μg per sample) were separated by 10% SDS-PAGE gels, and transferred onto polyvinylidene difluoride membranes (Millipore, Bedford, MA). After blocked with 3% BSA in PBS for 1 h, membranes were incubated with primary antibodies against RPE65 (1:1000, Novus Biologicals, Centennial, CO, USA); against TYRP1 (1:1000), MERTK (1:1000), FN1 (1:1000), α-SMA (1:2000), pSMAD2 (1:500), SMAD2 (1:1000), pSMAD3 (1:1000), SMAD3 (1:1000) TGF-β receptor1 (TGF-βR1) (1:1000), Abcam; against pERK1/2 (1:1000), ERK1/2 (1:1000), CST; against Claudin19 (1:1000, Invitrogen); against Flag (1:2000, MBL International, Woburn, MD, USA); against CRALBP (1:1000), syntenin1 (1:1000), DUSP4 (1:1000), DUSP10 (1:1000), PHLPP1 (1:1000), PPM1L (1:1000), PPP1CC (1:1000), PPP2R5A (1:1000), PTPN13 (1:1000), TGF-βR2 (1:1000), and β-Actin (1:5000), Proteintech; for 12 h at 4 °C, followed by incubation with corresponding secondary antibodies for 1 h at room temperature. The blots were visualized with a chemiluminescence imaging system (Tanon 5200, Shanghai, China) and quantified with ImageJ software (Version 1.48 v). Antibodies were listed in Table S3.

### Transmission electron microscopy (TEM)

Cells were seeded into the upper chamber of a 24-transwell plate (0.4 μm pore, BD Biosciences, San Diego, CA, USA). Three weeks later, cells were fixed in 2.5% glutaraldehyde-buffered solution at room temperature for 2 h and rinsed with PBS. Cells were then treated with 1% ice-cold osmium tetroxide for 1 h. After osmication, tissues were rinsed in PBS and processed through a battery of ethanol dehydration steps (50, 70, 85, and 100% ethanol) for plastic embedding. The micrographs were obtained by a transmission electron microscopy (Hitachi, HT7800, Japan).

### Photoreceptor outer segment (POS) phagocytosis assay

Porcine eyes were obtained from abattoir and POSs were isolated and purified from porcine retinas as described previously [27]. Purified POSs were labeled with NHS-LC-Biotin (Thermo Fisher Scientific, Carlsbad, CA, USA) and resuspended in culture medium at a concentration of 5 × 10^7^ POSs/mL. Cells were incubated with POSs at 37 °C, 5% CO_2_ in a humidified incubator for 4 h. Non-phagocyted or unbound POSs were removed by washing three times with PBS. Cells were fixed by 4% paraformaldehyde (PFA, Sigma) in PBS and incubated with cy3-labeled avidin (Invitrogen) for 10 min. ZO-1 immunostaining was used to show the boundary of cells. DAPI was used to label nuclei. The samples were then examined by fluorescence microscope (Olympus IX73). Z-stack images were obtained using a Nikon confocal microscope (Nikon A1R, Nikon Instruments Inc., Tokyo, Japan).

### Transepithelial electrical resistance (TER) analysis

3 × 10^4^ cells were seeded into the upper chamber of a 24-transwell plate (0.4 μm). After 21 days, TER was analyzed. Briefly, the 24-transwell plate was removed from the incubator and placed at room temperature for 30 min. TER values were measured by volt-ohm meter using an electrode (EVOM2; World precision Instruments, Sarasota, FL, USA).

### Permeability analysis

When the cells grew for 21 days in the upper chamber of a 24-transwell plate (0.4μm), 200 μL culture medium containing 10 μg/mL horseradish peroxidase (HRP, Sigma) was added into the upper chamber. 30 min later, 20 μL medium was taken from the lower chamber, mixed with 150 μL TMB chromogenic reaction solution (Sigma). The absorbance value (OD value) at the 450 nm wavelength was measured.

### β- Galactosidase (Gal) staining

iPSC-RPE cells (passage 12), iRPE cells (passage 35) and ARPE19 (passage 35) were cultured in 12-well plates. Cells were washed once with PBS and fixed in fixative solution for 15 min at room temperature. Cells were then washed three times with PBS and incubated with 1 mL X-gal containing solution (C0602, Beyotime, Shanghai, China) overnight at 37 °C. The plate was sealed with Parafilm to prevent evaporation of the staining medium. After incubation, cells were washed with PBS and observed under microscope. DAPI staining was used to calculate the number of cells.

### RNA-seq analysis

RNA library pools of the cells were established following the protocols of the Illumina mRNA-seq with 50 ng of RNA from hUCMSCs, iPSC-RPE cells, iRPE cells, and ARPE19 cells, and experiments were performed in the MAJORBIO company (Shanghai, China). Filtering and quality control checks of the raw reads from RNA-seq had been done by FastQC. The clean reads were mapped to reference sequences using SOAP2 aligner. The gene expression levels were calculated using TPM method. Log_2_ fold change (FC) of TPM (iRPE cells)/TPM (hUCMSCs) was used to identify differentially expressed genes (DEGs) between iRPE cells and hUCMSCs. Only those genes indicating |log_2_FC| > 2 and adjusted *p* < 0.05 were regarded as DEGs.

### Enzyme-linked immunosorbent assay (ELISA)

Quiescent hUCMSCs, iPSC-RPE cells, iRPE cells, and ARPE19 cells were cultured in 24-well culture plate or 24-transwell plate for 21 days. The supernatants from 24-well culture plate or from upper and lower chambers of 24-transwell plate were collected. PEDF and VEGF were quantified by PEDF ELISA kit (Elabscience, Wuhan, China) and VEGF ELISA kit (Proteintech).

### Generation of lentiviruses to knockdown target genes

Lentiviral pLVX-shRNA2-ZsGreen1 (Takara) vector were used to prepare lentiviruses. The packaging plasmids are psPAX2 and pMD2.G. The targeting sequences of the two shRNA for each phosphatases and syntenin1 were included in Table S4. HEK293FT cells were transfected with vectors. Individual supernatants containing virus were harvested at 48 h and used to infect iRPE cells and shPtpn13-iRPE cells. The positively transfected cells were sorted by FACS based on ZsGreen expression. The reduced expression of target genes at transcript level was determined by qRT-PCR. We chose the most efficient shRNA out of the two to do next experiments.

### Flow cytometry

iRPE cells transfected with shRNA or GFP were detached and dissociated into single cells with 0.25% trypsin/0.53 mM EDTA buffer, then resuspended in PBS containing 1% BSA. ZsGreen1+ or GFP+ cells were sorted and collected on a FACS Aria II instrument using Cellquest software (BD Bioscience). For TGF-β receptor (TGF-βR) detection, cells were incubated in PBS containing primary antibody against TGF-βR1 or TGF-βR2, and then with PE-labeled secondary antibody; For hUCMSC identification, cells were incubated with PBS containing antibodies against CD105, CD90, CD73, CD44, CD29, CD34, CD45, and MHCII.

### TGF-β stimulation

Cells (5 × 10^4^/cm^2^) were seeded in culture plate treated with 10 ng/mL TGF-β1 or 10 ng/mL TGF-β2 (PeproTech). Cells were cultured for 8 days and fixed with 4% PFA and the corresponding RPE markers and EMT markers were analyzed by immunostaining and Western blotting. For phosphorylation analysis, cells (5 × 10^4^/cm^2^) were seeded in 24 or 6-well culture plate and cultured for 1 day. Cells were stimulated with 10 ng/mL TGF-β1 or 10 ng/mL TGF-β2 and fixed in 24-well culture plate for immunostaining after 1 h treatment, or lysed in 6-well culture plate for Western blotting after 1, 2, 4, or 8 h treatment.

### Co-immunoprecipitation (Co-IP)

Protein A + G magnetic beads (20 μL) were incubated with 5 μg antibodies (against PTPN13, FLAG, or Phospho-Tyrosine) or normal 5 μg IgG for 1 h. 5 × 10^6^ cells were lysed by 200 μL IP lysis buffer (Beyotime) and incubated with prepared conjugated Protein A + G magnetic beads at 4 °C overnight. The beads were washed with TBS for three times and the binding proteins were eluted with protein loading buffer at 95 °C for 5 min. After centrifugation, the samples were collected and used for Western blotting.

### Mass spectrometry analysis

Co-IP samples were digested by trypsin and condensed, then dissolved in Nano-HPLC Buffer, protein components were separated by Nano-HPLC (UltiMate 3000 RSLCnano, ThermoFisher Scientific). Co-IP proteins were identified by Mass spectrometer (Q Exactive plus, ThermoFisher Scientific). The mass range for MS scans was 350–2000 m/z. The mass spectrometer was conducted in standard MS/MS data-dependent acquisition mode. The MS/MS raw files were searched in human protein RefSeq database (uniprot-Human) with software ProteomeDiscover 2.1 (ThermoFisher Scientific).

### Cell transplantation

The six-week-old SD rats were intravenously injected with sodium iodate (SI, 50 mg/kg, Sigma) to induce AMD model, 6 h later, rats were received hUCMSCs, iRPE cells, or iPSC-RPE cell transplantation as previously reported with modifications [13]. SI was metabolized within dozens of minutes in vivo [28, 29], thus the transplanted cells will not be damaged by SI at this time point. Briefly, rats were anesthetized by 2% sodium pentobarbital. A channel was created by inserting a 30-gauge needle, behind the limbus, into vitreous chamber. A 33-gauge needle was inserted into the subretinal space of the central retina and 3 microliters of the cell suspensions (1 × 10^5^ cells/μL) were injected. The eyes received a sham subretinal injection of PBS were used as control. All the rats received cyclosporin A for immunosuppression (210 mg/L in drinking water, Xinfu Pharmaceutical Co., Ltd., Jiangsu, China) two days before the transplantation until the end of the experiment.

### Electroretinogram (ERG) examination

Following cell transplantation, ERG examination was performed at weeks 1, 2, 4, and 6 after cell transplantation with AVES-2000 electrophysiological apparatus (Kanghuaruiming S&T, Chongqing, China) as described previously [13]. An intensity of 6.325e-2cd•s/m enabled the recording of photoreceptor response.

### Retina structure assessment

Rats were sacrificed with an overdose of sodium pentobarbital at post-transplantation 4 and 6 weeks. The eyeballs were removed immediately and fixed in 4% PFA. The embedded tissues were sectioned (10μm thickness) along the vertical meridian of the eye. The samples collected at week 4 post-transplantation were used to analyze the EMT of iRPE cells, and the samples collected at week 6 post-transplantation were used to assess the retinal degeneration. Nuclei in the sectioned tissue were counterstained with DAPI. The degree of retinal degeneration was assessed by the thicknesses (μm) of retinal outer nuclear layer (ONL) which were measured at 10 different points within both the nasal and temporal hemispheres.

### Terminal deoxynucleotidyl transferase-mediated deoxyuridine triphosphate nick and labeling (TUNEL) assay

Eye samples were collected at week 4 post-transplantation. TUNEL assay was conducted with In Situ Cell Death Detection Kit (Roche, Diagnostics, Switzerland), according to the manufacturer’s instructions. Nuclei were counterstained with DAPI. The apoptosis of ONL was assessed based on the counts of TUNEL+ cells per field.

### Tumorigenesis

3 × 10^6^ cells were embedded in 50 μL of Matrigel and subcutaneously injected into back of 6-week-old female nude mice using a 1 mL syringe with a 28G needle. Animals were monitored for 2 weeks (HELA cell group) and 4 months (iPRE cell group and hUCMSC group). At the end of the experiments, mice were sacrificed and cell masses were picked out and fixed with 4% PFA for cryosection preparation. HELA cells were used as positive control.

### Statistical analysis

All data were expressed as means ± standard deviation (SD). Differences between two groups were assessed with the two-tailed Student’s unpaired t test. The one-way ANOVA and post hoc Bonferroni’s test was used to assess differences between more than two groups. No statistical methods were used to predetermine the sample size. Mice were randomly allocated to experimental groups. No blinding method was used for assessing outcome. There was no animal exclusion criterion. The variance was similar between the groups that were being statistically compared. All experiments were repeated twice. Data were analyzed using GraphPad Prism 9 software (GraphPad Software, San Diego, CA, USA). Statistical significance was set at *P* < 0.05.

## Results

### The combination of 5TFs (CRX, NR2E1, C-MYC, LHX2, and SIX6) transdifferentiates hUCMSCs into iRPE cells

**Fig. 1:**
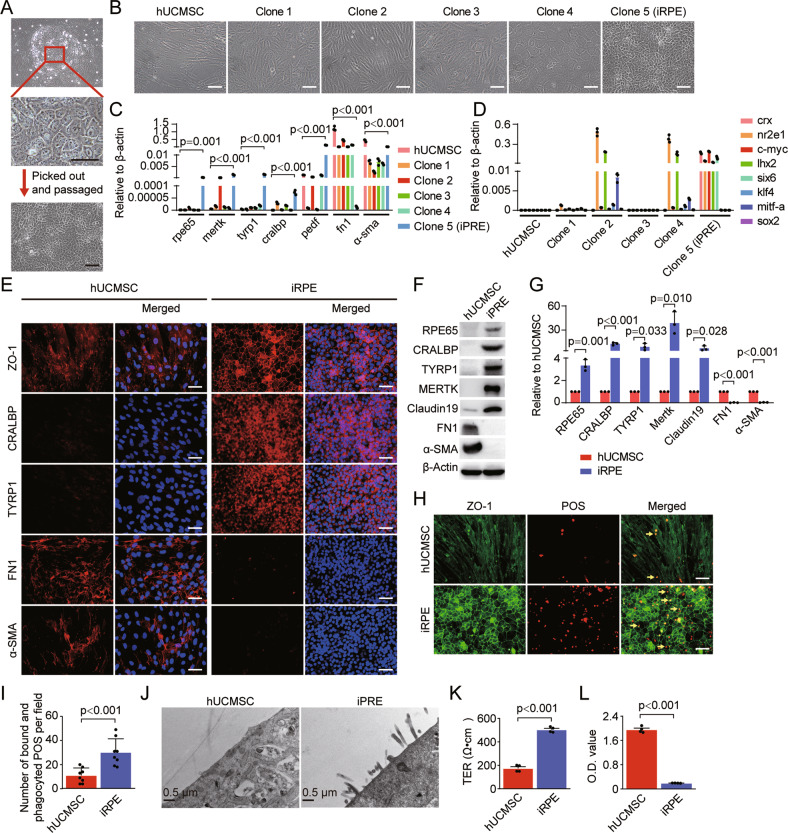
Five TFs transdifferentiate hUCMSCs into iRPE Cells. Retroviruses carrying 8 TF genes *(crx, nr2e1, c-myc, lhx2, six6, klf4, mitf-a, and sox2*) were used to transfect hUCMSCs. **A** The representative colony of hUCMSCs transfected with 8 TFs was picked out and passaged. **B** Representative images of induced cell colonies. **C** The expression levels of RPE-specific markers, EMT-related markers, and **D** exogenous TFs were determined by qRT-PCR (*n* = 3, *P* value measured by one-way ANOVA and post hoc Bonferroni’s test). RPE-specific and EMT-associated markers were detected by **E** immunostaining, **F** Western blotting, and **G** quantitative analysis (*n* = 3, *P* value measured by Student’s unpaired t test). **H** Total bound and phagocyted POSs (pointed by arrows) in hUCMSCs and iRPE cells. **I** Quantification of phagocytosis, as determined by the number of bound and phagocyted POS per field (*n* = 8, *P* value measured by Student’s unpaired t test). **J** Electron micrographs of hUCMSCs and iRPE cells. **K** TER analysis (*n* = 4, *P* value measured by Student’s unpaired t test). **L** HRP permeability assay (*n* = 4, *P* value measured by Student’s unpaired t test). Scale bar = 50 μm. Results are expressed as mean ± SD.

hUCMSCs were identified by exhibiting fibroblast-like morphology and the capacity to differentiate into osteocytes, adipocytes, and chondrocytes in vitro (Fig. S1A), as well as expressing the surface markers CD105, CD90, CD73, CD44, and CD29, but not CD34, CD45, and MHCII (Fig. S1B). We used a mixture of eight viruses burdened with *crx*, *nr2e1*, *c-myc*, *lhx2*, *six6*, *klf4*, *mitf-a*, and *sox2* to infect hUCMSCs. Seven days after virus infection, different morphological clones appeared in the cultures, and clones were picked for amplification using a clone ring (Fig. 1A). The cells of clone 5 showed polygonal morphology, typical RPE cell characteristics (Fig. 1B), along with high expression of RPE markers *rpe65*, *mertk*, *tyrp1*, *cralbp*, and *pedf*, and low expression of EMT markers *fn1* and *α-sma*, detected by qRT-PCR (Fig. 1C), and termed iRPE cells. The transdifferentiation efficiency of iRPE cells was 47 ± 8.67 clones per 100,000 hUCMSCs in 10-cm dish (Fig. S2A–C). The eight exogenous transcription factors in iRPE cells were further analyzed, and only exogenous transcription factors *crx*, *nr2e1*, *c-myc*, *lhx2*, and *six6* were expressed in iRPE cells (Fig. 1D). We next confirmed that the combination of these five TFs was essential and sufficient to transdifferentiate hUCMSCs into iRPE cells (Fig. S2D, E). Immunofluorescence staining showed that compared with hUCMSCs, iRPE cells had continuous tight junction ZO-1 staining, high expression of RPE markers, and low expression of the EMT markers FN1 and α-SMA (Fig. 1E). Western blotting further verified these immunofluorescence results (Fig. 1F, G). An important function of RPE cells is to engulf and eliminate exfoliated POS and maintain the normal renewal of visual cells. The apical membrane of RPE cells harbors microvilli which participate in the phagocytic function of the RPE cells, and MERTK (The TAM receptor tyrosine kinase) is expressed by RPE cells and mediates the recognition and endocytosis of the POS [30]. Compared with hUCMSCs, iRPE cells phagocytized more porcine POS (Fig. 1H, I and Fig. S3). Furthermore, TEM showed that iRPE cells had more microvilli on the cell membrane than hUCMSCs (Fig. 1J), and western blotting showed that iRPE cells expressed higher level of the MERTK than hUCMSCs (Fig. 1F, G). RPE cells normally form a highly polarized monolayer between neuroretina and the choroid, this epithelial barrier regulates the transport of macromolecules and ions, and TER measurement and permeability assay are always used to evaluate the integrity of the epithelial barrier [31]. The TER value of iRPE cells was significantly higher than that of hUCMSCs (Fig. 1K). We further examined the cellular permeability of iRPE, and the results showed that the amount of HRP passing through the iRPE monolayer was significantly lower than that of hUCMSCs (Fig. 1L). These results indicate that iRPE cells have characteristics of RPE cells.

### iRPE cells exhibit comparable characteristics with iPSC-RPE cells

**Fig. 2:**
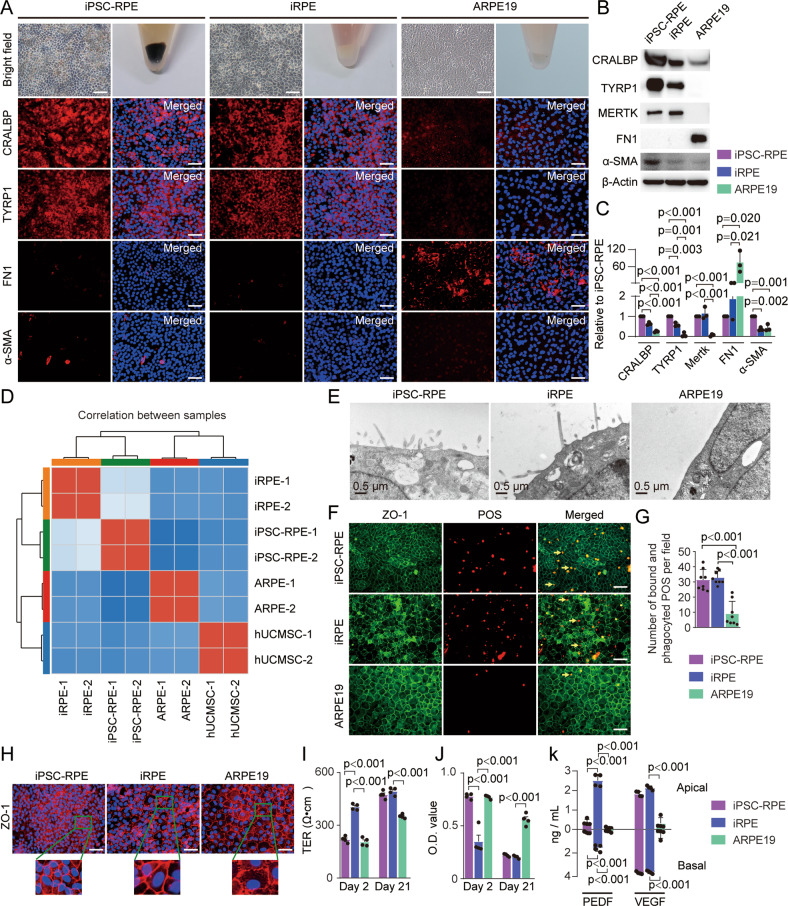
iRPE cells exhibit comparable characteristics with iPSC-RPE cells. Pigment generation, RPE-specific and EMT-associated markers in iPSC-RPE cells, iRPE cells, and ARPE19 cells were detected by **A** pigment generation and immunostaining, **B** Western blotting and **C** quantitative analysis (*n* = 3). **D** The Pearson correlation coefficient matrix of all samples based on RNA-seq datasets. **E** Electron micrographs of iPSC-RPE cells, iPRE cells, and ARPE19 cells. **F** Total bound and phagocyted POSs (pointed by arrows) in iPSC-RPE cells, iRPE cells, and ARPE19 cells. **G** Quantification of phagocytosis, as determined by the number of bound and phagocyted POS per field (*n* = 8). **H** Tight junction were demonstrated by ZO-1 immunostaining. **I** TER analysis (*n* = 4). **J** HRP permeability assay (*n* = 4). **K** Expression levels of PEDF and VEGF from upper and lower chambers were determined by ELISA (*n* = 4). Scale bar = 50 μm. Results are expressed as mean ± SD. *P* value measured by one-way ANOVA and post hoc Bonferroni’s test.

Next, we compared the properties of iRPE cells with iPSC-RPE cells and the ARPE19 cell line. ARPE19 is a human RPE cell line which derived spontaneously from a primary culture of RPE cells from a male donor [32], and popularly used as a cell model to clarify the pathogenesis and treatment of AMD [33]. iPSCs were derived from hUCMSCs and differentiated into iPSC-RPE cells in vitro (Fig. S4). iPSC-RPE cells were able to maintain RPE cell morphology within 12 passages, but cell size increased at passage 12 and gradually lost polygonal morphology at the 15th generation (Fig. S4D), thus the iPSC-RPE cells of passages 7–8 were used in this study. We observed that pigments were produced in iPSC-RPE cells, but not in iRPE cells and ARPE19 (Fig. 2A). Compared with iPSC-RPE cells, iRPE cells expressed less RPE markers, CRALBP and TYRP1, but were significantly higher than ARPE19 cells after cells were cultured for 21 days (Fig. 2A–C). iRPE cells did not express FN1 and α-SMA, whereas a small number of iPSC-RPE cells expressed α-SMA, and ARPE19 cells expressed a certain amount of FN1, which was verified by western blotting (Fig. 2A–C). Pearson’s correlation analysis further revealed that the transcriptional profile of iRPE cells was more similar to that of iPSC-RPE cells than to that of ARPE19 cells (Fig. 2D). TEM showed that there were a large number of microvilli on the cell membrane of iPSC-RPE and iRPE cells, while there were almost no microvilli on the membrane of ARPE19 cells (Fig. 2E). POS phagocytosis experiments showed that iPSC-RPE and iRPE cells phagocytized more POS than ARPE19 cells (Fig. 2F, G). Western blotting also showed that iPSC-RPE and iRPE cells expressed higher levels of MERTK (Fig. 2B, C). Immunofluorescence showed that ZO-1 in iPSC-RPE and iRPE cells was continuous, but discontinuous in ARPE19 cells (Fig. 2H). TER and leakage experiments demonstrated that iPSC-RPE and iRPE cells had higher TER and lower leakage compared with ARPE19 cells (Fig. 2I, J), which confirmed that iPSC-RPE and iRPE cells had stronger epithelial cell polarity. The secretion patterns of PEDF and VEGF in iRPE cells were similar as that in iPSC-RPE cells, with a higher concentration of PEDF and a lower concentration of VEGF in the apical media than that in the basal media (Fig. 2K). These results demonstrated that iRPE cells have characteristics comparable to those of iPSC-RPE cells. We further evaluated the senescence of the three types of cultured cells: iPSC-RPE (passage 12), iRPE (passage 35), and ARPE19 (passage 35) by β-Gal staining, and found that both ARPE19 and iRPE cells contained a small part of senescent cells, but more senescent cells were observed among iPSC-RPE cells (Fig. S5).

### iRPE cells possess anti-EMT property

**Fig. 3:**
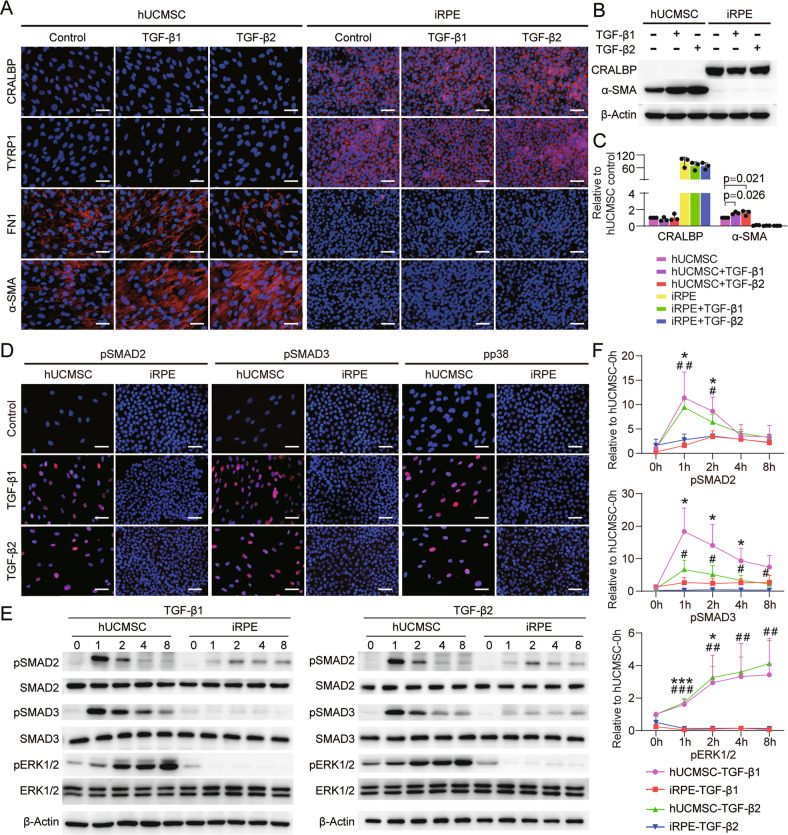
iRPE cells possess anti-EMT property. RPE-specific and EMT-associated markers in hUCMSCs and iRPE cells stimulated with TGF-β1 or TGF-β2 were detected by **A** immunostaining, **B** Western blotting, and **C** quantitative analysis (*n* = 3). The phosphorylation of SMAD2/3, p38, and ERK1/2 in hUCMSCs and iRPE cells stimulated with TGF-β were detected by **D** immunostaining, **E** Western blotting, and **F** quantitative analysis (*n* = 3). Scale bar = 50 μm. Results are expressed as mean ± SD. *P < 0.05, ***P < 0.001, hUCMSC-TGF-β1 compared with iRPE-TGF-β1; ^#^*P* < 0.01, ^##^*P* < 0.01, ^###^*P* < 0.001, hUCMSC-TGF-β2 compared with iRPE-TGF-β2. Results are expressed as mean ± SD. *P* value measured by one-way ANOVA and post hoc Bonferroni’s test.

Clinical evidence demonstrates that RPE cells undergo EMT in AMD, and the level of TGF-β, an inducer of EMT, is elevated in the retina of patients with AMD compared with that in normal control eyes [20–24]. As iRPE cells can be passaged stably in medium containing FBS without losing polygonal morphology, and expressed lower levels of EMT related genes, for example *snai1* and *snai2*, compared with hUCMSCs (Fig. S6), iRPE may have an anti-EMT function. In order to clarify whether iRPE cells can resist EMT induced by TGF-β, we treated iRPE cells and hUCMSCs with TGF- β1 or TGF- β2, the expression of FN1 and α-SMA in iRPE cells was significantly lower than that in hUCMSCs. In addition, there was no significant change in the expression levels of these EMT markers in iRPE cells with or without TGF- β1 or TGF- β2 treatment, and the expression levels of RPE markers CRALBP and TYRP1 were not significantly reduced (Fig. 3A–C). The phosphorylation of SMAD2 and SMAD3 is a TGF-β canonical signaling pathway [34], and immunostaining and western blotting showed that phosphorylation levels of SMAD2 and SMAD3 increased and nuclear retention occurred in hUCMSCs after stimulation by TGF-β1 or TGF-β2 (Fig. 3D–F), indicating that the TGF-β/SMAD pathway was activated in hUCMSCs. However, the nuclear retention of SMAD2 and SMAD3 was inhibited in iRPE cells after TGF-β1 or TGF-β2 stimulation, and phosphorylation were slightly increased (Fig. 3D–F). We next examined the non-canonical TGF-β signaling pathway and found that pp38 was nuclear retention in hUCMSCs, but not in iRPE cells (Fig. 3D–F). In addition, ERK1/2 was activated in hUCMSCs after TGF-β1 or TGF-β2 stimulation, but was not activated in iRPE cells (Fig. 3D–F). These results suggest that iRPE cells can resist the EMT induced by TGF-β1 or TGF-β2. We further compared the EMT resistance capacity of iRPE cells with that of iPSC-RPE cells and ARPE19 cells and found that only iRPE cells possessed anti-EMT ability (Fig. S7).

### PTPN13 confers TGF-β-induced EMT Resistance in iRPE cells

Dephosphorylation is an important function of phosphatases [35]. Blockage of phosphorylation of pSMAD2/3 and ERK1/2 in iRPE cells is likely to be achieved by phosphatases. We detected the expression levels of phosphatases DUSP4, DUSP10, PHLPP1, PPM1L, PPP1CC, PPP2R5A, and PTPN13 in hUCMSCs and iRPE cells and found that the levels of these phosphatases were higher in iRPE cells than those in hUCMSCs (Fig. S8A, B). Next, we applied RNA interference technology to knock down these phosphatases in iRPE cells. Compared with the knockdown of other phosphatases, PTPN13 knockdown more efficiently reduced the RPE marker levels in iRPE cells, and increased the expression levels of EMT markers *fn1* and *α-sma* (Fig. S8C, D), indicating that PTPN13 plays a critical role in maintaining iRPE cell properties. We next demonstrated that overexpression of *crx*, *nr2e1*, or *c-myc* were able to increase the level of *ptpn13* in hUCMSCs, suggesting that these TFs directly regulate the expression of *ptpn13* (Fig. S8E).

**Fig. 4:**
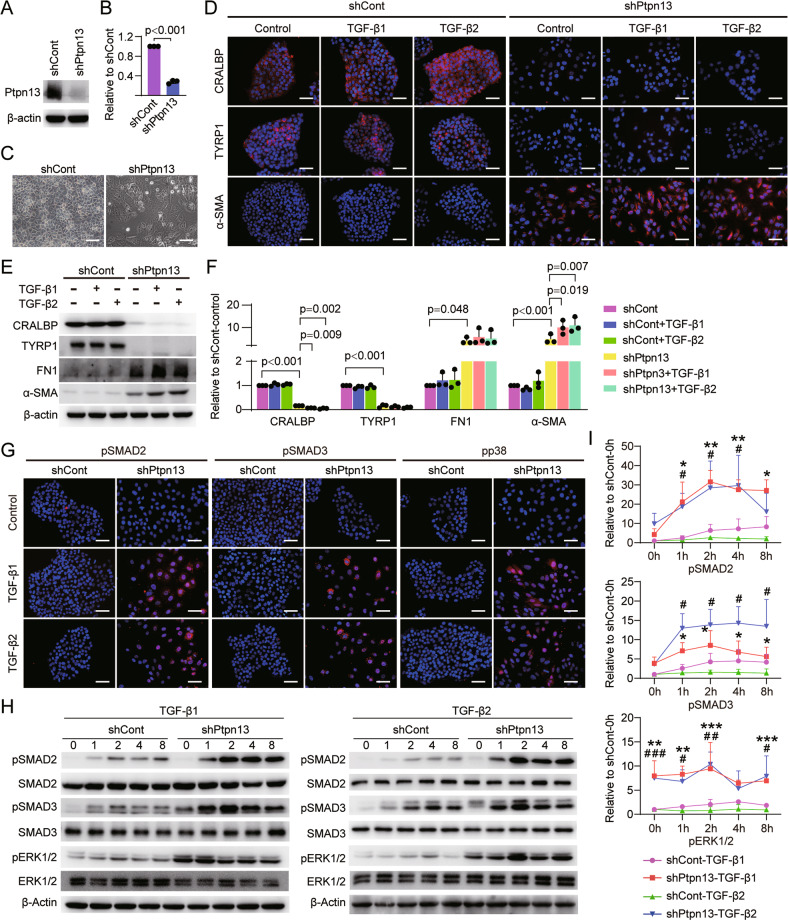
PTPN13 confers TGF-β-induced EMT Resistance on iRPE cells. The expression level of PTPN13 in shCont-iRPE and shPtpn13-iRPE cells were detected by **A** Western blotting and **B** quantitative analysis (*n* = 3, *P* value measured by Student’s unpaired t test). **C** Representative images of shCont-iRPE and shPtpn13-iRPE cells. RPE-specific and EMT-associated markers in shCont-iRPE cells and shPtpn13-iRPE cells stimulated with TGF-β1 or TGF-β2 were detected by **D** immunostaining, **E** Western blotting, and **F** quantitative analysis (*n* = 3, *P* value measured by one-way ANOVA and post hoc Bonferroni’s test). Phosphorylation of SMAD2/3, p38, and ERK1/2 in shCont-iRPE and shPtpn13-iRPE cells were analyzed by **G** immunostaining, **H** Western blotting, and **I** quantitative analysis (*n* = 3, *P* value measured by one-way ANOVA and post hoc Bonferroni’s test). Scale bar = 50 μm. Results are expressed as mean ± SD. *P < 0.05, **P < 0.01, ***P < 0.001, shPtpn13-TGF-β1 compared with shCont-TGF-β1; ^#^*P* < 0.01, ^##^*P* < 0.01, ^###^*P* < 0.001, shPtpn13-TGF-β2 compared with shCont-TGF-β2.

To further clarify whether PTPN13 mediate the inhibition of the TGF-β signaling pathway in iRPE cells, we treated cells with TGF-β. Because shPtpn13-1 in iRPE cells demonstrated higher knockdown efficiency than shPtpn13-2 (Fig. S8C), we selected iRPE cells with shPtpn13-1 deficiency for subsequent experiments and termed them shPtpn13-iRPE cells and confirmed the knockdown efficiency at protein level (Fig. 4A, B). Compared with shCont-iRPE cells, shPtpn13-iRPE cells lost polygonal morphology (Fig. 4C), and when shPtpn13-iRPE cells were stimulated by TGF-β1 or TGF-β2, RPE-related marker CRALBP was significantly downregulated, whereas α-SMA were markedly upregulated, indicating that the TGF-β signaling pathway was activated by TGF-β induction in shPtpn13-iRPE cells (Fig. 4D–F). We next detected the phosphorylation of SMAD2, SMAD3, p38, and ERK1/2, and found that phosphorylation of these molecules and their nuclear retention were significantly increased in shPtpn13-iRPE cells compared with that in shCont-iRPE cells stimulated with TGF-β1 or TGF-β2 (Fig. 4G–I). These results suggest that PTPN13 mediates, at least partly, the anti-EMT function of iRPE cells by inhibiting phosphorylation of the TGF-β signaling pathway.

### PTPN13 dephosphorylates syntenin1 to promote TGF-β receptor internalization and degradation

**Fig. 5:**
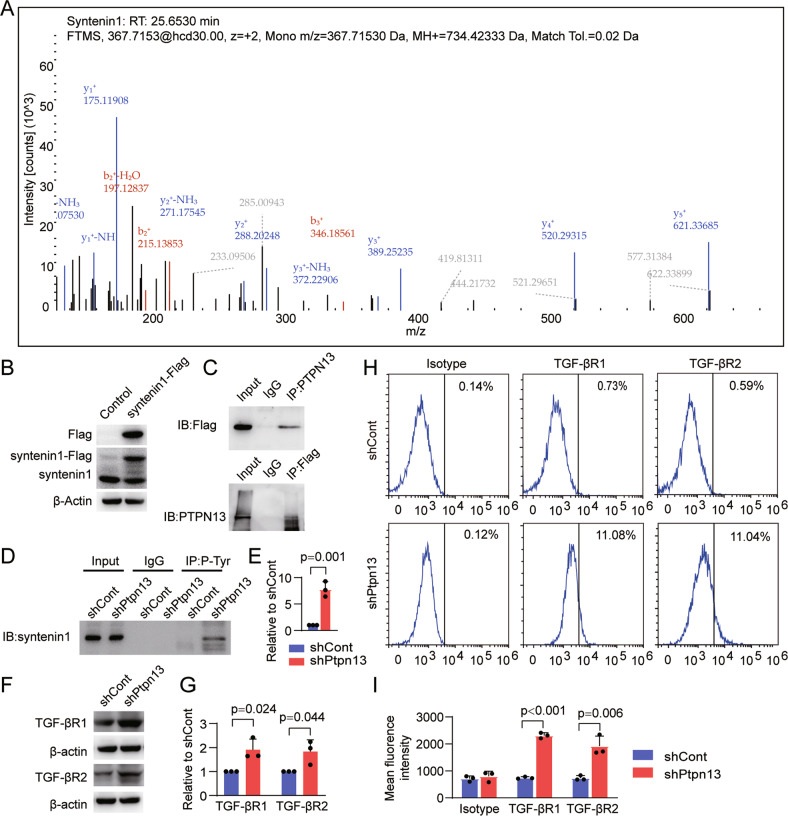
PTPN13 dephosphorylates syntenin1 to promote TGF-β receptor internalization. PTPN13 binding syntenin1 in iRPE cells were identified by **A** mass spectrometry and confirmed by **B**, **C** Western blotting. Phosphorylation of syntenin1 was detected by **D** anti-phosphorylated-Tyr antibody CO-IP assay and **E** quantitative analysis (*n* = 3). The expression levels of TGF-βR1 and TGF-βR2 in shCont-iRPE and shPtpn13-iRPE cells were detected by **F** Western blotting, **G** quantitative analysis (*n* = 3). The expression levels of TGF-βR1 and TGF-βR2 on cell membranes of shCont-iRPE and shPtpn13-iRPE cells were detected by **H** flow cytometry and **I** quantitative analysis of mean fluorescence density (*n* = 3). Results are expressed as mean ± SD. *P* value measured by Student’s unpaired t test.

To clarify the targets of PTPN13, Co-IP test was conducted using an anti-PTPN13 antibody, and proteins binding to PTPN13 in iRPE cells were identified by mass spectrometry. The result showed that syntenin1 is one of the binding targets of PTPN13 (Fig. 5A). We next overexpressed syntenin1-Flag in iRPE cells, and confirmed that PTPN13 binds syntenin1 (Fig. 5B, C). It has been reported that phosphorylated syntenin1 inhibits the TGF-βR internalization and degradation pathway [36]. PTPN13 is very likely to act on syntenin1 and dephosphorylate it, promoting internalization and degradation of TGF-βR1 and TGF-βR2, and subsequently inhibiting the canonical and non-canonical TGF-β signaling pathway. To verify this hypothesis, we performed an anti-phosphorylated-Tyr antibody co-IP assay, and found that the level of phosphorylated syntenin1 in shPtpn13-iRPE cells was significantly higher than that in shCont-iRPE cells (Fig. 5D, E). We further determined the expression levels of TGF-βR1 and TGF-βR2 and found that the total proteins of TGF-βR1 and TGF-βR2 in shPtpn13-iRPE cells were significantly higher than those in shCont-iRPE cells (Fig. 5F, G). Flow cytometry showed that the levels of TGF-βR1 and TGF-βR2 on the cell membrane of shPtpn13-iRPE cells were significantly higher than those on the membrane of shCont-iRPE cells (Fig. 5H, I).

**Fig. 6:**
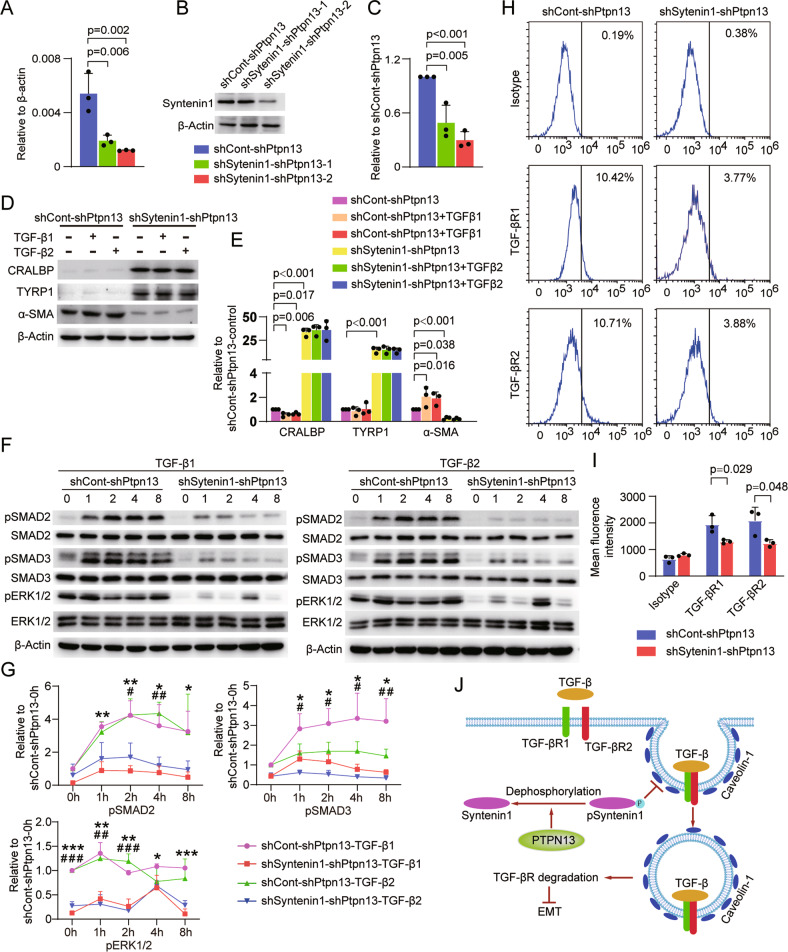
Syntenin1 mediates the internalization of TGF-βR1 and TGF-βR2. Knockdown efficiency of syntenin1 was determined by **A** qRT-PCR (*n* = 3, *P* value measured by one-way ANOVA and post hoc Bonferroni’s test), **B** Western blotting, and **C** quantitative analysis (*n* = 3, *P* value measured by one-way ANOVA and post hoc Bonferroni’s test). RPE-specific and EMT-associated markers in shCont-shPtpn13-iRPE and shSyntenin1-shPtpn13-iRPE cells were analyzed by **D** Western blotting and **E** quantitative analysis (*n* = 3, *P* value measured by one-way ANOVA and post hoc Bonferroni’s test). Phosphorylation of SMAD2/3, p38, and ERK1/2 in shCont-shPtpn13-iRPE and shSyntenin1-shPtpn13-iRPE cells were analyzed by **F** Western blotting and **G** quantitative analysis (*n* = 3, *P* value measured by one-way ANOVA and post hoc Bonferroni’s test). The expression levels of TGF-βR1 and TGF-βR2 on the cell membranes of shCont-shPtpn13-iRPE and shSyntenin1-shPtpn13-iRPE cells were detected by **H** flow cytometry and **I** quantitative analysis of mean fluorescence density (*n* = 3, *P* value measured by Student’s unpaired t test). **J** Schematic model for the PTPN13 dephosphorylating syntenin1 mediates TGF-β receptor internalization and degradation. Results are expressed as mean ± SD. *P < 0.05, **P < 0.01, ***P < 0.001, shCont-shPtpn13-TGF-β1 compared with shSyntenin1-shPtpn13-TGF-β1; ^#^*P* < 0.01, ^##^*P* < 0.01, ^###^*P* < 0.001, shCont-shPtpn13-TGF^-β^2 compared with shSyntenin1-shPtpn13-TGF-β2.

Next, we knocked down syntenin1 in shPtpn13-iRPE cells. shSyntenin1-2 demonstrated higher knockdown efficiency than shSyntenin1-1; therefore, we selected cells with shSyntenin1-2 deficiency for the subsequent experiments and termed them shSyntenin1-shPtpn13-iRPE cells (Fig. 6A–C). Under the induction of TGF-β1 or TGF-β2, the expression levels of RPE markers were further downregulated and EMT markers were upregulated in shCont-shPtpn13-iRPE cells, but did not change in shSyntenin1-shPtpn13-iRPE cells (Fig. 6D, E). Phosphorylation of SMAD2/3 and ERK1/2 was repressed in shSyntenin1-shPtpn13-iRPE cells compared with that in shCont-shPtpn13-iRPE cells (Fig. 6F, G). Furthermore, compared with shCont-shPtpn13-iRPE cells, shSyntenin1-shPtpn13-iRPE cells showed significantly reduced TGF-βR1 and TGF-βR2 expression on cell membrane (Fig. 6H, I). These results collectively suggest that syntenin1 participates in the internalization and degradation of TGF-βR, PTPN13 promotes TGF-βR internalization and degradation by dephosphorylating syntenin1, and subsequently inhibits the EMT of iRPE cells (Fig. 6J).

### iRPE cells demonstrate better therapeutic functions compared with hUCMSCs in rat AMD model

**Fig. 7:**
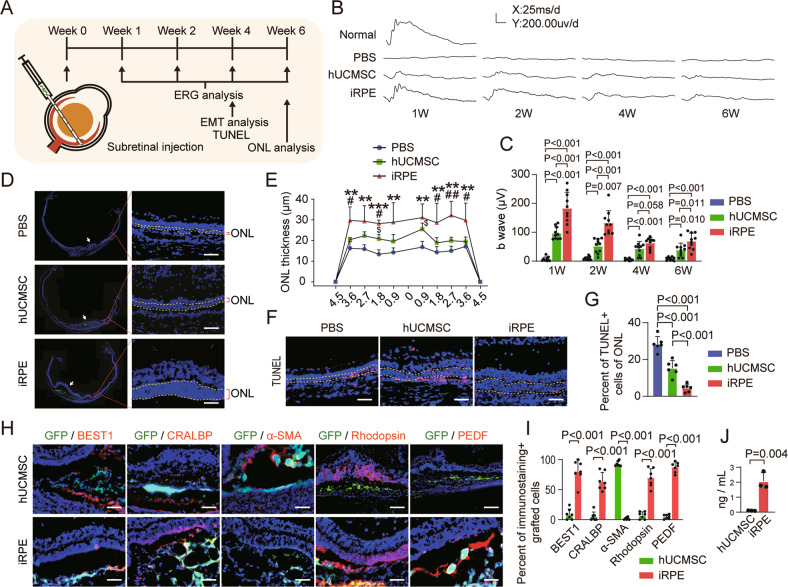
iRPE cells delay retinal degeneration in rat AMD model. **A** Experiment design for iRPE transplantation in SI-induced rat AMD model. **B** ERG waveforms recorded at different time points (the calibration indicates 200 μV vertically and 25 ms horizontally). **C** Quantitative analysis of ERG b-wave amplitude (*n* = 10, *P* value measured by one-way ANOVA and post hoc Bonferroni’s test). **D** Representative micrographs of retinal samples at week 6 post-transplantation. The injection sites were pointed by arrows, and ONL was between yellow dashed lines. **E** Quantitative analysis of ONL thickness (μm) (*n* = 6, *P* value measured by one-way ANOVA and post hoc Bonferroni’s test). **F** Representative micrographs of retinal cryosections stained with TUNEL. **G** Statistical analysis of the percentage of the apoptotic cells in ONL (*n* = 6, *P* value measured by one-way ANOVA and post hoc Bonferroni’s test). **H** Immunostaining of hUCMSCs and iRPE cells after transplantation in vivo for 4 weeks. **I** Quantitative analysis of percent of immunostaining+ cells of grafted cells (*n* = 7, *P* value measured by Student’s unpaired t test). **J** The level of PEDF secreted by hUCMSCs and iRPE cells were determined by ELISA (*n* = 4, *P* value measured by Student’s unpaired t test). Scale bar = 50 μm. Results are expressed as mean ± SD; **P < 0.01, ***P < 0.001, compared with PBS group; ^#^*P* < 0.05, ^##^*P* < 0.01 compared with hUCMSC group; ^$^*P* < 0.05 compared with PBS group.

To verify that iRPE cells maintain RPE characteristics in vivo and possess better therapeutic effects, GFP-labeled iRPE cells or hUCMSCs were injected into the subretinal space of the SI-induced rat AMD model, the experimental design is illustrated in Fig. 7A. The b-wave amplitude was analyzed, and it was almost extinguished in the PBS group at week 1 post-transplantation. The b-wave amplitude of the iRPE cell transplantation group was significantly higher than that of the hUCMSCs and PBS groups at weeks 1 to 6 post-transplantation (Fig. 7B, C). We next analyzed the thickness of the ONL, and at week 6 post-transplantation, there was 1–2 layers of the ONL in the PBS group, compared with 2-3 layers in the hUCMSC group and 5–6 layers in the iRPE cell group (Fig. 7D, E), indicating that subretinal space transplantation of hUCMSCs and iRPE cells could delay the progression of retinopathy in the rat model, and the protective effect of iRPE cells was stronger than that of hUCMSCs. TUNEL staining analysis performed at week 4 post-transplantation showed that the number of apoptotic photoreceptor cells in the ONL in the cell transplantation group was significantly lower than that in the PBS group, especially in the iRPE cell transplantation group (Fig. 7F, G). Immunostaining performed at week 4 post-transplantation showed that nearly no transplanted hUCMSCs were able to express RPE cell markers, whereas iRPE cells still expressed BEST1 and CRALBP, but did not express α-SMA. Furthermore, more iRPE cells phagocytized POS than hUCMSCs, as demonstrated by Rhodopsin+ iRPE cells (Fig. 7H, I). PEDF is thought to enhance the survival of photoreceptors and retinal neurons [37], immunostaining and ELISA showed that iRPE cells express higher level of PEDF than hUCMSCs (Fig. 7H–J). To further evaluate the functions of iRPE cells to delay retinal degeneration, we compared the therapeutic effect of iRPE cells with that of iPSC-RPE cells. iRPE cell transplantation group demonstrated a higher ERG response and thicker ONL compared with that of iPSC-RPE cell transplantation group (Fig. S9). In addition, iRPE cells, but not iPSC-RPE cells, exhibited anti-EMT property (Fig. S9). These results collectively confirmed that iRPE cells maintained their RPE characteristics in vivo and had better therapeutic effects than hUCMSCs and iPSC-RPE cells.

The safety of the iPRE cells was confirmed by subcutaneously inoculating the cells into nude mice. The results showed that the tumor HELA cells formed a large mass after subcutaneous inoculation for 2 weeks (indicated by the arrow in Fig. S10A), suggesting the proliferation of HELA cells, whereas hUCMSCs and iRPE cells did not proliferate even after inoculation for 4 months (Fig. S10A, B). Immunostaining showed that the number of Ki67+ proliferating iRPE cells was significantly lower than that of HELA cells, and is equivalent to the number of proliferating hUCMSCs (Fig. S10C, D), proving that iRPE cells are safe for in vivo transplantation.

## Discussion

The use of key transcription factors to achieve cell lineage transformation has been proven to be an effective strategy [25]. In this study, hUCMSCs were transdifferentiated into iRPE cells by five key TFs: CRX, NR2E1, C-MYC, LHX2, and SIX6. The gene expression patterns and functions of iRPE cells are similar to those of iPSC-RPE cells, and iRPE cells have significantly better therapeutic function than hUCMSCs. In addition, iRPE cells exhibit anti-EMT functions. This study provides a new strategy for the treatment of AMD using iRPE cells with EMT resistance.

Among the five TFs, NR2E1, LHX2, and SIX6 are eye-field TFs (EFTFs) [38]. The neural plate, which is formed in response to neural inducers such as noggin, generates regions that develop into the eye [39]. LHX2 is expressed at an early stage and participates in eye field specification within the presumptive forebrain [40]. NR2E1 and SIX6 play a role in the late specification of eye formation [41, 42]. CRX is thought to be the key TF for photoreceptor cell fate determination [43], and C-MYC plays an important role in the control of cell proliferation, growth, differentiation, apoptosis, survival, and stem cell self-renewal [44]. The combination of the five TFs was able to transdifferentiate hUCMSCs into iRPE cells, indicating that the five TFs may participate in the development of RPE cells. LHX2 regulates the expression of visual cycle genes in RPE cells [45]. c-myc expression is higher in adult RPE cells than in fibroblasts and the neural retina derived from the same human eye donors. In addition, Zhang et al. demonstrated that C-MYC is essential for the direct conversion of fibroblasts into RPE-like cells [46]. Therefore, LHX2 and C-MYC are essential for maintaining mature RPE function. Further study is needed to clarify the mechanisms by which these five TFs cooperate to transdifferentiate hUCMSCs into iRPE cells.

Studies have demonstrated that MSCs have therapeutic effects in animal models of AMD [9–13]. However, the differentiation of MSCs into RPE cells in animal models is still controversial [14, 15]. Although MSCs have a powerful paracrine function [47], secreted factors, including growth factors and cytokines, are different from those secreted by RPE cells; for example, iPSC-RPE and iRPE cells secreted more PEDF than hUCMSCs in this study. Furthermore, MSCs do not have phagocytic functions and are unable to participate in the light cycle. Therefore, transplantation of RPE cells should in theory be better than that of MSCs. Although RPE cells derived from pluripotent stem cells have been used in clinical trials for the treatment of AMD patients and have achieved intriguing outcomes [3–8], MSCs still have advantages for clinical application; for example, MSCs are easy to obtain and safe after transplantation in vivo. In particular, hUCMSCs are routinely stored in cell banks, and a considerable number of newborn babies have their own UCMSCs stored in cell banks that may be used for autologous transplantation in the future. We confirmed that transplantation of iRPE cells derived from hUCMSCs markedly improved therapeutic outcomes in an animal model of AMD, which will enhance the clinical application value of hUCMSCs.

Besides possessing similar characteristics as iPSC-RPE cells, the reason for iRPE cells demonstrating a stronger therapeutic effect than hUCMSCs may be due to the anti-EMT function. It is well documented that some RPE cells appear to degenerate by losing polygonal morphology, are often multilayered, and migrate into the retina and sub-RPE space in both dry and wet AMD [20]. A closer examination of these “degenerating” cells revealed that some cells do not die but may transform into mesenchymal cells to survive the harsh microenvironment during disease progression [21, 22]. Retinal TGF-β levels have been reported to be high in AMD patients [23, 24]. In an animal model of CNV, TGF-β1 and TGF-β2 were significantly increased, and the degree of fibrosis was remarkably reduced after injection of the TGF-β antibody [48]. These studies suggest that TGF-β-mediated EMT may play a critical role in the loss of RPE phenotype in AMD. The iRPE cells in our study were resistant to TGF-β-induced EMT in vitro and maintained their RPE characteristics in vivo, suggesting that the development of EMT-resistant RPE cells is a novel strategy for the treatment of AMD based on RPE cell transplantation.

TGF-β binding to its receptors not only activates the classical signaling pathway involving recruitment and phosphorylation of SMAD2/3 and translocation into the nucleus to trigger the expression of EMT-related genes, but also activates non-canonical TGF-β signaling, including phosphorylation of MAPKs, ERK, p38, and PI3K [34]. Suppressing the TGF-β signaling pathway can inhibit the EMT process of RPE cells; for example, suppressing the phosphorylation of TGF-βR1 can inhibit the EMT of RPE cells [49], and blocking the ERK/AKT signaling pathway can also inhibit the EMT of RPE cells [50]. Protein phosphatases reverse the actions of protein kinases and thus inactivate substrates, which provides a balance for the proper function of signaling molecules. For instance, phosphatase PP1 can dephosphorylate TGF-βR1 and inhibit the TGF-β signaling pathway [51]. PP2A can dephosphorylate SMAD3 under hypoxic conditions [52], while PPM1A targets SMAD2 and reduces the phosphorylation of SMAD2 [53]. Because both the canonical and non-canonical TGF-β signaling pathways are repressed in iRPE cells, the mechanism may include multiple phosphatases dephosphorylating different targets in TGF-β signaling pathways and/or TGF-βR dephosphorylation or degeneration. We confirmed that several phosphatase deficiencies led to the increased expression of EMT markers in iRPE cells, and PTPN13 showed the most powerful regulatory effect in iRPE cells.

PTPN13 is a tyrosine phosphatase that is frequently downregulated or silenced in several tumors. In addition, it is thought to be an independent prognostic marker for increased overall survival in several cancers [54]. Further evidence demonstrated that PTPN13 overexpression inhibited cell invasiveness through cell junction stabilization [55]. PTPN13 can dephosphorylate ERK and AKT in tumor cells and inhibit breast cancer by directly dephosphorylating SRC [56–58]. In this study, we confirmed that PTPN13 directly targets and dephosphorylates syntenin1. Syntenin1 is a 32-kDa protein comprising tandem PDZ (PSD-95/Dl/ZO-1) domains [59]. Previous studies have shown that syntenin1 participates in multiple biological functions including receptor clustering, protein trafficking, and exosome biogenesis [60, 61]. Syntenin1 can prevent caveolin-1-mediated internalization and subsequent degradation of TGF-βRI, thus promoting TGF-β1-activated SMAD signaling and EMT in cancer cells [36]. PTPN13 dephosphorylates and inactivates syntenin1, which promotes internalization of TGF-βR, thus repressing the canonical and non-canonical TGF-β signaling pathways and subsequently inhibiting EMT of iRPE cells. This study suggests that PTPN13 is an important target for inhibiting EMT in RPE cells.

A limitation of this study is that we used retroviruses to randomly integrate the five TFs into the chromosomes of hUCMSCs for reprogramming the cells into iRPE cells. Although we did not observe tumorigenesis after transplantation of iRPE cells in nude mice, there are still risks with regards to future clinical use. The best method may be to integrate the key TF genes into the AAVS1 (also known as PPP1R12C) locus, a well-validated “safe harbor” in the human genome, to avoid tumorigenicity or death caused by random integration [62]. Furthermore, because lots of the grafted cells died 6 weeks post-transplantation, an appropriate immunosuppression scheme is required to prolong the survival of grafted cells in animal models.

In summary, the five TFs (CRX, NR2E1, C-MYC, LHX2, and SIX6) successfully transdifferentiated hUCMSCs into iRPE cells, which exhibited comparable properties to iPSC-RPE cells and demonstrated better therapeutic function than hUCMSCs in a rat model of AMD. In addition, highly expressed PTPN13 in iRPE cells endows cells with EMT-resistant capacity by dephosphorylating syntenin1 and subsequently promoting TGF-βR internalization and degeneration. hUCMSC-derived iRPE cells may be promising candidates for clinical use to reverse AMD pathophysiology and ultimately restore vision in the future. Furthermore, this strategy of endowing grafted cells with anti-EMT functions may be used to treat other EMT-related diseases, such as pulmonary and renal fibrosis.

## Supplementary information

Supplementary figure legends

Supplementary figure 1

Supplementary figure 2

Supplementary figure 3

Supplementary figure 4

Supplementary figure 5

Supplementary figure 6

Supplementary figure 7

Supplementary figure 8

Supplementary figure 9

Supplementary figure 10

Supplementary tables

Original figures of western blotting

Checklist

## Data Availability

All data generated or analyzed during this study are included in this published article and in its supplementary information file.
