# Supplementary material for: Direct conversion of human umbilical cord mesenchymal stem cells into retinal pigment epithelial cells for treatment of retinal degeneration
Source: Cell Death Dis. 2022 Sep 12;13(9):785. doi: 10.1038/s41419-022-05199-5 (PMC9468174; doi:10.1038/s41419-022-05199-5)
Supplement: Supplementary file 1 — Supplementary figure legends [file 41419_2022_5199_MOESM1_ESM.docx]

**Supplementary figure 1. Characterization of hUCMSCs.** **A** Morphology (Bright field), adipogenesis (Oil-red-O), osteogenesis (AKP) and chondrogenesis (Toluidine blue) of hUCMSCs. **B** Flow cytometry analysis of cell membrane markers on hUCMSCs. Scale bar = 50 μm.

**Supplementary figure 2. Five TFs are essential and sufficient to transdifferentiate hUMSCs into iRPE cells.** **A** Total clones from hUCMSCs cells transfected with the combinations of 8 TFs were stained with giemsa staining. **B** Representative image of iRPE clone. Scale bar = 50 μm. **C** The efficiency of iRPE generation was calculated by the number of RPE-like clones per 100,000 hUCMSCs cells. **D** Different combinations of TFs were used to transdifferentiate hUCMSCs into iRPE cells. The expression levels of exogenous TFs were determined by qRT-PCR (n = 3). **E** RPE-specific and EMT-associated markers were detected by qRT-PCR (n = 3, *P* value measured by one-way ANOVA and post hoc Bonferroni’s test). Results are expressed as mean ± SD.

**Supplementary figure 3. Phagocytosis of POSs by hUMSCs and iRPE cells.** Representative images were taken by confocal microscope. Scale bar = 10 μm.

**Supplementary figure 4. hUCMSCs are reprogrammed into iPSCs and further differentiated into iPSC-RPE cells.** **A** iPSCs showed normal pluripotent stem cell morphology. **B** iPSCs expressing pluripotent markers OCT4, NANOG, SOX2, and SSEA4 was identified by immunostaining. **C** Pigmented iPSC-RPE cells appeared in iPSC-formed embryonic bodies. **D** The pigmented areas were isolated by a surgical blade and cultured to form a monolayer in which cells demonstrated polygonal morphology. The cells were further subcultured to passages 8, 12, and 15. Scale bar = 50 μm.

**Supplementary figure 5.** Senescence of iPSC-RPE cells, ARPE19, and iRPE. iPSC-RPE cells (passage 12), iRPE (passage 35), and ARPE19 (passage 35) were cultured in 12-well culture plate. β-Gal staining was performed. A Representative image of of senescent cells. B Quantitative analysis of senescent cells. (n = 8). Results are expressed as mean ± SD. *P* value measured by one-way ANOVA and post hoc Bonferroni’s test.

**Supplementary figure 6. Gene expressions in hUMSCs and iRPE cells identified by RNA-seq analysis.** **A** Volcano plot of up-regulated (red) and down-regulated (green) differentially expressed genes (DEGs) in hUMSCs and iRPE cells. **B** Gene ontology (GO) enrichment analysis of the DEGs between hUMSCs and iRPE cells based on the GO database, EMT-related genes were enriched. **C** Heat map of the EMT-related genes.

**Supplementary figure 7.** **iRPE cells rather than iPSC-RPE cells and ARPE19 cells possess anti-EMT function. A-C** RPE-specific and EMT-associated markers in iPSC-RPE cells, iRPE cells, and ARPE19 cells stimulated with TGF-β1 or TGF-β2 were detected by (**A**) immunostaining, (**B**) Western blotting and (**C**) quantitative analysis (n = 3). Scale bar = 50 μm. Results are expressed as mean ± SD. *P* value measured by one-way ANOVA and post hoc Bonferroni’s test.

**Supplementary figure 8. PTPN13 is the key phosphatase for endowing iRPE cells with EMT Resistance. A, B** The expression levels of phosphatases in hUCMSCs and iRPE cells were detected by (**A**) Western blotting and (**B**) quantitative analysis (n = 3, *P* value measured by Student’s unpaired t test). **C** Knockdown efficiency of phosphatases was determined by qRT-PCR (n = 3, *P* value measured by one-way ANOVA and post hoc Bonferroni’s test). **D** RPE-specific and EMT-associated markers in shCont-iRPE cells and shPhosphatase-iRPE cells were detected by qRT-PCR (n = 3, *P* value measured by Student’s unpaired t test). **E** the expression level of *ptpn13* in hUCMSCs with overexpression of *crx, nr2e1, c-myc, lhx2,* or *six6* was detected by qRT-PCR (n = 3, *P* value measured by one-way ANOVA and post hoc Bonferroni’s test). Results are expressed as mean ± SD.

**Supplementary figure 9. iRPE cells demonstrate better therapeutic functions than iPSC-RPE cells. A** ERG waveforms recorded at different time points (the calibration indicates 200 μV vertically and 25 ms horizontally). **B** Quantitative analysis of ERG b-wave amplitude (n = 10). **C** Representative micrographs of retinal samples at week 6 post-transplantation. The injection sites were pointed by arrows, and ONL was between yellow dashed lines. **D** Quantitative analysis of ONL thickness (μm) (n = 7). **E** Immunostaining of iPSC-RPE cells and iRPE cells after transplantation *in vivo* for 4 weeks. *P* value measured by one-way ANOVA and post hoc Bonferroni’s test

**Supplementary figure 10. Tumorigenesis assay for iRPE in nude mice.** **A** HELA, hUCMSCs, and iRPE cells were subcutaneously inoculated into nude mice. **B** Tumorigenesis rate of cells in nude mice. (J) Proliferation of the inoculated cells, as assessed by Ki67 immunostaining and (K) quantitative analysis (n = 7). Scale bar = 50 μm. Results are expressed as mean ± SD. *P* value measured by one-way ANOVA and post hoc Bonferroni’s test.
