# Supplementary figures and images for: Direct conversion of human umbilical cord mesenchymal stem cells into retinal pigment epithelial cells for treatment of retinal degeneration
Source: Cell Death Dis. 2022 Sep 12;13(9):785. doi: 10.1038/s41419-022-05199-5 (PMC9468174; doi:10.1038/s41419-022-05199-5)

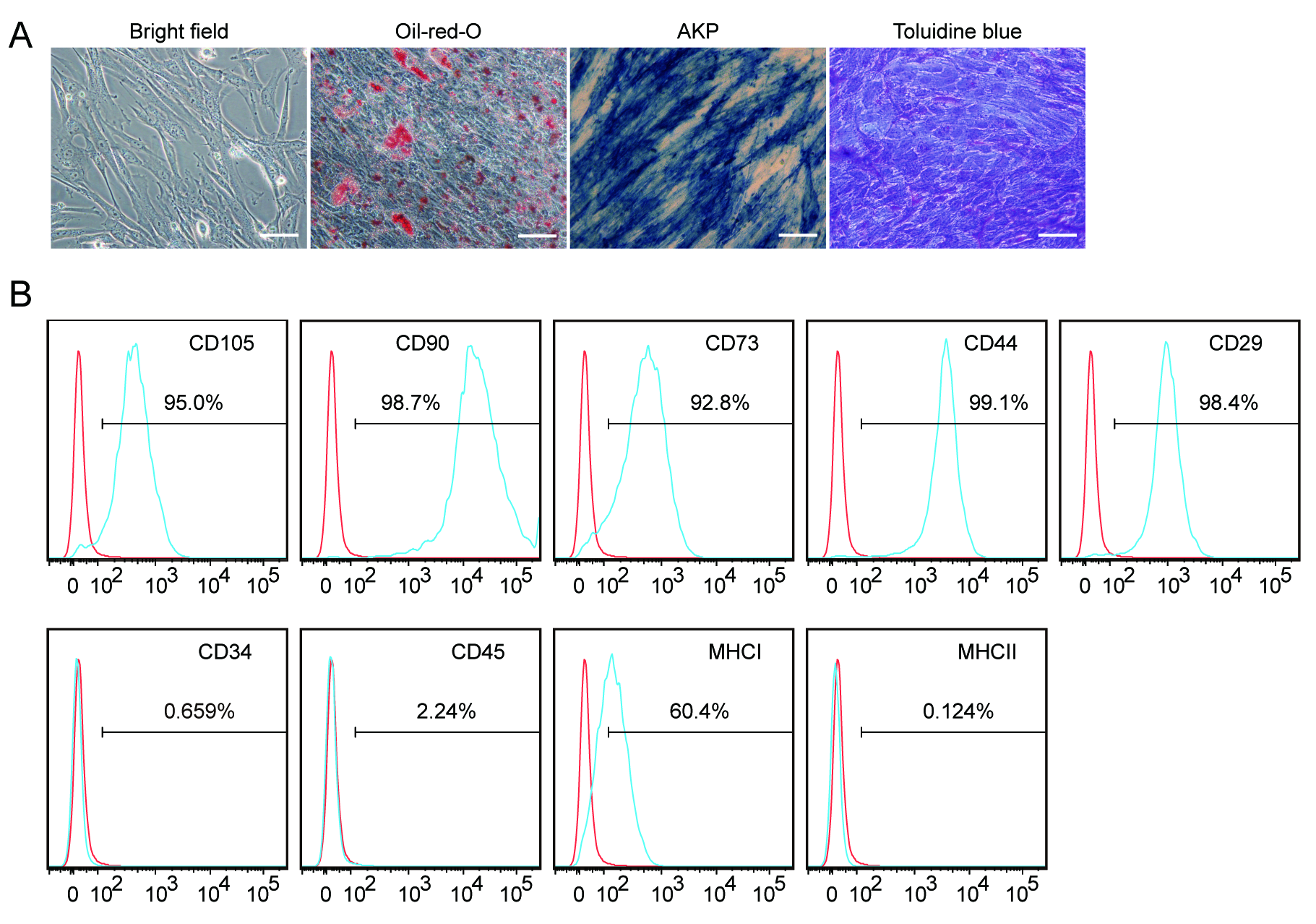

Supplement: Supplementary file 2 — Supplementary figure 1 [file 41419_2022_5199_MOESM2_ESM.tif]

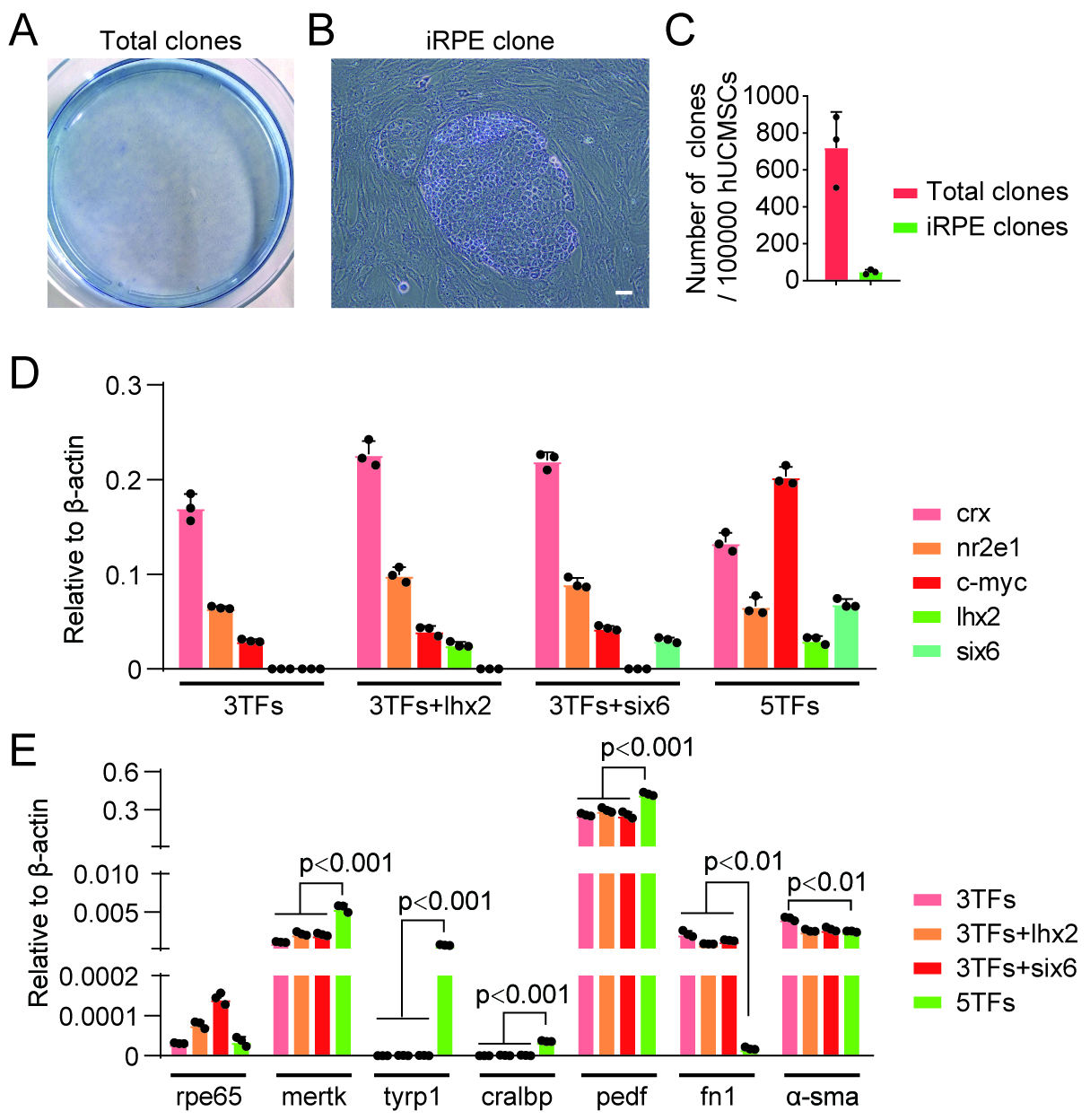

Supplement: Supplementary file 3 — Supplementary figure 2 [file 41419_2022_5199_MOESM3_ESM.tif]

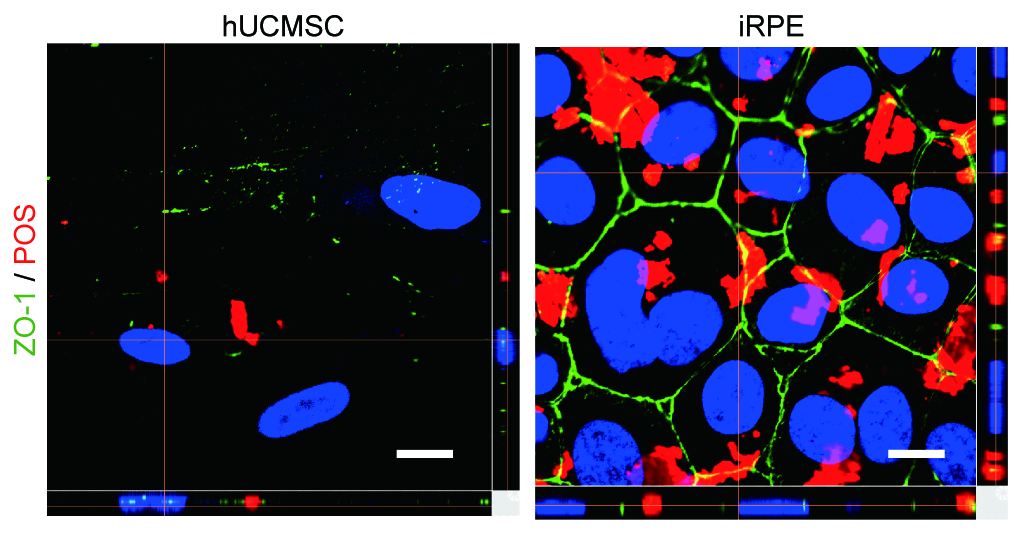

Supplement: Supplementary file 4 — Supplementary figure 3 [file 41419_2022_5199_MOESM4_ESM.tif]

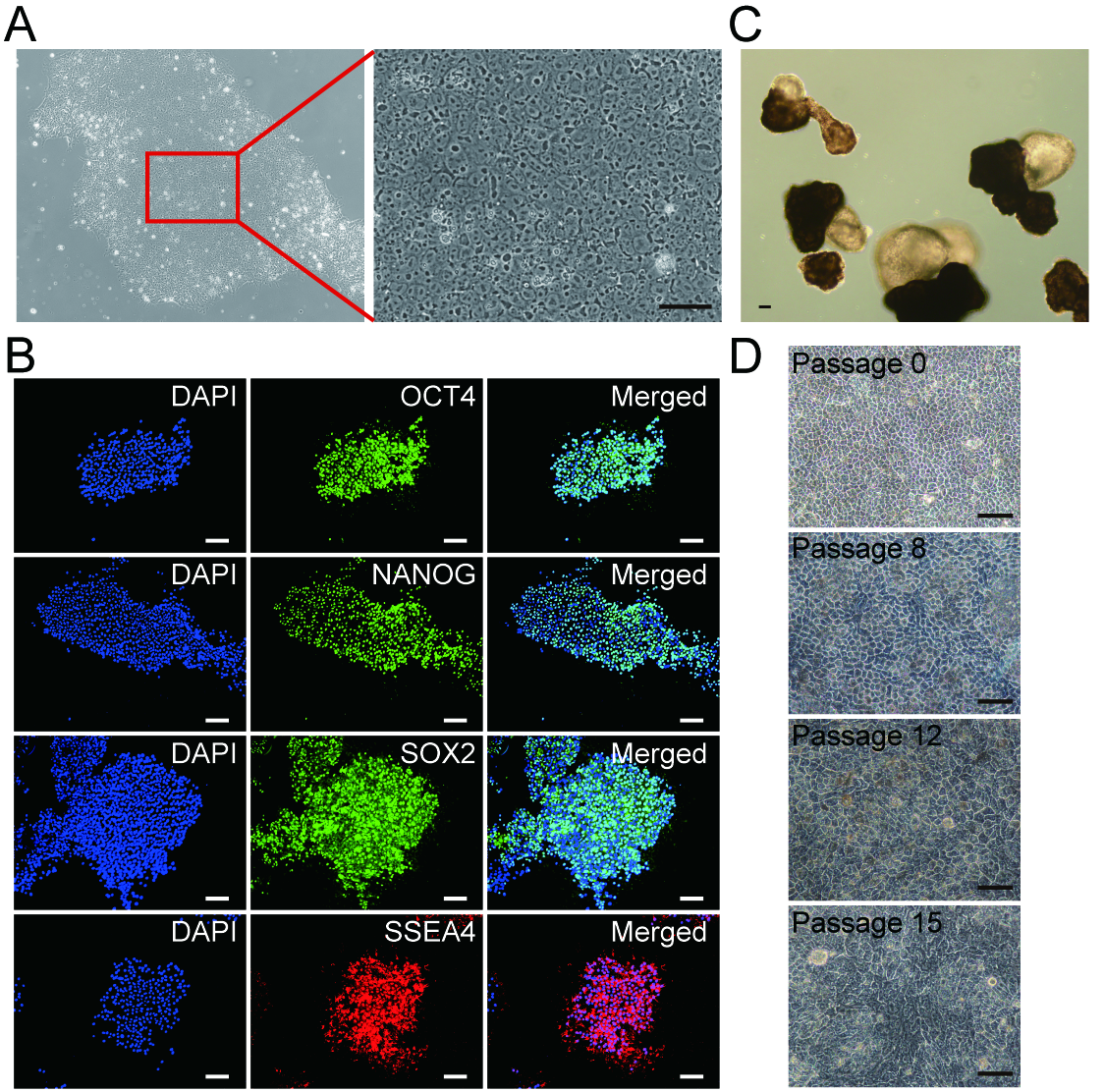

Supplement: Supplementary file 5 — Supplementary figure 4 [file 41419_2022_5199_MOESM5_ESM.tif]

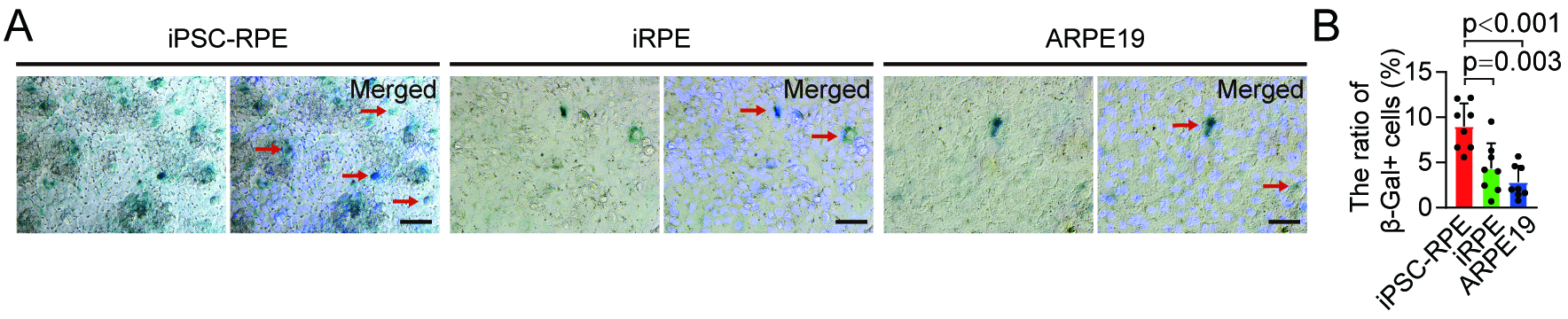

Supplement: Supplementary file 6 — Supplementary figure 5 [file 41419_2022_5199_MOESM6_ESM.tif]

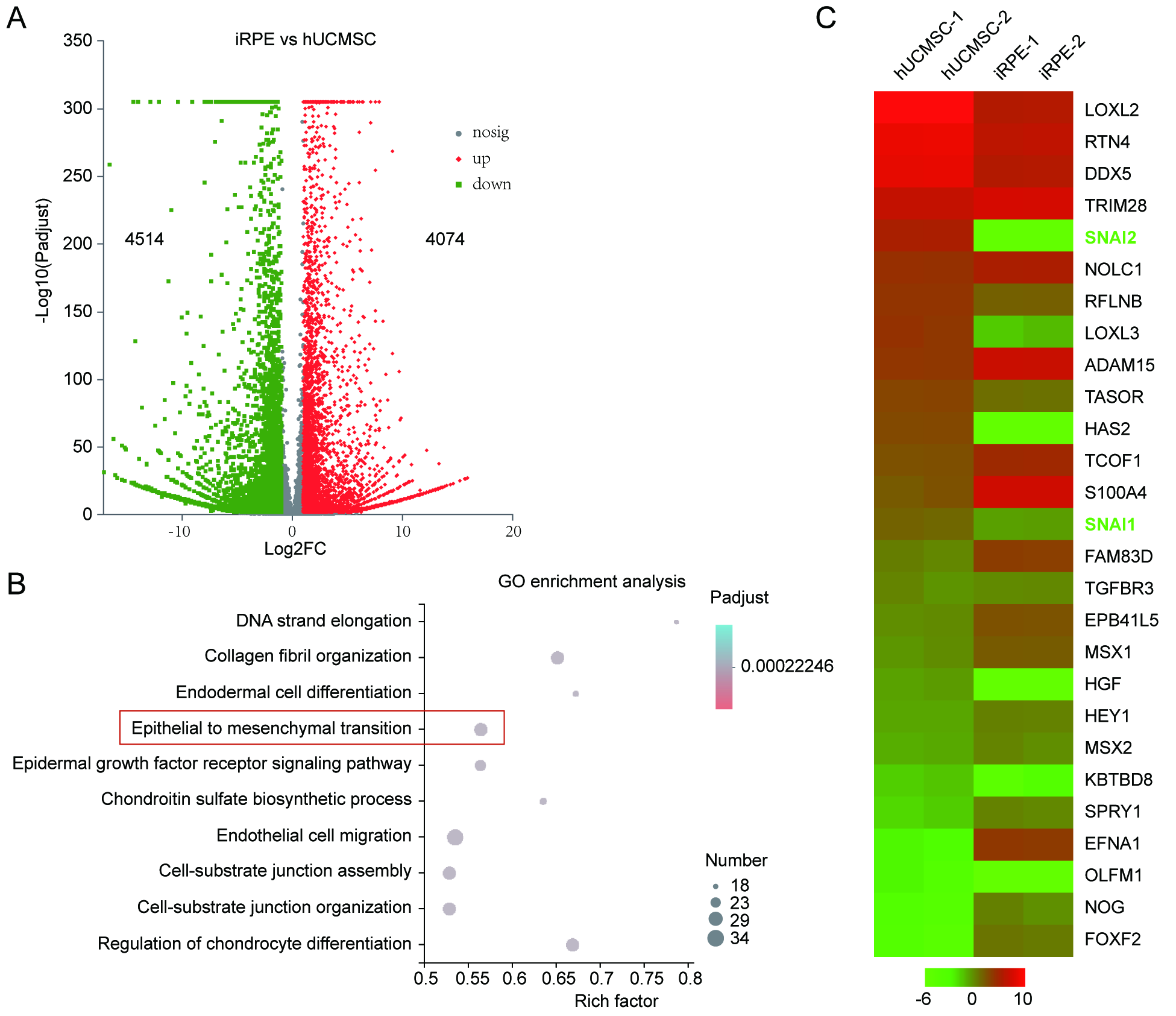

Supplement: Supplementary file 7 — Supplementary figure 6 [file 41419_2022_5199_MOESM7_ESM.tif]

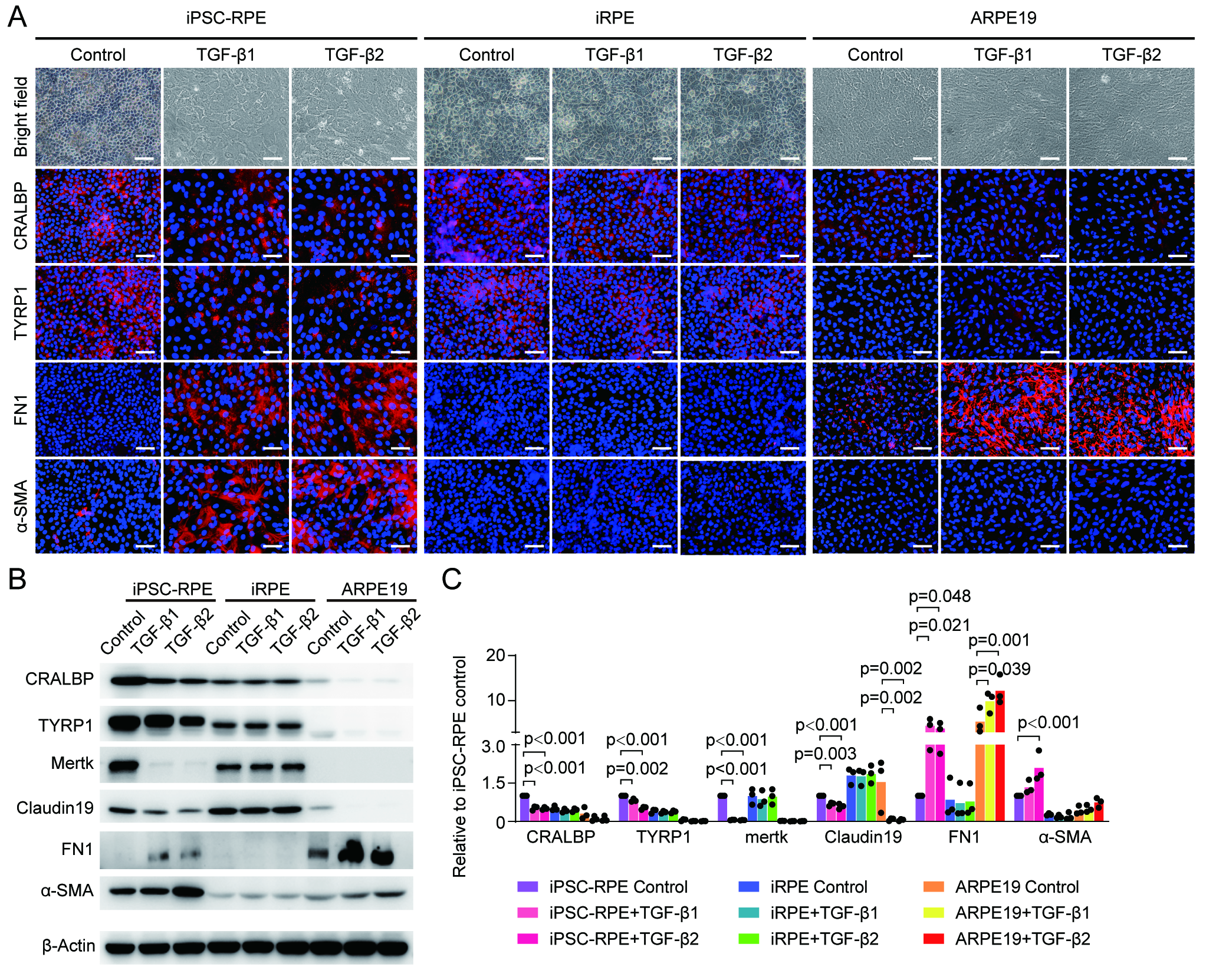

Supplement: Supplementary file 8 — Supplementary figure 7 [file 41419_2022_5199_MOESM8_ESM.tif]

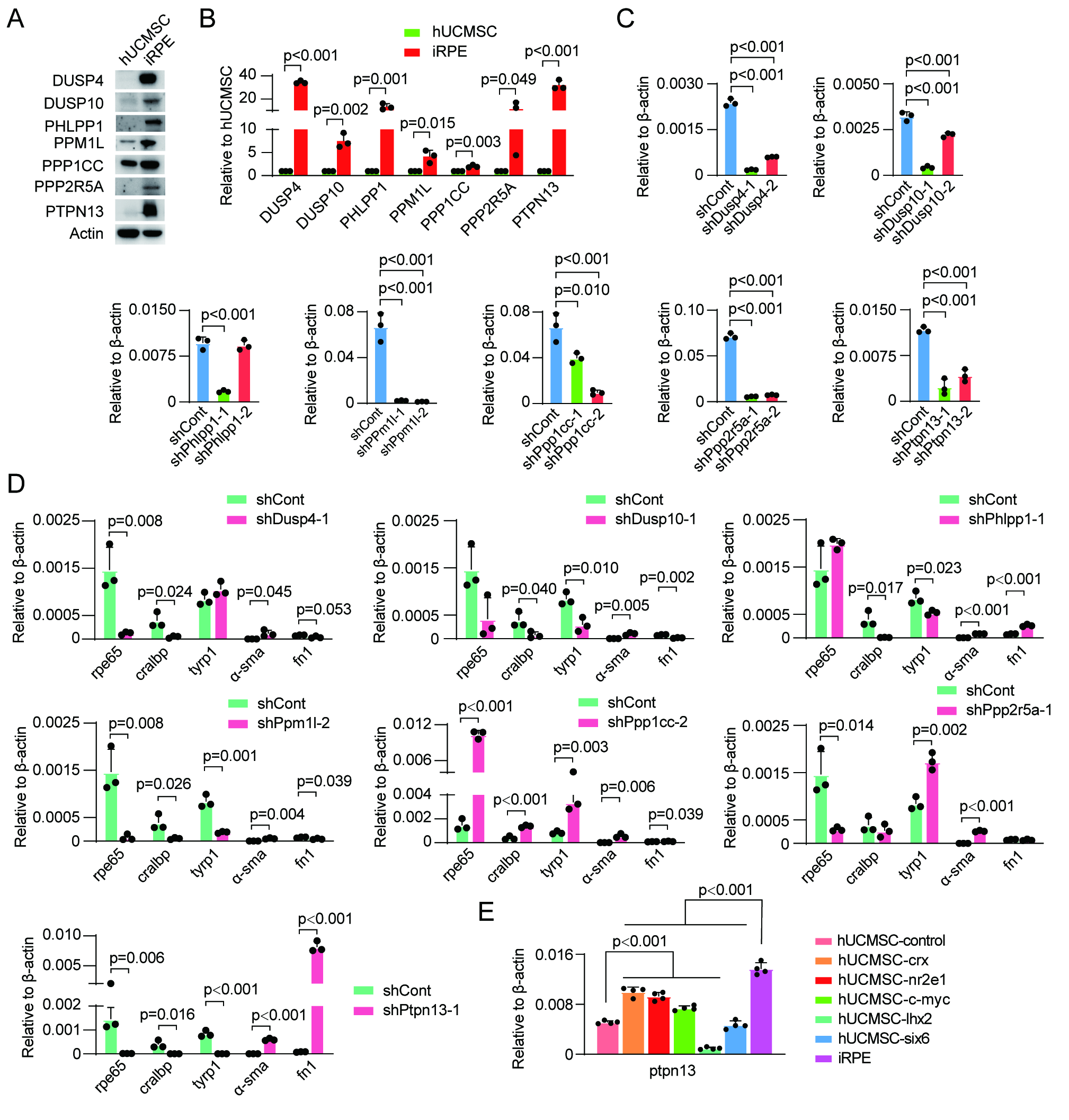

Supplement: Supplementary file 9 — Supplementary figure 8 [file 41419_2022_5199_MOESM9_ESM.tif]

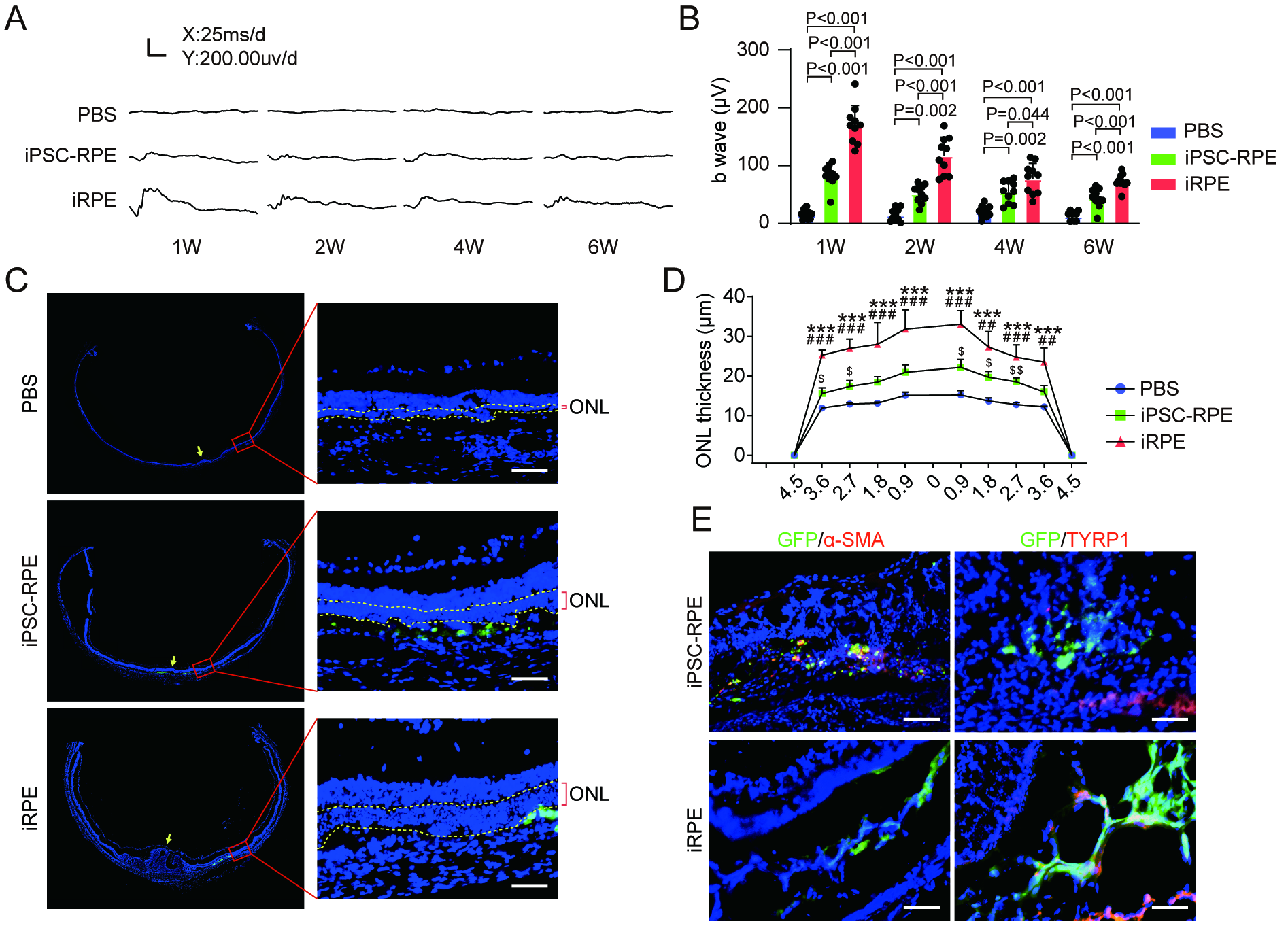

Supplement: Supplementary file 10 — Supplementary figure 9 [file 41419_2022_5199_MOESM10_ESM.tif]

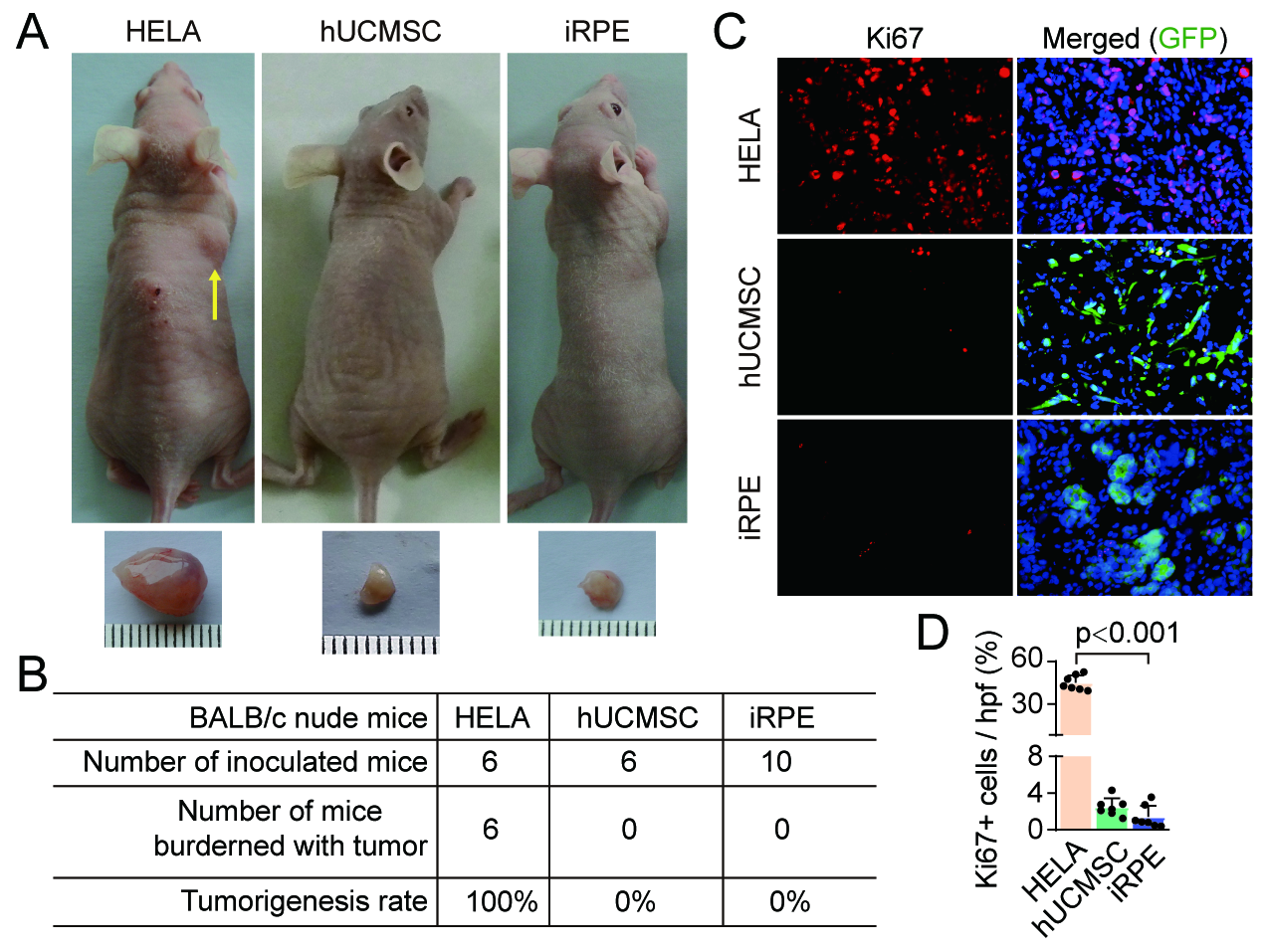

Supplement: Supplementary file 11 — Supplementary figure 10 [file 41419_2022_5199_MOESM11_ESM.tif]
