# Supplementary material for: Direct conversion of human umbilical cord mesenchymal stem cells into retinal pigment epithelial cells for treatment of retinal degeneration
Source: Cell Death Dis. 2022 Sep 12;13(9):785. doi: 10.1038/s41419-022-05199-5 (PMC9468174; doi:10.1038/s41419-022-05199-5)
Supplement: Supplementary file 12 — Supplementary tables [file 41419_2022_5199_MOESM12_ESM.docx]

**Supplemental Table 1. Primers for the analysis of Expression Levels of RPE Specific Genes, EMT-related Genes and phosphatase Genes.**

| Genes | Forward sequence (5’-3’) | Reverse sequence (5’-3’) | Accession no. |
| --- | --- | --- | --- |
| pedf | CTCATCAGCCACTGGAAAG | TCTGAACATGTCGGTCATTC | NM_001386460.1 |
| cralbp | GACAGGAAGTCACAACTTGG | TGCCTATGGAAGACACAGA | NM_000326.5 |
| mertk | AGCAATAATCGCTTCCTTCA | ATGGGATCAGACACGATCT | NM_006343.3 |
| rpe65 | CCAGGACTCTTTGAAGTTGG | GGATGAACCTTCTGTGGTATG | NM_000329.3 |
| tyrp1 | GACCAGAGGGTTCTCATAGT | TGCGCTTTGCCATATCC | NM_000550.3 |
| fn1 | GAGCTGCACATGTCTTGGGAAC | GGAGCAAATGGCACCGAGATA | NM_212482.4 |
| α-sma | GGGACATCAAGGAGAAACTG | CAGGCAACTCGTAACTCTTC | NM_001141945.2 |
| β-actin | ACTCTTCCAGCCTTCCTT | GTACAGGTCTTTGCGGATG | NM_001101.5 |
| dusp4 | CTGGTTCATGGAAGCCATAG | CGTTTCTTCATCATCAGGTAGG | NM_001394 |
| dusp10 | CCTGAAGAGAGAAGGCAAAG | GGAGTTGTCACAGAGGTTT | NM_144728 |
| phlpp1 | TGCCTCATCCGCTTCTAT | CACTGGCAACTGCATCTT | NM_194449 |
| ppm1l | CGTCCATCTTCGGGATCT | CCTCTGGGAGTCGAGATTT | NM_139245 |
| ppp1cc | TCGACAGCATTATCCAACG | AAGCACAGTCCTCTGATTTC | NM_002710 |
| ppp2r5a | AGCAACACTGAATGAACTGG | GGAAGTGTACGGAAGATGTTAG | NM_001199756 |
| ptpn13 | TTATTGCTGGCATCCTTGG | CAAGTTTCTCCATCACTCTGG | NM_080685 |
| syntenin1 | CTGCTCCTATCCCTCACGATG | GGCCACATTTGCACGTATTTCT | NM_001006800.1 |

**Supplemental Table 2. Primers for the Analysis of Exogenous TF Genes.**

| Genes | Forward sequence (5’-3’) | Reverse sequence (5’-3’) |
| --- | --- | --- |
| c-myc | GGACATCCTCTAGACTGCCG | AGAAATACGGCTGCACCGAG |
| nr2e1 | CCATCCTCTAGACTGCCGGA | CGTAGACCCCGTAGTGCTTC |
| crx | GGGTGGACCATCCTCTAGACT | ATCCACACTGGGGCCACTTA |
| mitf-a | TCATGAAACTCCTCCCCGAC | GAAATCCGGCACGATCCCC |
| sox2 | AAGGATCCCAGTGTGGTGGT | GCTTCAGCTCCGTCTCCATC |
| klf4 | GTGTGGTGGTACGGGAAATCA | CTGGACGCAGTGTCTTCTCC |
| lhx2 | GACCATCCTCTAGACTGCCG | CATCTCGTCGATGACCCCG |
| six6 | GGGTGGACCATCCTCTAGACT | CACTTGCTGGGGGCTGAAAT |

**Supplemental Table 3. Antibodies for Immunostaining and Western Blotting.**

| Antibody | Company and Cat NO. |
| --- | --- |
| ZO-1 | Proteintech, 21773-1-AP |
| FN1 | Proteintech, 66042-1-Ig |
| BEST1 | Proteintech, 21910-1-AP |
| CRALBP | Proteintech,15356-1-AP |
| syntenin1 | Proteintech, 22399-1-AP |
| DUSP4 | Proteintech, 66349-1-Ig |
| DUSP10 | Proteintech, 11689-1-AP |
| PHLPP1 | Proteintech, 22789-1-AP |
| PPM1L | Proteintech, 18203-1-AP |
| PPP1CC | Proteintech, 11082-1-AP |
| PPP2R5A | Proteintech, 12675-2-AP |
| PTPN13 | Proteintech, 25944-1-AP |
| TGF-βR2 | Proteintech, 27212-1-AP |
| β-Actin | Proteintech, 66009-1-Ig |
| Goat anti-mouse IgG (H+L), HRP conjugate | Proteintech, SA00001-1 |
| Goat Anti-Rabbit IgG(H+L), HRP conjugate | Proteintech, SA00001-2 |
| MERTK | Abcam, ab52968 |
| Rhodopsin | Abcam, ab98887 |
| TGF-βR1 | Abcam, ab235578 |
| TYRP1 | Abcam, ab235447 |
| α-SMA | Abcam, ab5694 |
| SMAD2 | Abcam, ab33875 |
| pSMAD2 | Abcam, ab53100 |
| pSMAD3 | Abcam, ab52903 |
| SMAD3 | Abcam, ab40854 |
| Ki67 | Abcam, ab92742 |
| PEDF | Abcam, ab233120 |
| pERK1/2 | CST, 9101 |
| ERK1/2 | CST, 9102 |
| pp38 | CST, 9216 |
| Phospho-Tyrosine MultiMab Rabbit mAb | CST, 8954s |
| RPE65 | Novus Biologicals, NB100-355 |
| Claudin19 | Invitrogen, A5-101526 |
| Goat anti-Mouse IgG (H+L) Secondary Antibody, Alexa Fluor Plus 555 | Invitrogen, A32727 |
| Goat anti-Rabbit IgG (H+L) Secondary Antibody, Alexa Fluor Plus 488 | Invitrogen, A32731 |
| Donkey anti-Rabbit IgG (H+L) Secondary Antibody, Alexa Fluor Plus 555 | Invitrogen, A32794 |
| FLAG | MBL International, M185-3L |

**Supplemental Table 4. The shRNA Sequences for Target Genes.**

| shRNA | Sequence |
| --- | --- |
| shDusp4-1 | CCGGCGCAGTTCGTCTTCAGC |
| shDusp4-2 | CCGGGGAGGCCTTCGAGTTCG |
| shDusp10-1 | CCGGCAATGAACCAAGCCGAG |
| shDusp10-2 | CCGGCCACTATGAGAAAGGCC |
| shPhlpp1-1 | CCGGCGAGGTCTTTCCCGAAG |
| shPhlpp1-2 | CCGGCCTGATAGTATCATCTG |
| shPpm1l-1 | CCGGCGTGTTTGATTGCTCTG |
| shPpm1l-2 | CCGGGTGACAAAGATGGGAAC |
| shPpp1cc-1 | CCGGCAAATGCCACGAGACCT |
| shPpp1cc-2 | CCGGGCGAATTATGCGACCAA |
| shPpp2r5a-1 | CCGGCACTGAATGAACTGGTT |
| shPpp2r5a-2 | CCGGCGACCATTGTAGCACTG |
| ShPtpn13-1 | CCGGCCAGTGAAAGTCCATCT |
| ShPtpn13-2 | CCGGGCCACGGTCTATTCTTA |
| shSyntenin1-1 | GCCAAGTTGGAGACCAGAATC |
| shSyntenin1-2 | GAAAGAAGGATCGCCGCATCA |
| shCont | GCGCGATAGCGCTAATAATTT |
