# Supplementary material for: Direct conversion of human umbilical cord mesenchymal stem cells into retinal pigment epithelial cells for treatment of retinal degeneration
Source: Cell Death Dis. 2022 Sep 12;13(9):785. doi: 10.1038/s41419-022-05199-5 (PMC9468174; doi:10.1038/s41419-022-05199-5)
Supplement: Supplementary file 13 — Original figures of western blotting [file 41419_2022_5199_MOESM13_ESM.pdf]

Figure 1F.

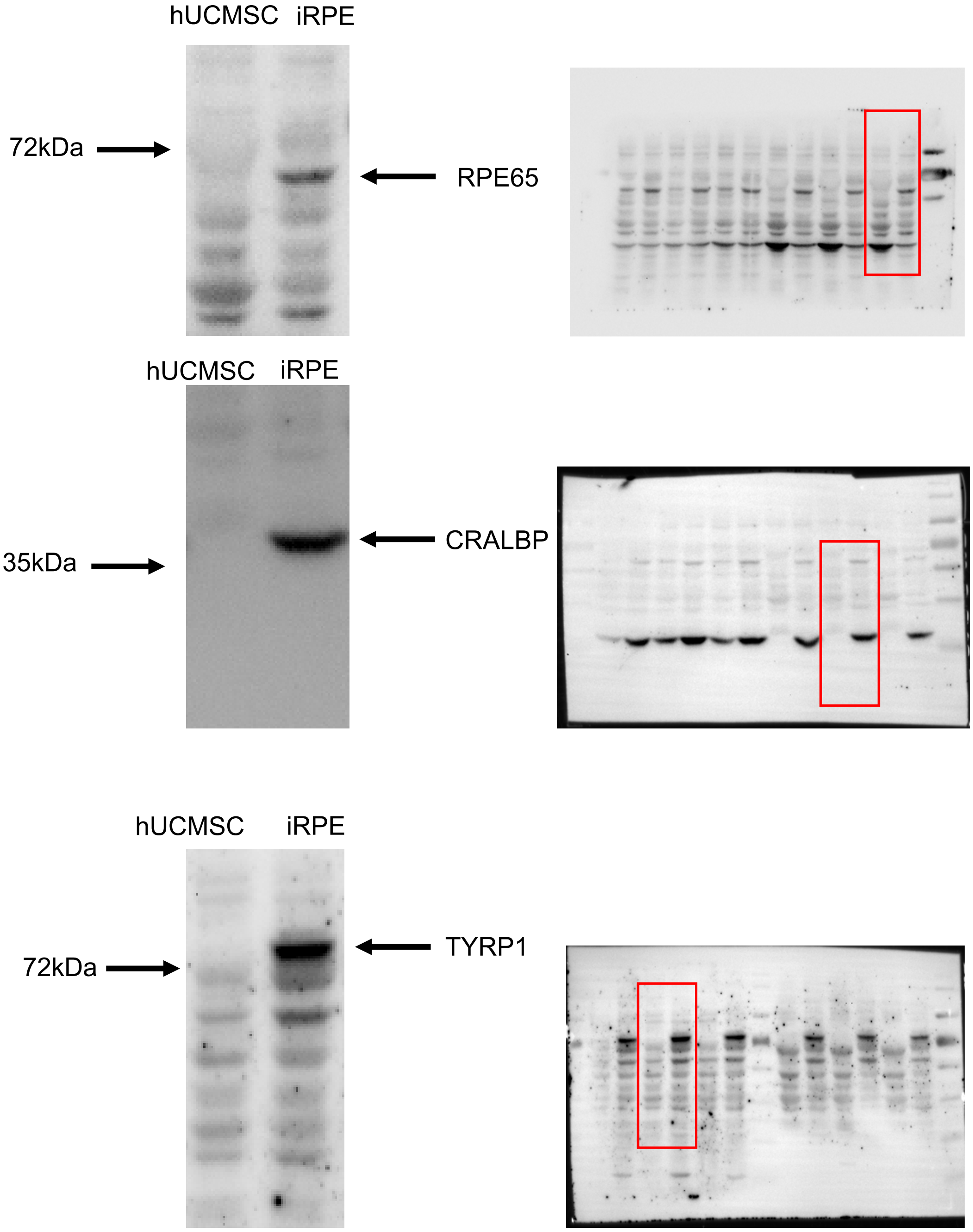
hUCMSC iRPE
72kDa
RPE65
hUCMSC iRPE
35kDa
CRALBP
hUCMSC iRPE
72kDa
TYRP1

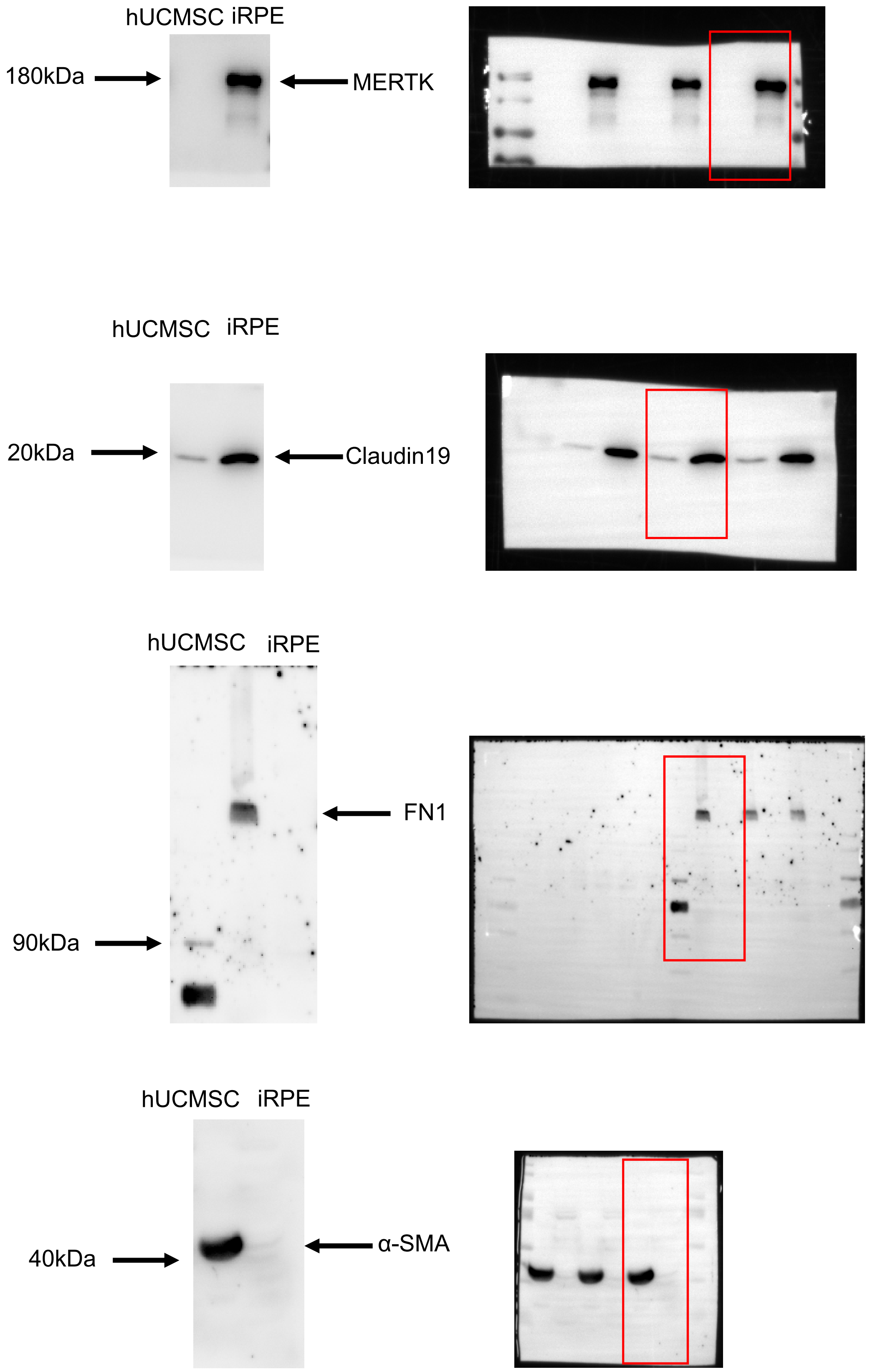
hUCMSC iRPE
180kDa
MERTK
hUCMSC iRPE
20kDa
Claudin19
hUCMSC iRPE
FN1
90kDa
hUCMSC iRPE
40kDa
α-SMA

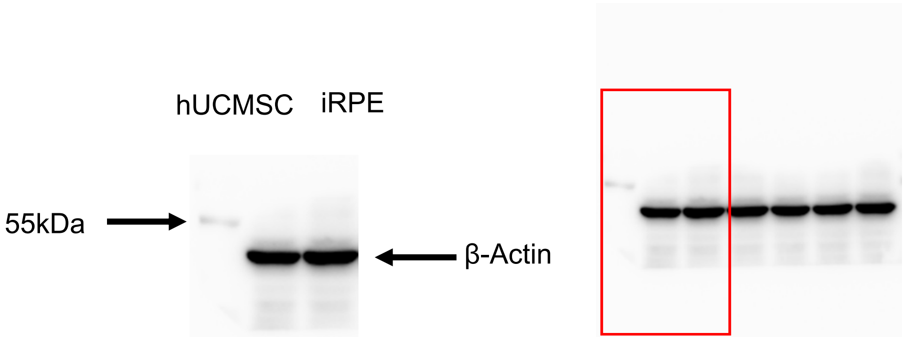
hUCMSC iRPE
55kDa
β-Actin

Figure 2B.

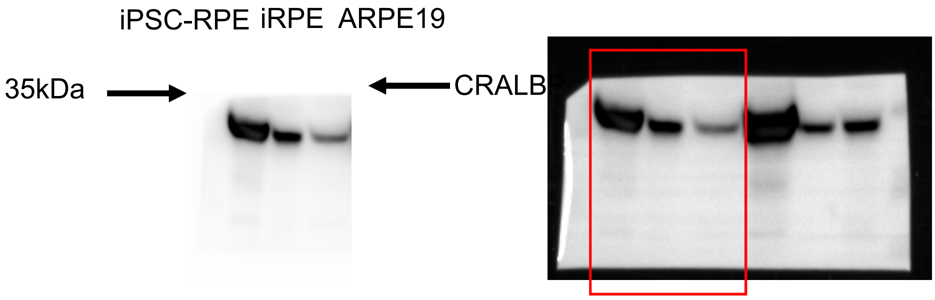
iPSC-RPE iRPE ARPE19
35kDa
CRALBP

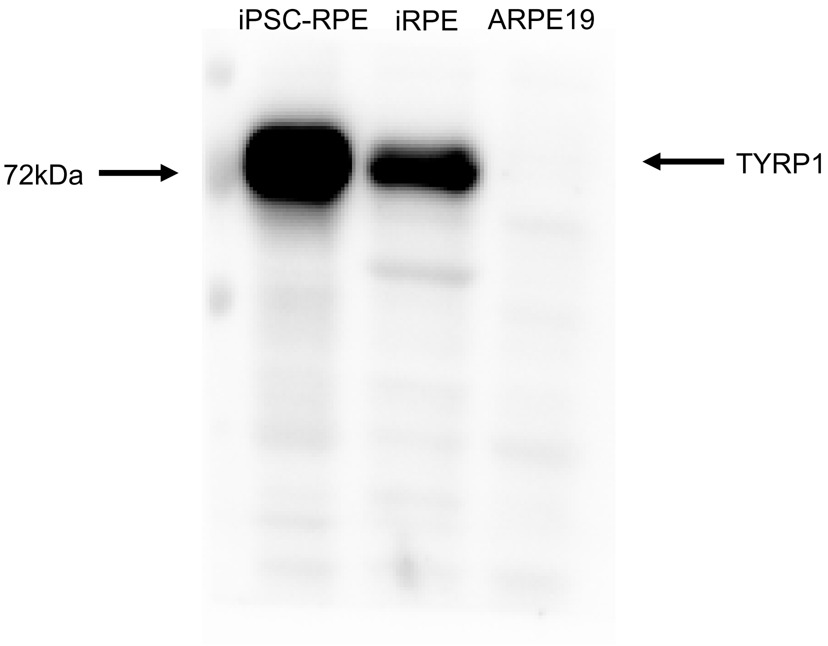
iPSC-RPE iRPE ARPE19
72kDa
TYRP1

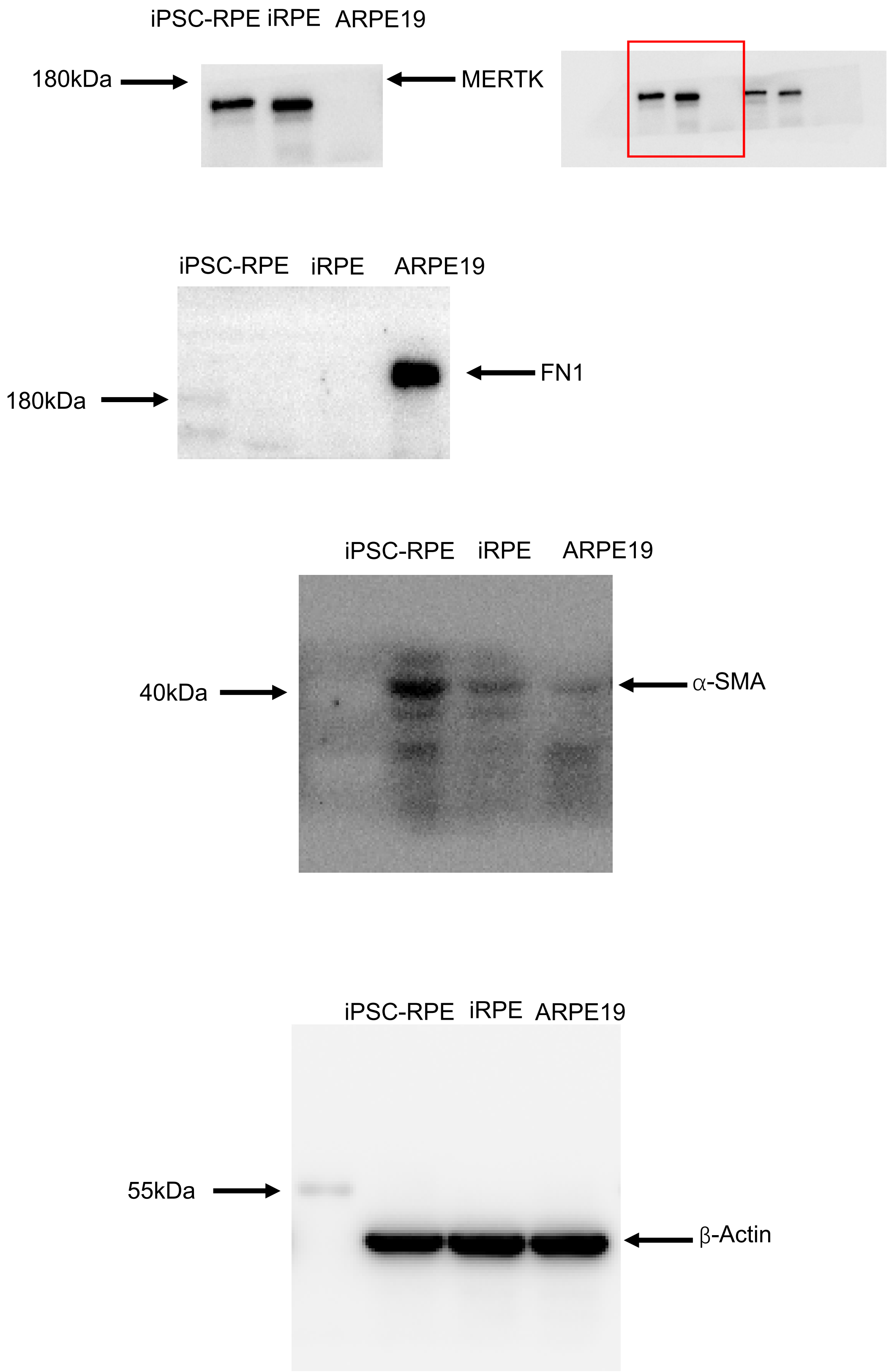
iPSC-RPE iRPE ARPE19
180kDa
MERTK
iPSC-RPE iRPE ARPE19
180kDa
FN1
iPSC-RPE iRPE ARPE19
40kDa
α-SMA
iPSC-RPE iRPE ARPE19
55kDa
β-Actin

Figure 3B.

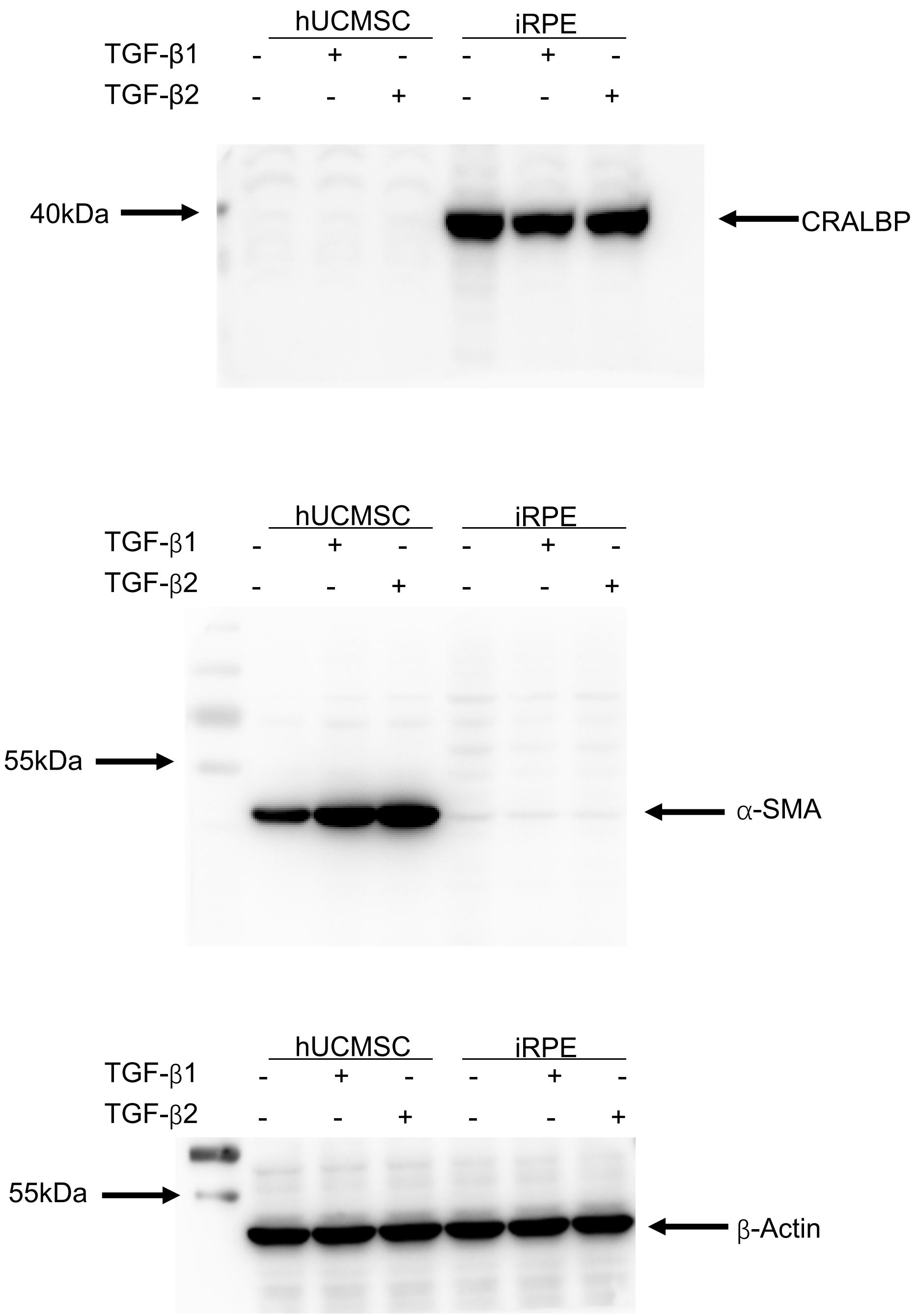
hUCMSC
iRPE
TGF-β1 - + - - + -
TGF-β2 - - + - - +
40kDa
CRALBP
hUCMSC
iRPE
TGF-β1 - + - - + -
TGF-β2 - - + - - +
55kDa
α-SMA
hUCMSC
iRPE
TGF-β1 - + - - + -
TGF-β2 - - + - - +
55kDa
β-Actin

Figure 3E.

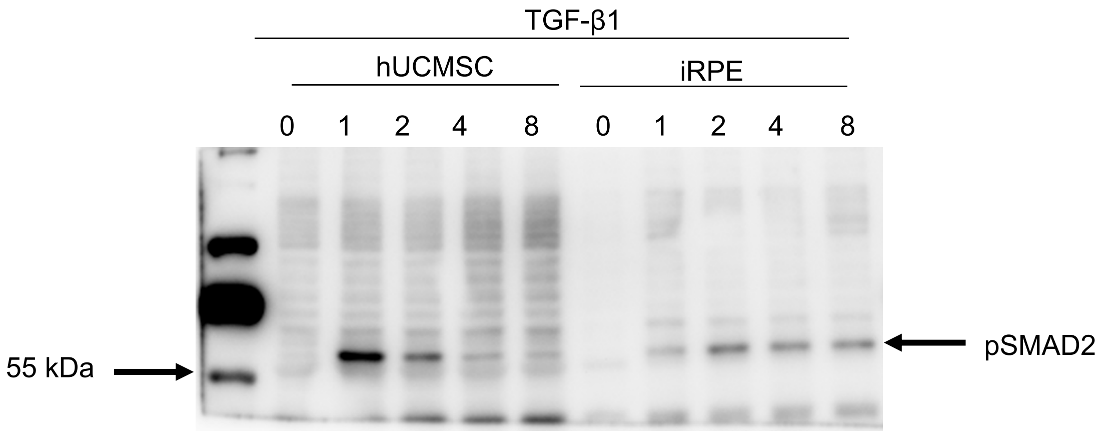
TGF-β1
hUCMSC
iRPE
0 1 2 4 8 0 1 2 4 8
55 kDa
pSMAD2

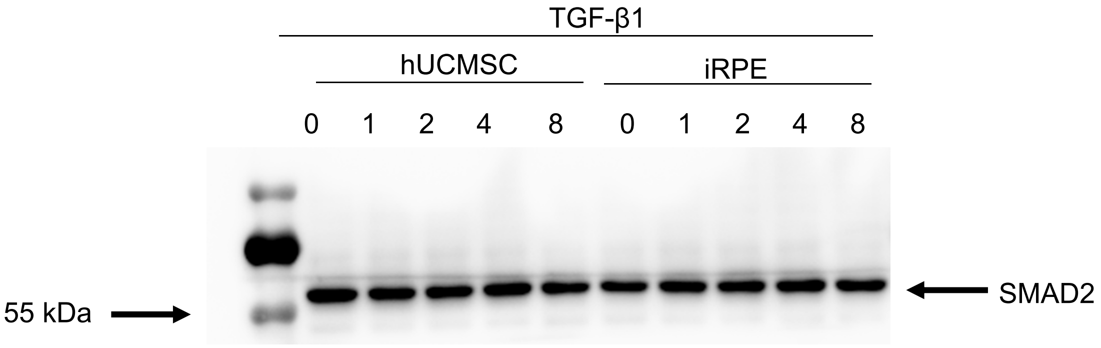
TGF-β1
hUCMSC
iRPE
0 1 2 4 8 0 1 2 4 8
55 kDa
SMAD2

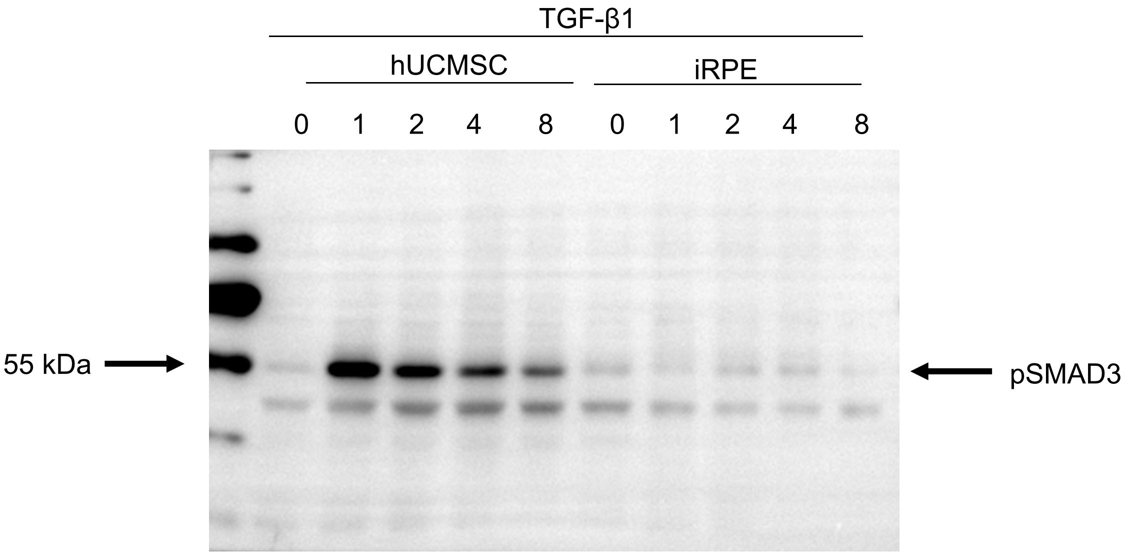
TGF-β1
hUCMSC
iRPE
0 1 2 4 8 0 1 2 4 8
55 kDa
pSMAD3

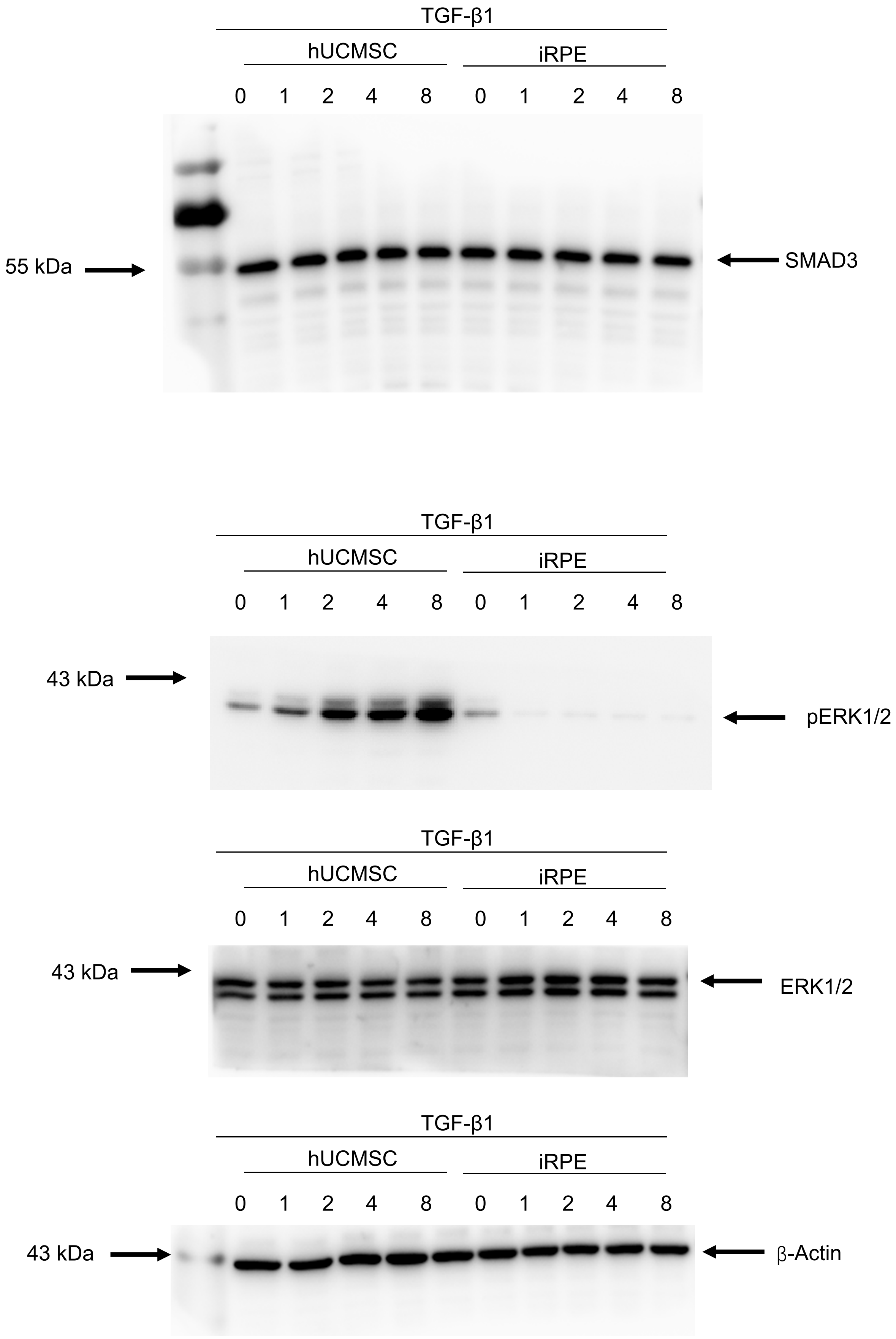
TGF-β1
hUCMSC
iRPE
0 1 2 4 8 0 1 2 4 8
55 kDa
SMAD3
TGF-β1
hUCMSC
iRPE
0 1 2 4 8 0 1 2 4 8
43 kDa
pERK1/2
TGF-β1
hUCMSC
iRPE
0 1 2 4 8 0 1 2 4 8
43 kDa
ERK1/2
TGF-β1
hUCMSC
iRPE
0 1 2 4 8 0 1 2 4 8
43 kDa
β-Actin

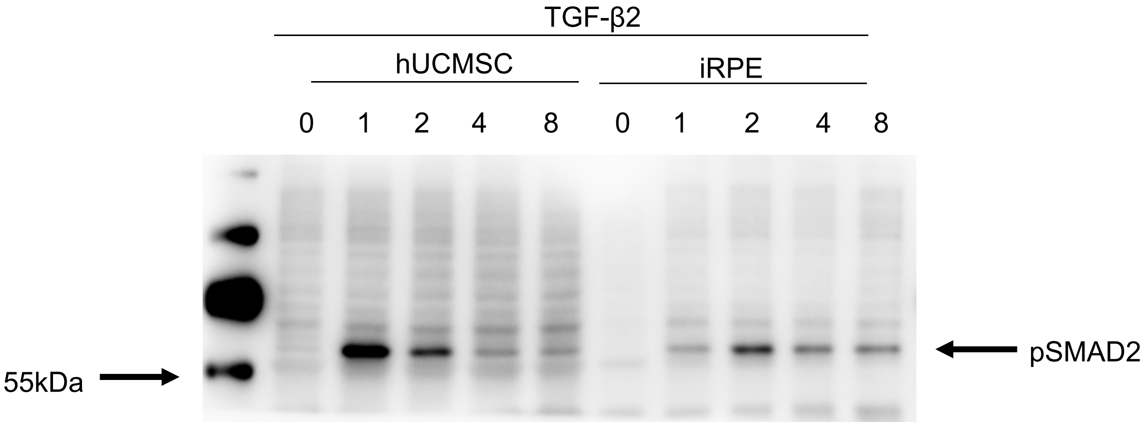
TGF-β2
hUCMSC
iRPE
0 1 2 4 8 0 1 2 4 8
55kDa
pSMAD2

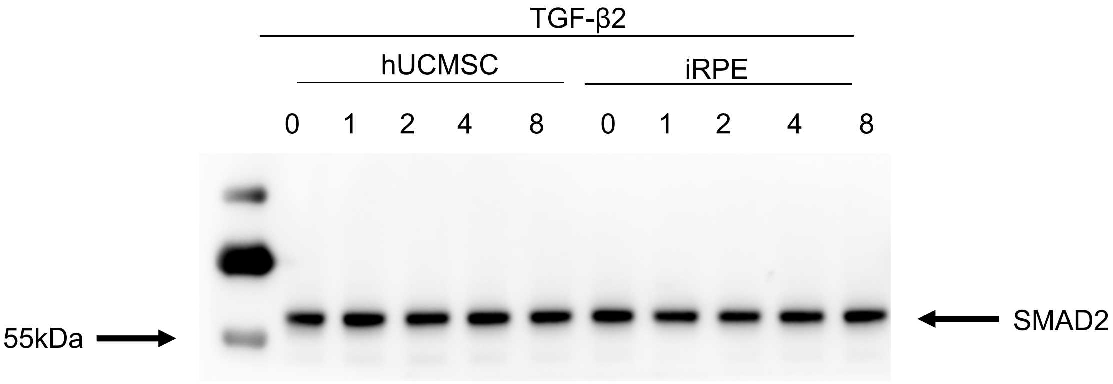
TGF-β2
hUCMSC
iRPE
0 1 2 4 8 0 1 2 4 8
55kDa
SMAD2

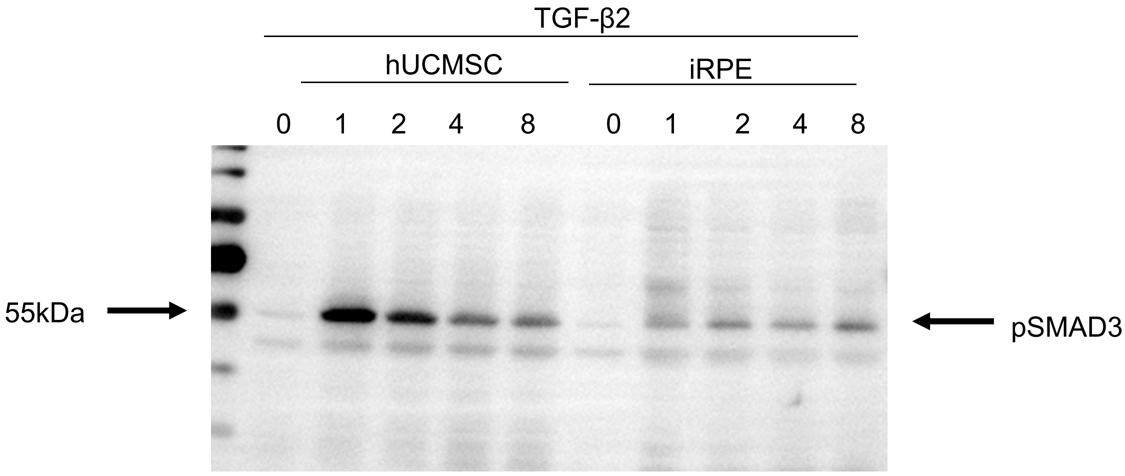
TGF-β2
hUCMSC
iRPE
0 1 2 4 8 0 1 2 4 8
55kDa
pSMAD3

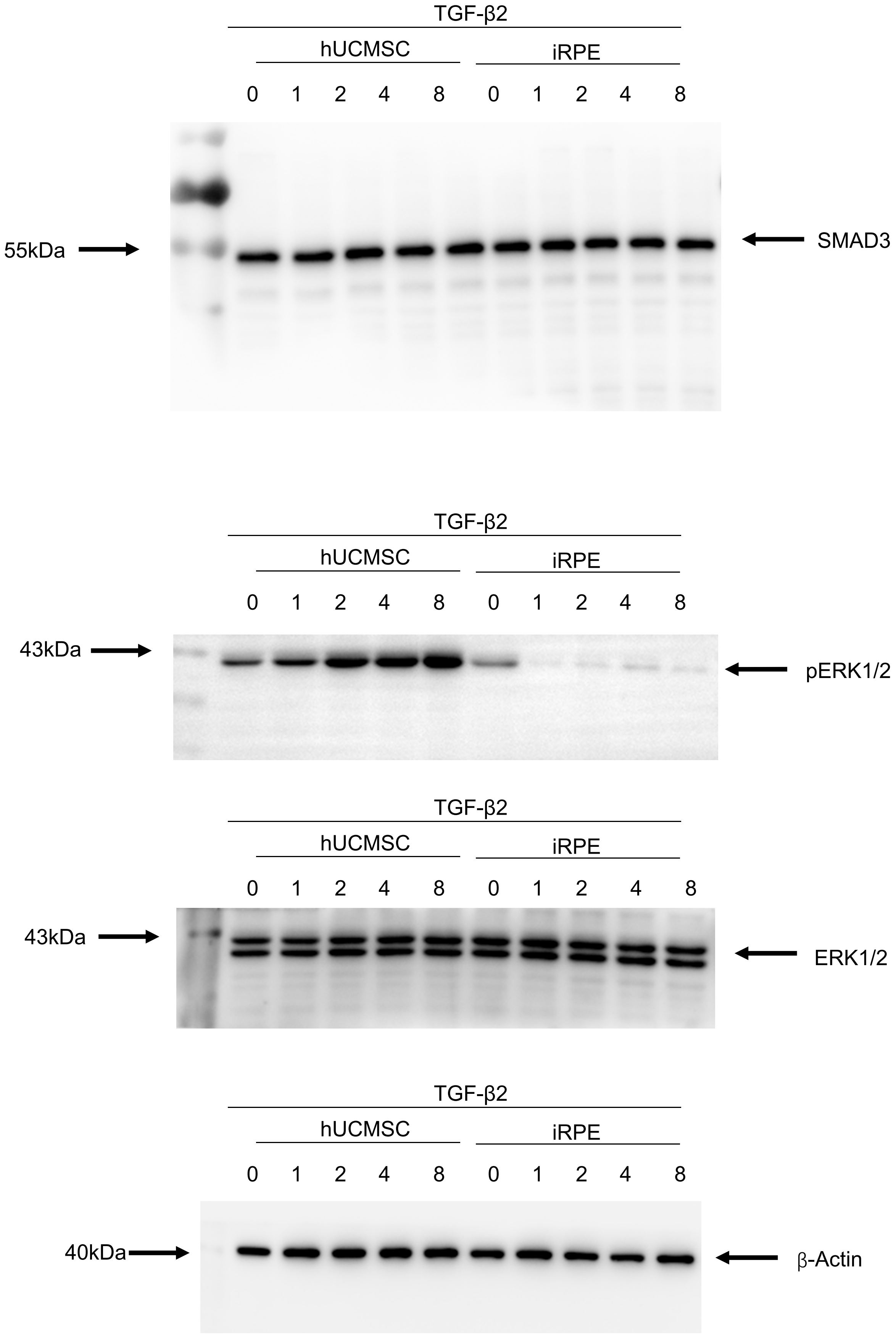
TGF-β2
hUCMSC
iRPE
0 1 2 4 8 0 1 2 4 8
55kDa
SMAD3
TGF-β2
hUCMSC
iRPE
0 1 2 4 8 0 1 2 4 8
43kDa
pERK1/2
TGF-β2
hUCMSC
iRPE
0 1 2 4 8 0 1 2 4 8
43kDa
ERK1/2
TGF-β2
hUCMSC
iRPE
0 1 2 4 8 0 1 2 4 8
40kDa
β-Actin

Figure 4A.

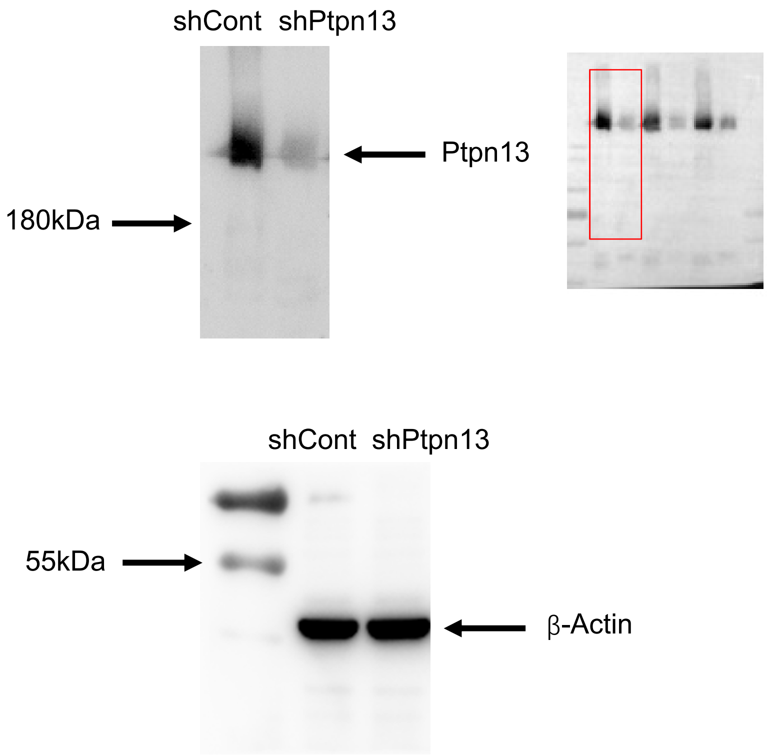

Figure 4E.

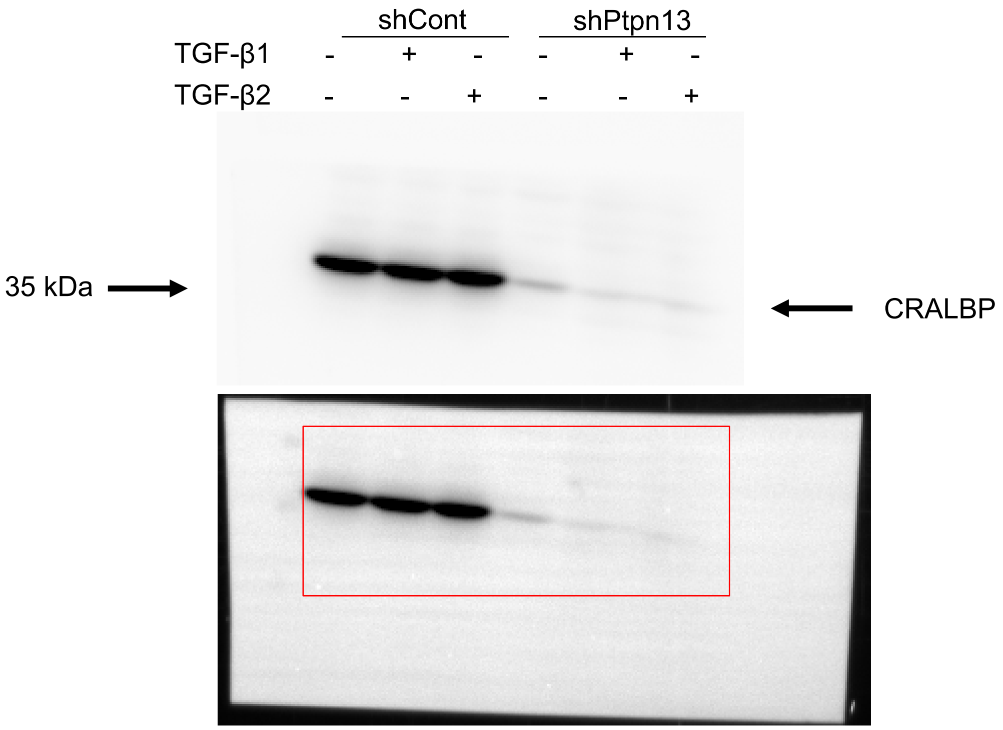

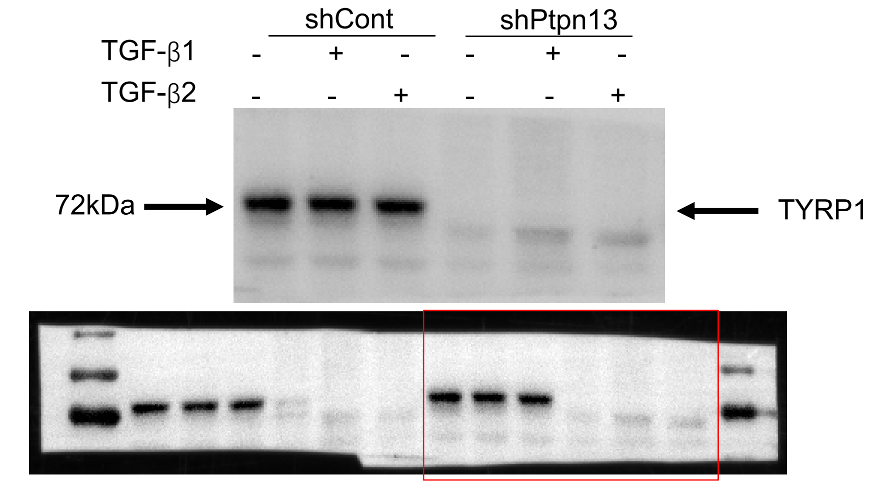
shCont
shPtpn13
TGF-β1
TGF-β2
72kDa
TYRP1

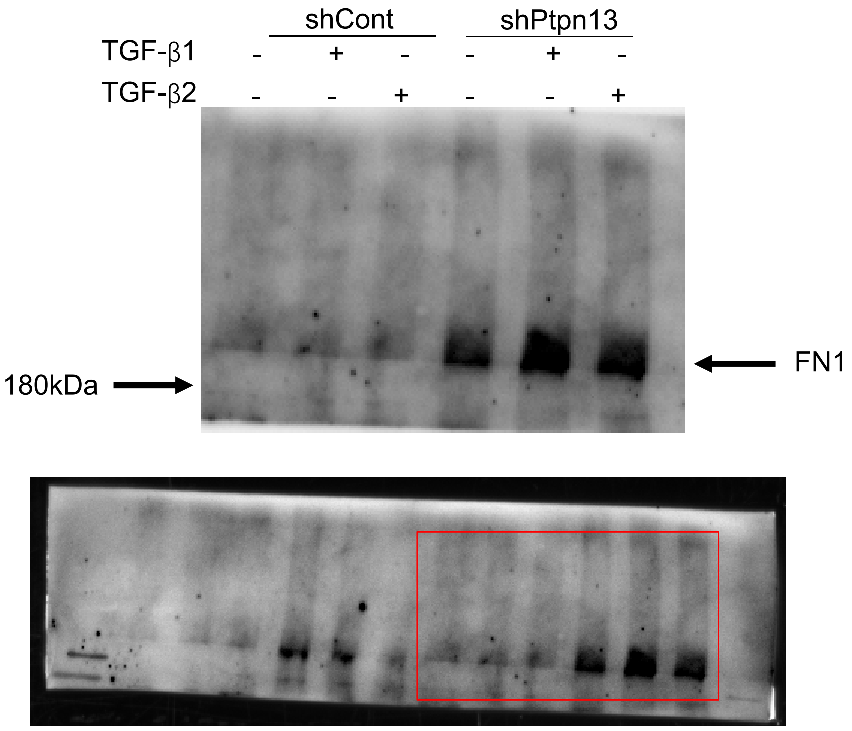
shCont
shPtpn13
TGF-β1
TGF-β2
180kDa
FN1

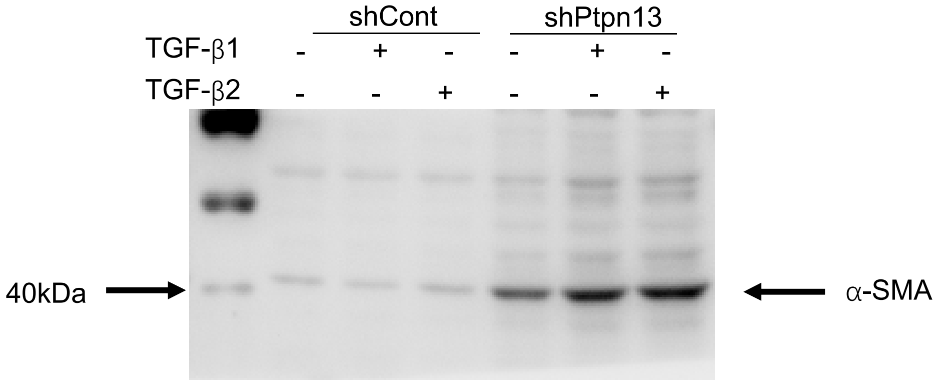
shCont
shPtpn13
TGF-β1
TGF-β2
40kDa
α-SMA

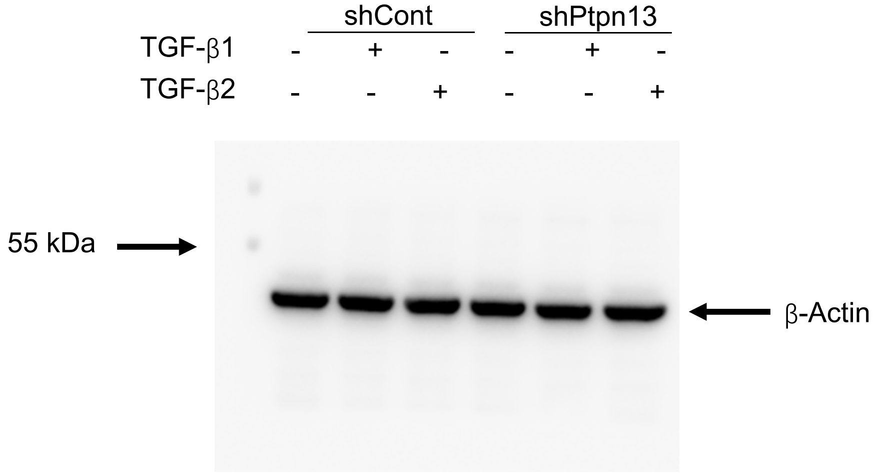
shCont
shPtpn13
TGF-β1
TGF-β2
55 kDa
β-Actin

Figure 4H.

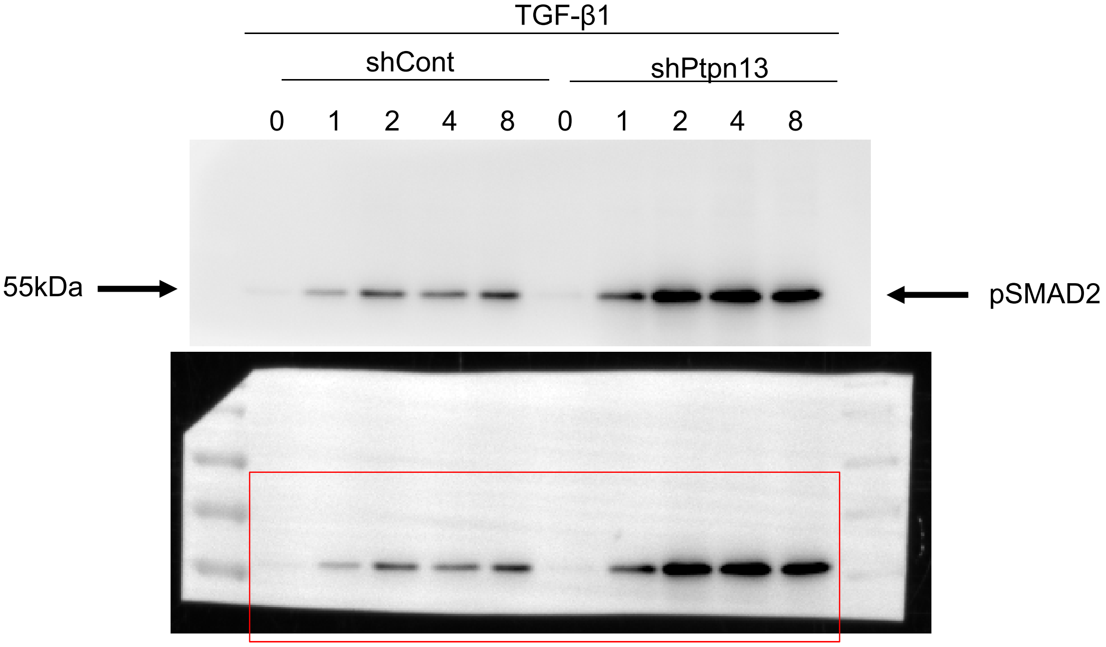
TGF-β1
shCont
shPtpn13
0 1 2 4 8 0 1 2 4 8
55kDa
pSMAD2

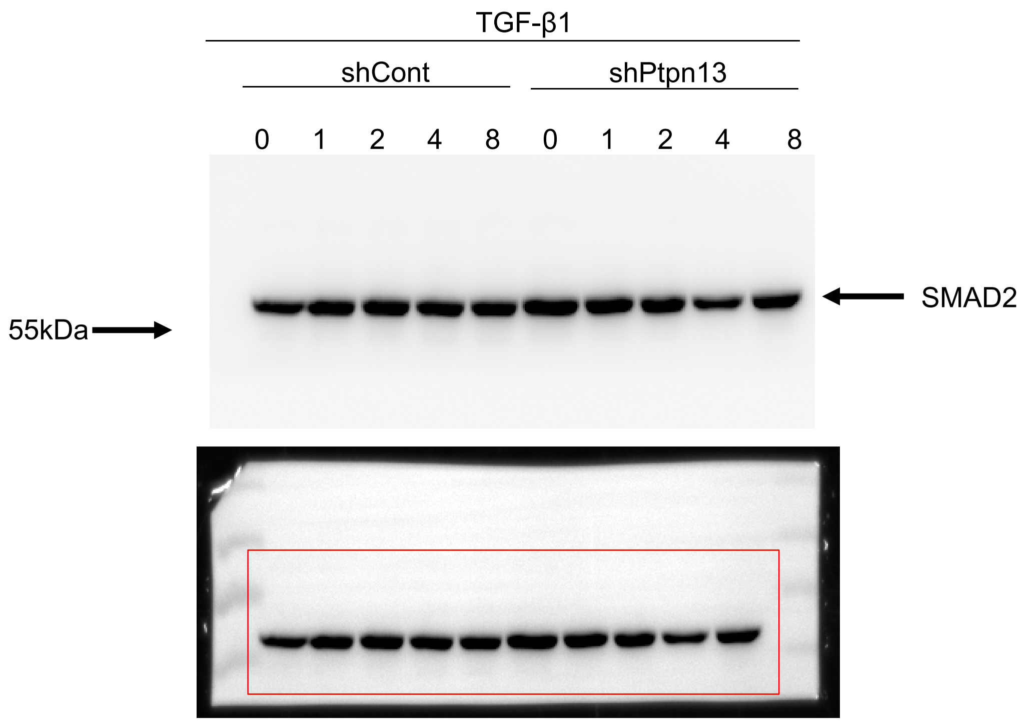
TGF-β1
shCont
shPtpn13
0 1 2 4 8 0 1 2 4 8
55kDa
SMAD2

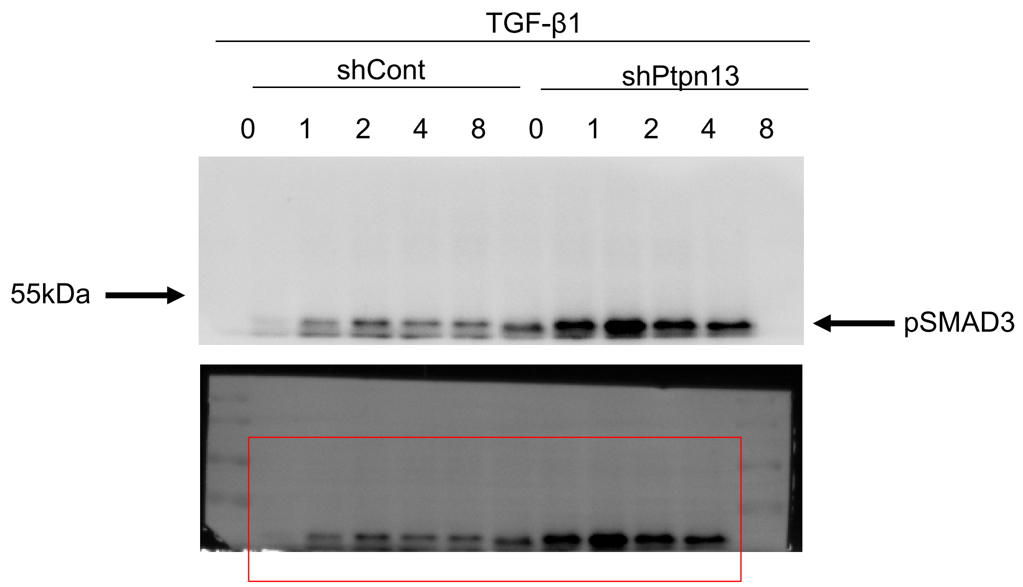
TGF-β1
shCont
shPtpn13
0 1 2 4 8 0 1 2 4 8
55kDa
pSMAD3

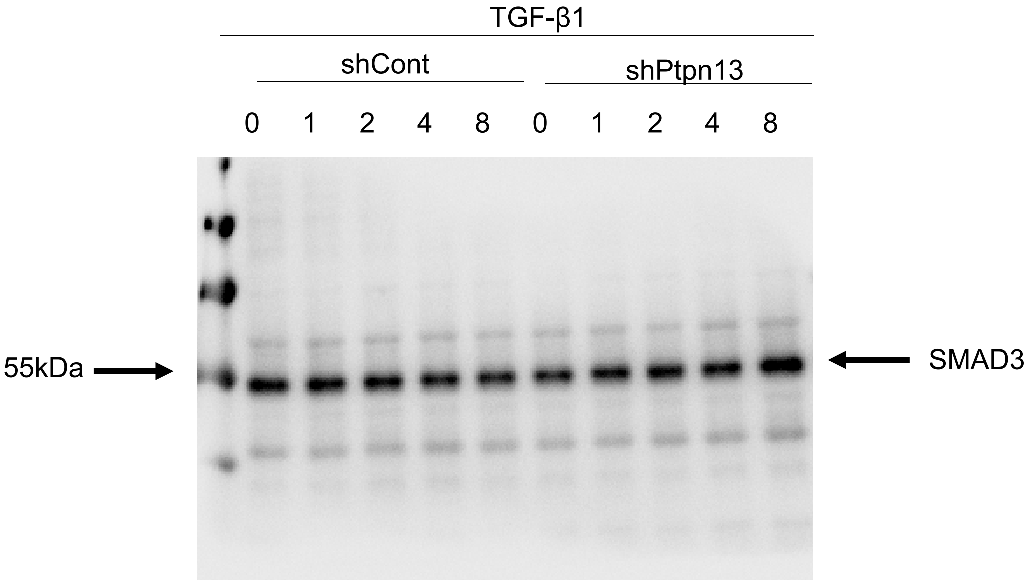
TGF-β1
shCont
shPtpn13
0 1 2 4 8 0 1 2 4 8
55kDa
SMAD3

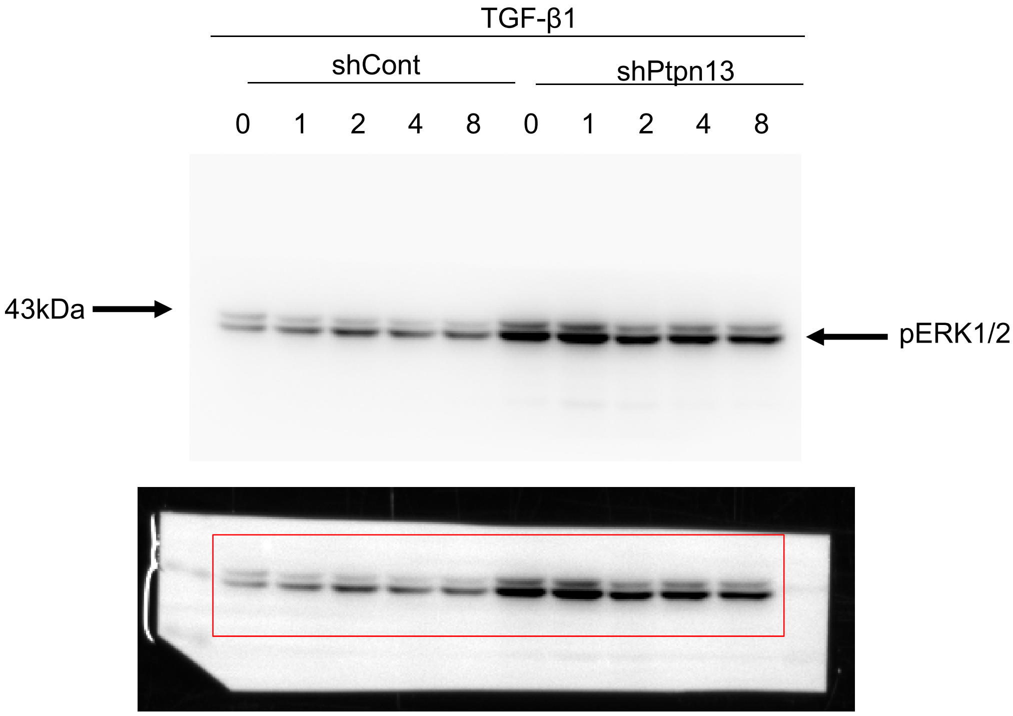
TGF-β1
shCont
shPtpn13
0 1 2 4 8 0 1 2 4 8
43kDa
pERK1/2

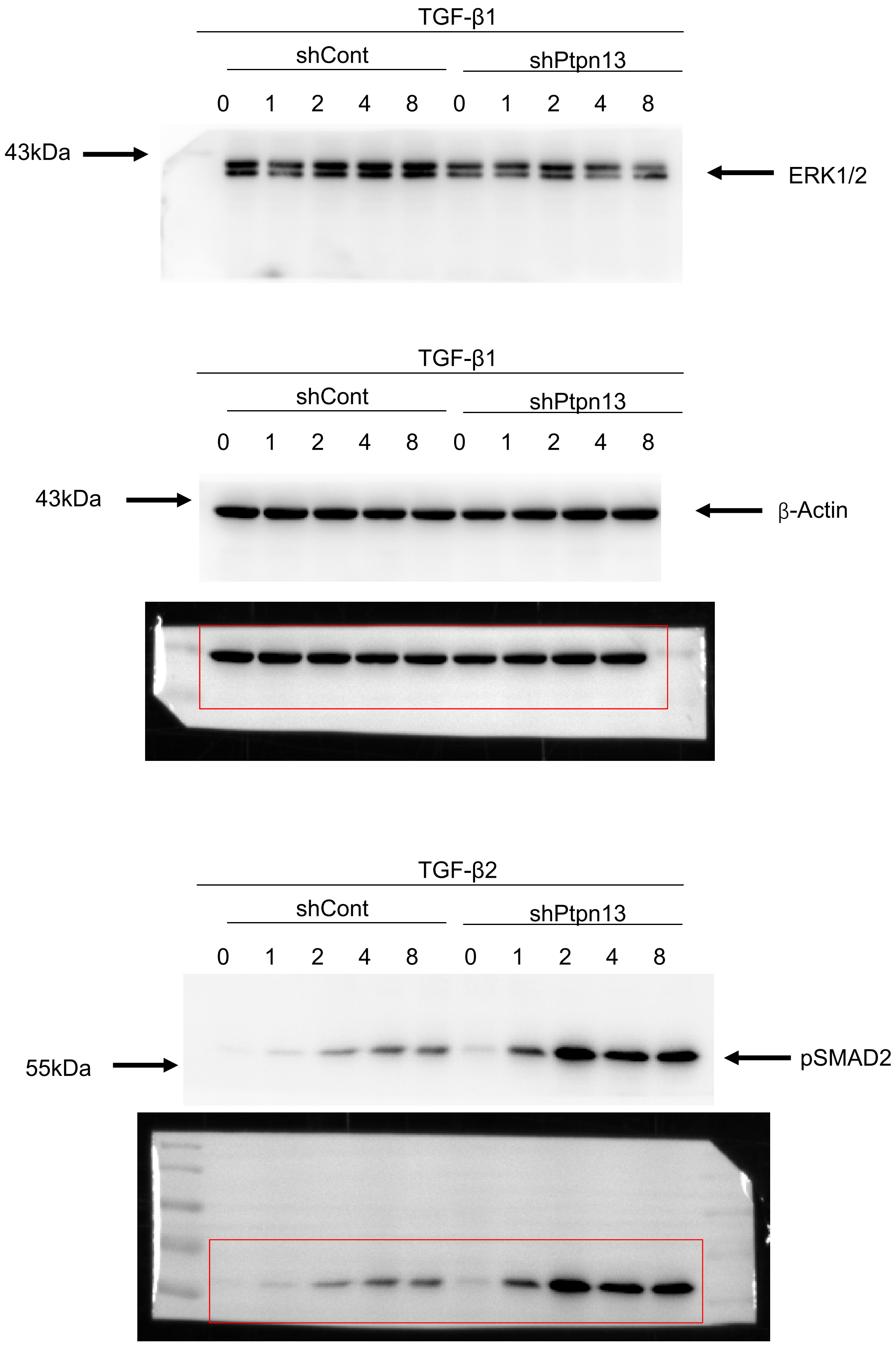

TGF-β1
shCont
shPtpn13
0 1 2 4 8 0 1 2 4 8
43kDa
ERK1/2
TGF-β1
shCont
shPtpn13
0 1 2 4 8 0 1 2 4 8
43kDa
β-Actin
TGF-β2
shCont
shPtpn13
0 1 2 4 8 0 1 2 4 8
55kDa
pSMAD2

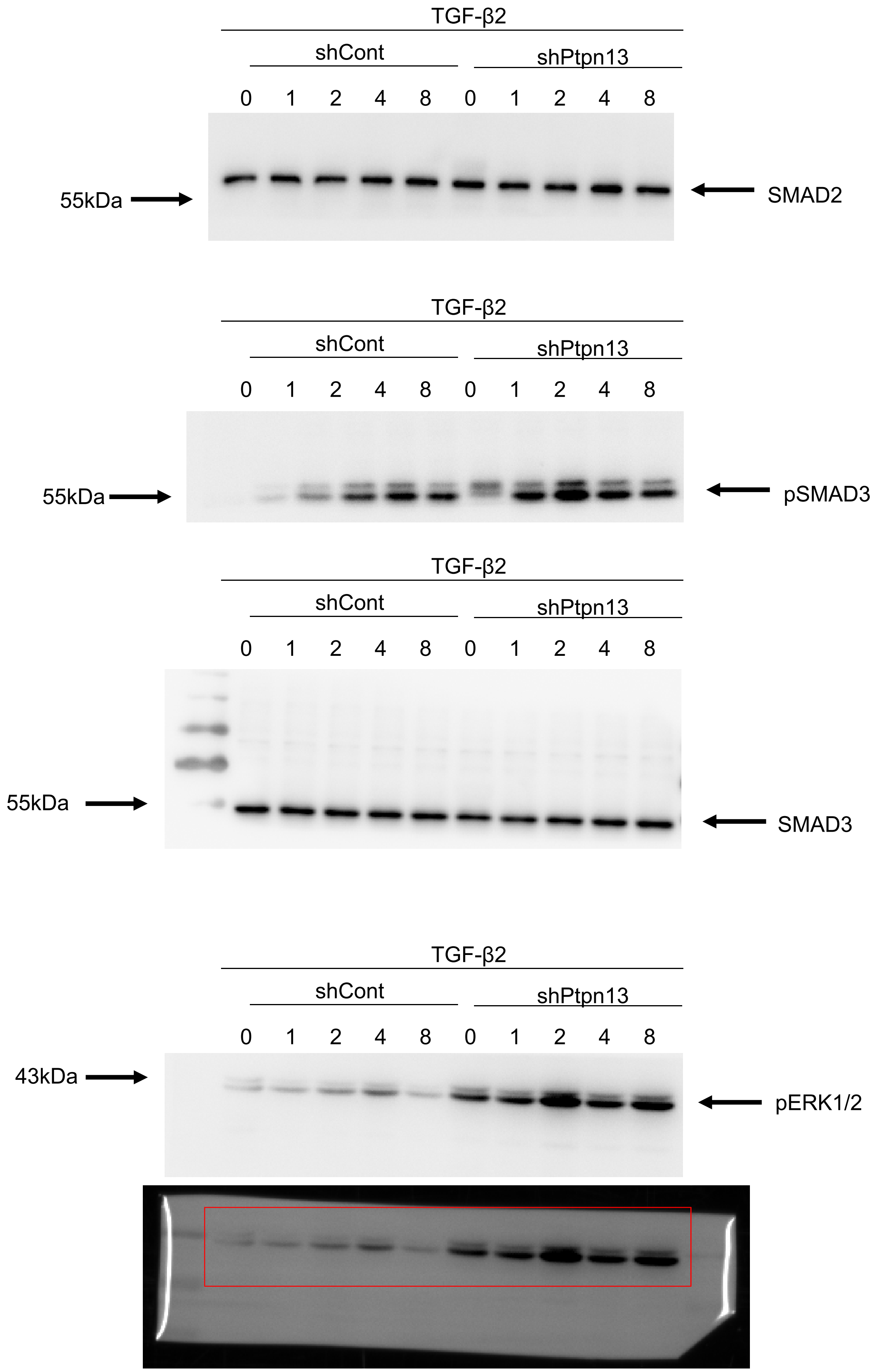
TGF-β2
shCont
shPtpn13
0 1 2 4 8 0 1 2 4 8
55kDa
SMAD2
TGF-β2
shCont
shPtpn13
0 1 2 4 8 0 1 2 4 8
55kDa
pSMAD3
TGF-β2
shCont
shPtpn13
0 1 2 4 8 0 1 2 4 8
55kDa
SMAD3
TGF-β2
shCont
shPtpn13
0 1 2 4 8 0 1 2 4 8
43kDa
pERK1/2

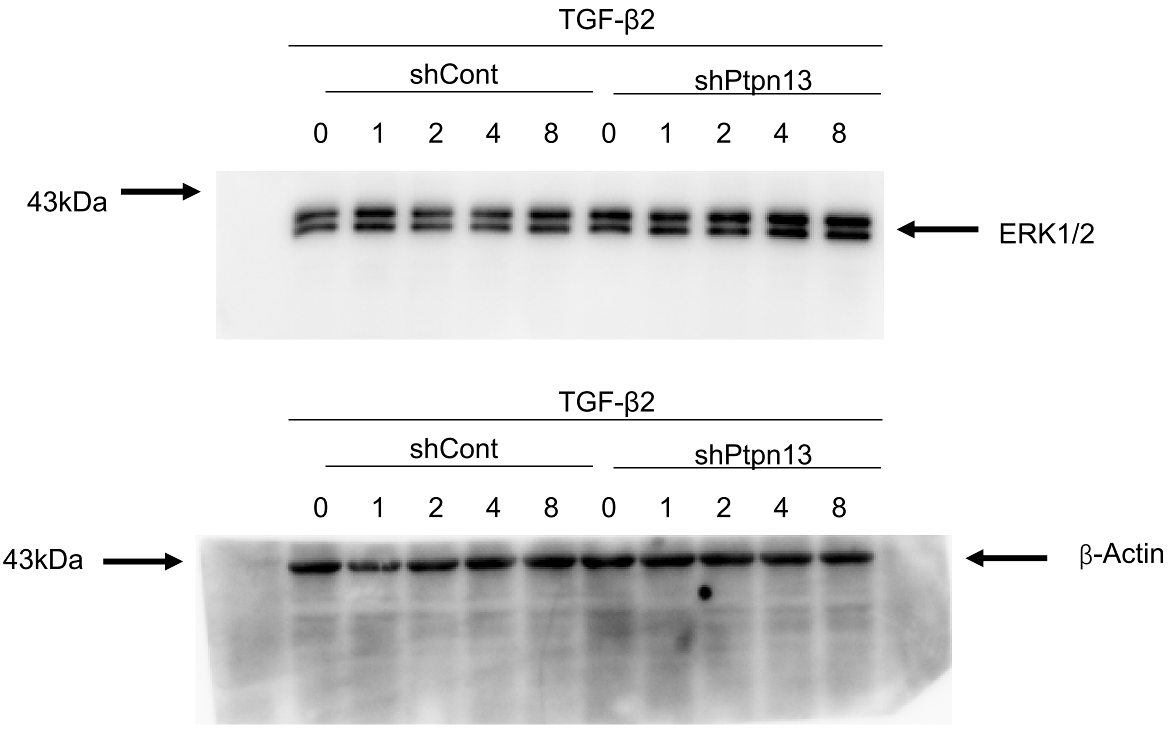
TGF-β2
shCont
shPtpn13
0 1 2 4 8 0 1 2 4 8
43kDa
ERK1/2
TGF-β2
shCont
shPtpn13
0 1 2 4 8 0 1 2 4 8
43kDa
β-Actin

Figure 5B.

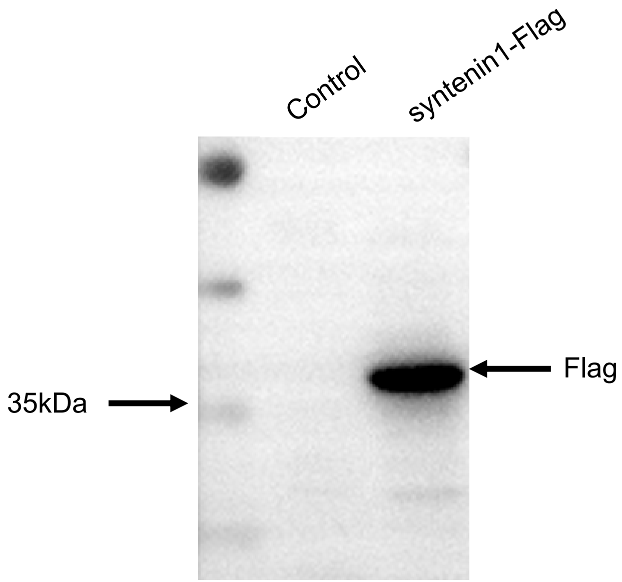
Control
syntenin1-Flag
35kDa
Flag

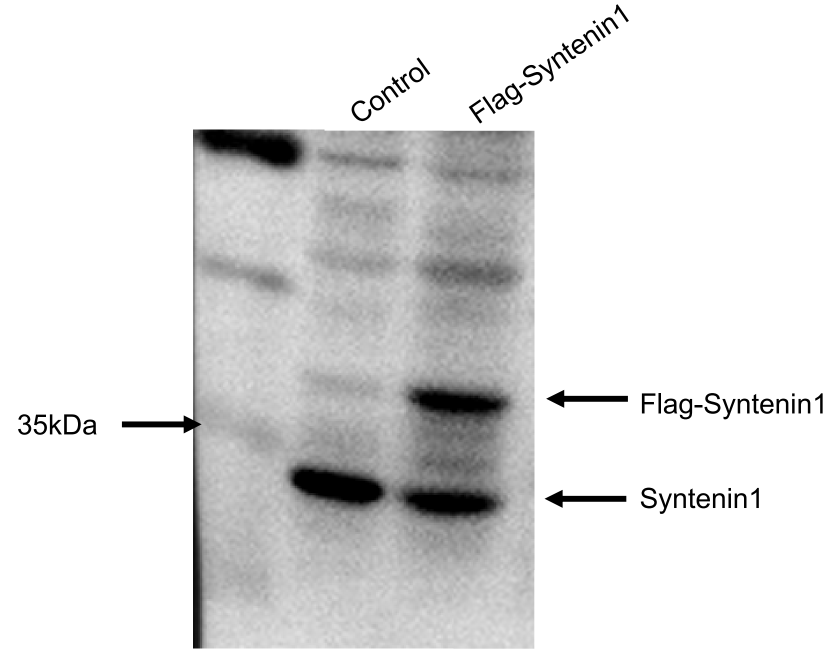
Control
Flag-Syntenin1
35kDa
Flag-Syntenin1
Syntenin1

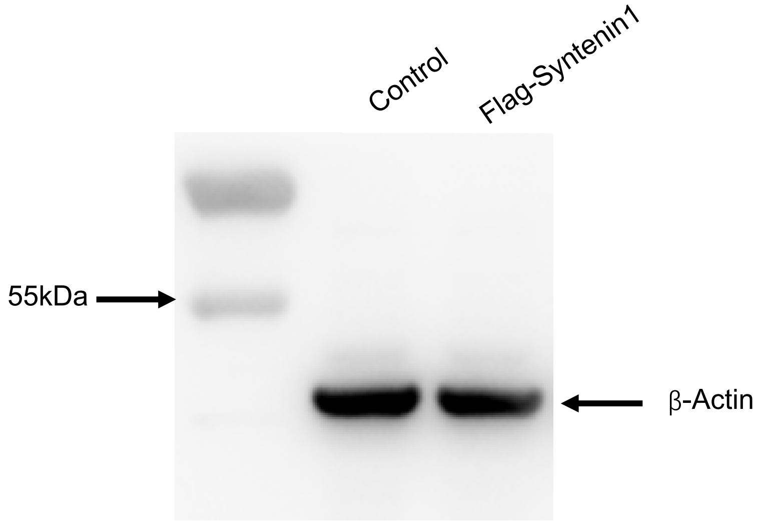
Control
Flag-Syntenin1
55kDa
β-Actin

Figure 5C.

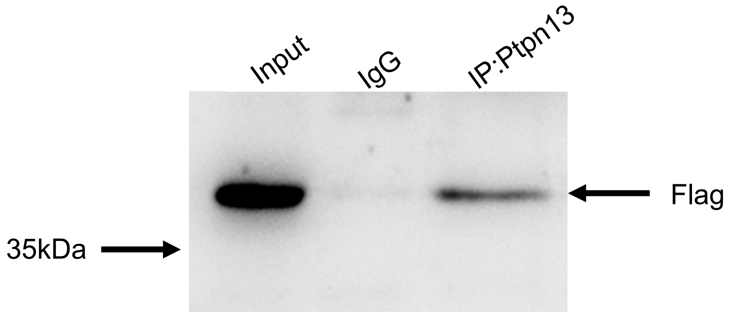
Input
IgG
IP:Ptpn13
Flag
35kDa

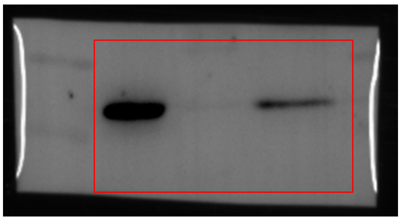

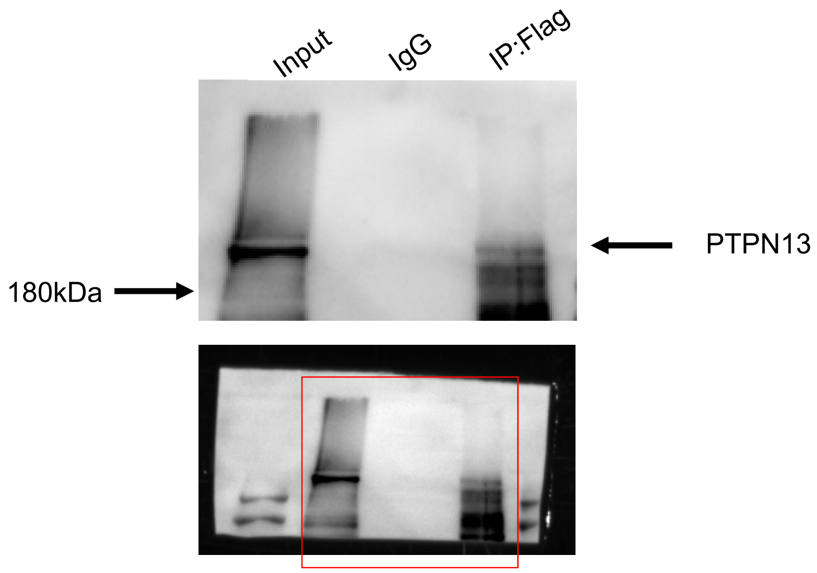
Input
IgG
IP:Flag
PTPN13
180kDa

Figure 5D.

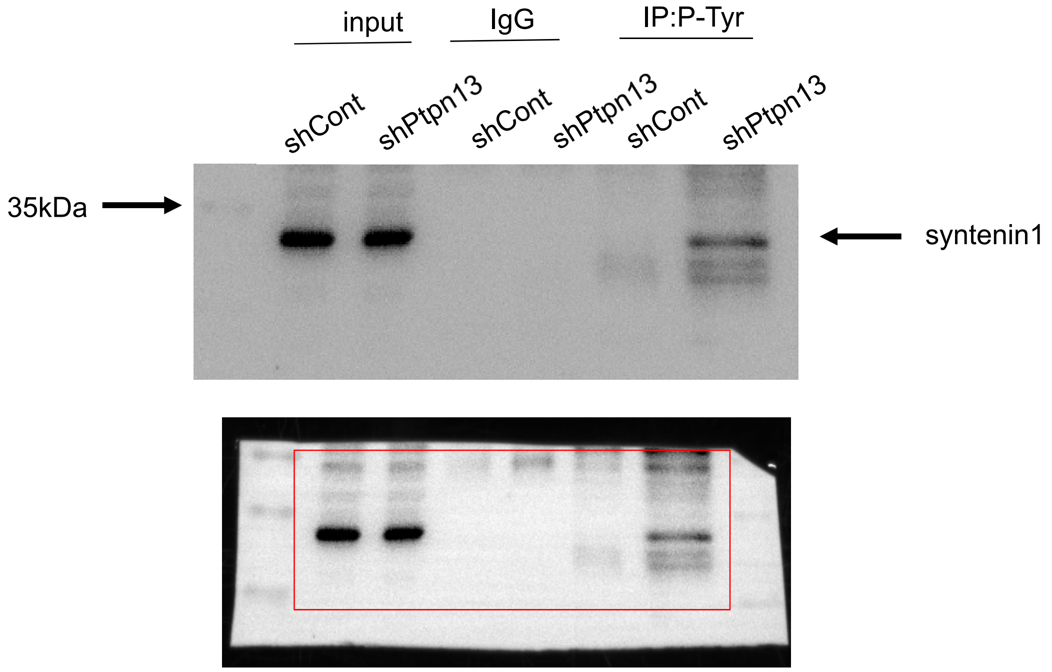
input
IgG
IP:P-Tyr
shCont
shPtpn13
shCont
shPtpn13
shCont
shPtpn13
35kDa
syntenin1

Figure 5F.

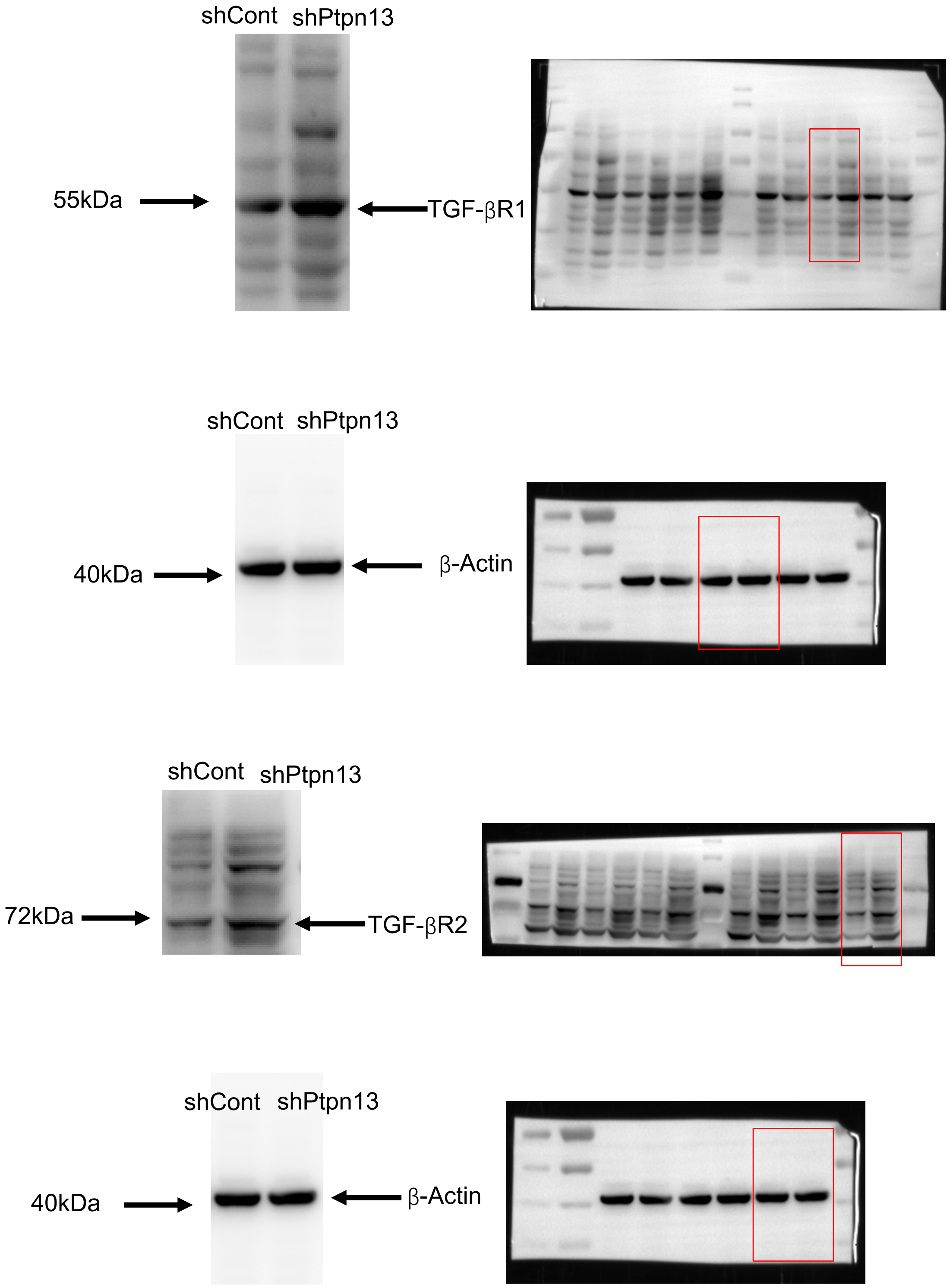
shCont
shPtpn13
55kDa
TGF-βR1
shCont
shPtpn13
40kDa
β-Actin
shCont
shPtpn13
72kDa
TGF-βR2
shCont
shPtpn13
40kDa
β-Actin

Figure 6B.

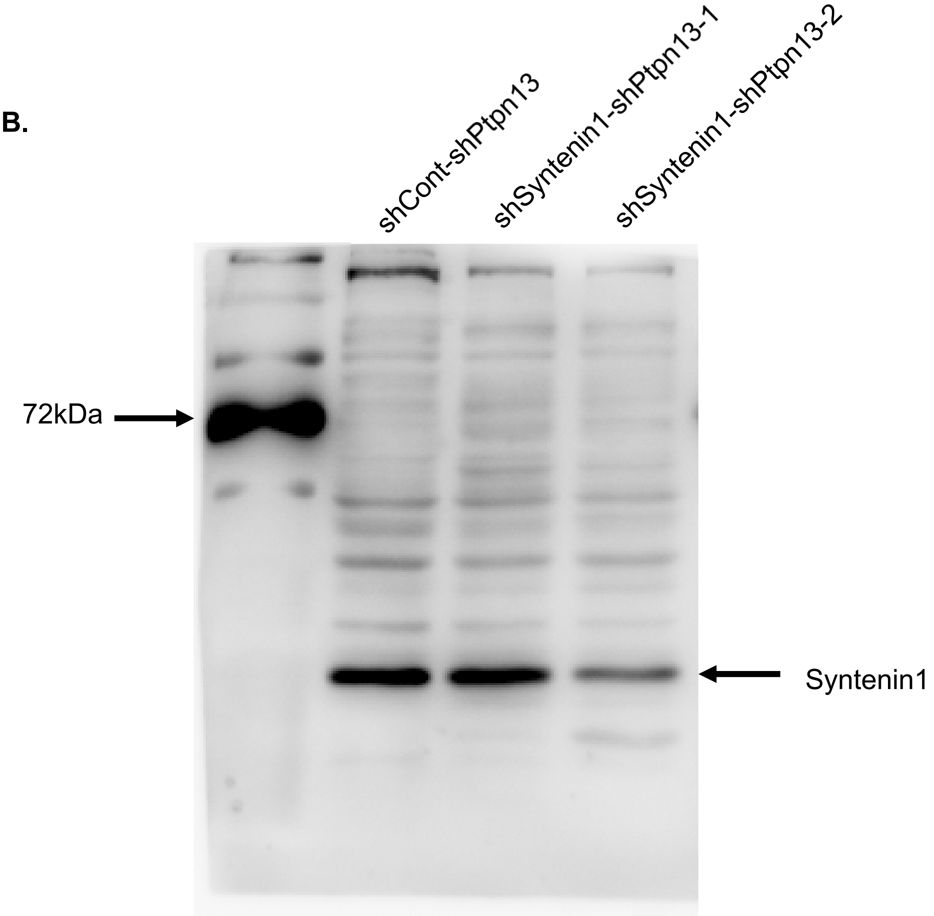
shCont-shPtpn13
shSyntenin1-shPtpn13-1
shSyntenin1-shPtpn13-2
72kDa
Syntenin1

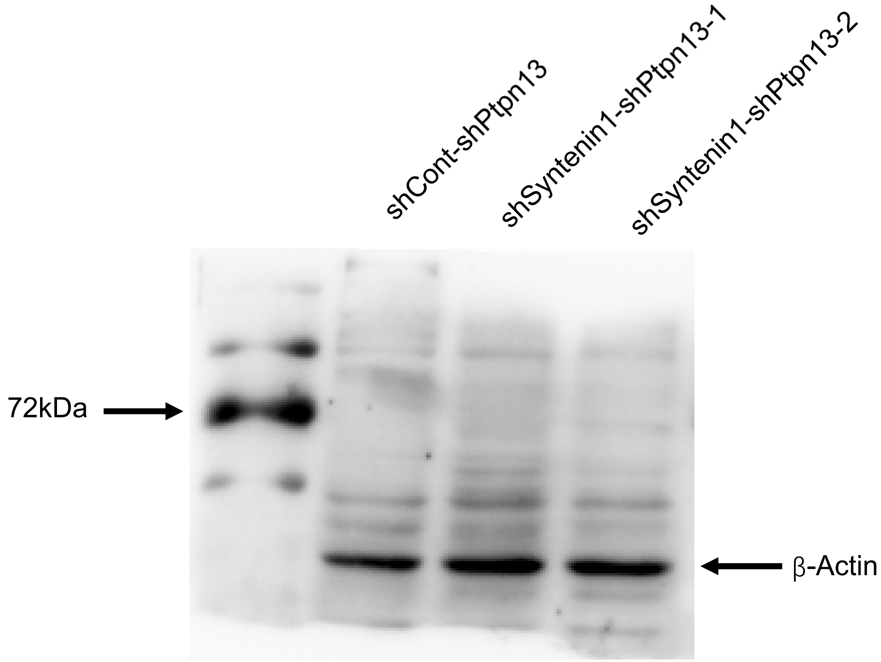
shCont-shPtpn13
shSyntenin1-shPtpn13-1
shSyntenin1-shPtpn13-2
72kDa
β-Actin

Figure 6D.

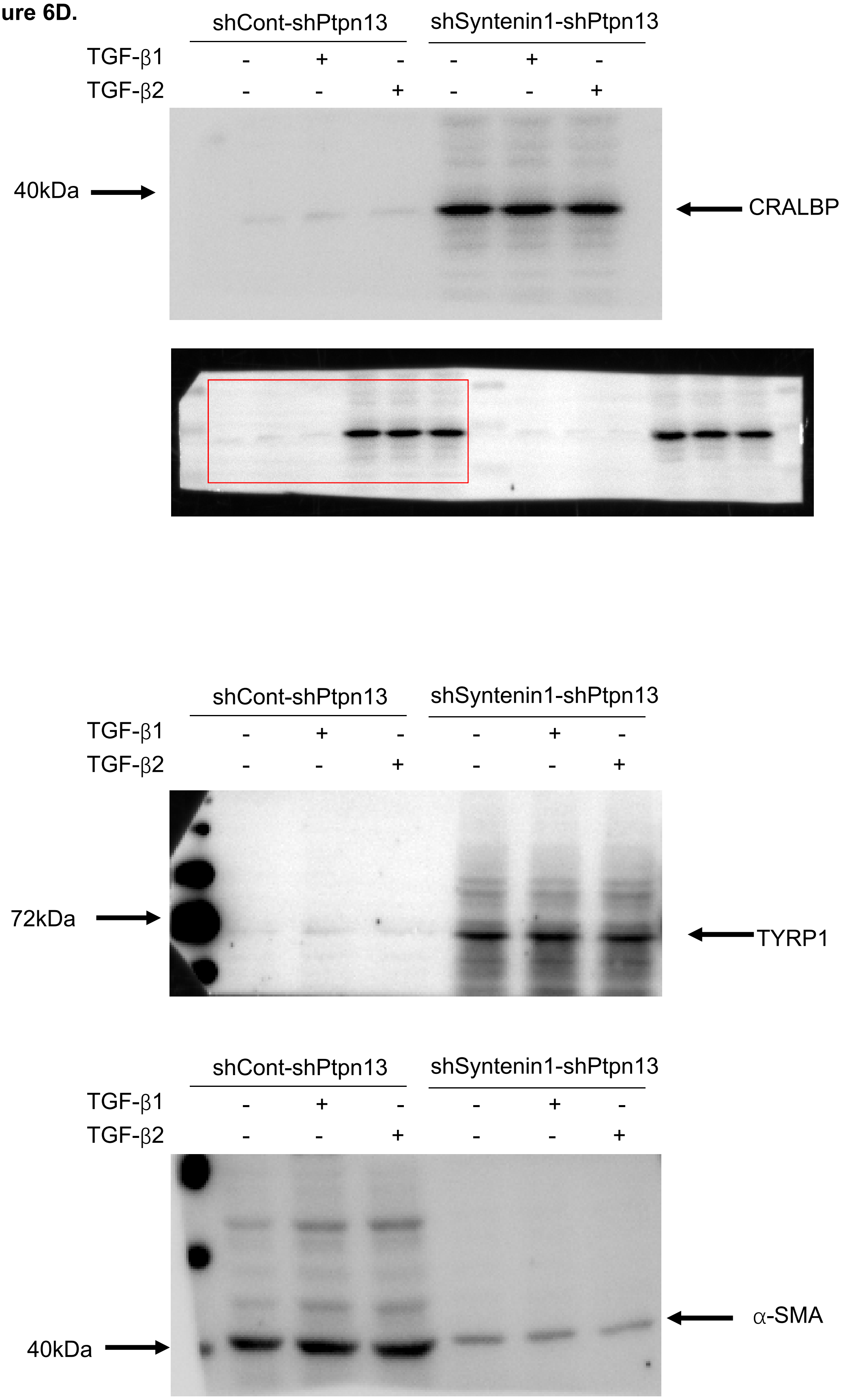
shCont-shPtpn13
shSyntenin1-shPtpn13
TGF-β1 - + - - + -
TGF-β2 - - + - - +
40kDa
CRALBP
shCont-shPtpn13
shSyntenin1-shPtpn13
TGF-β1 - + - - + -
TGF-β2 - - + - - +
72kDa
TYRP1
shCont-shPtpn13
shSyntenin1-shPtpn13
TGF-β1 - + - - + -
TGF-β2 - - + - - +
α-SMA
40kDa

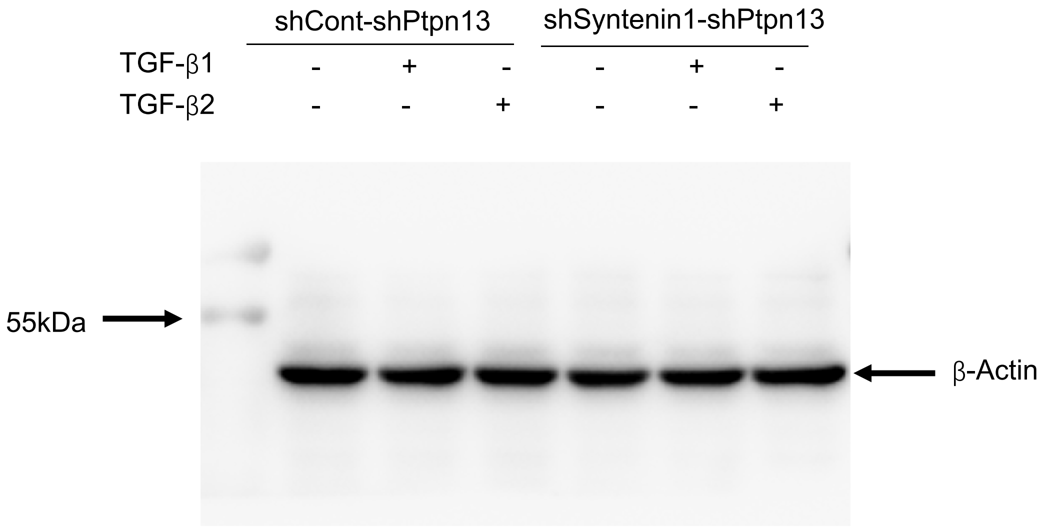
shCont-shPtpn13
shSyntenin1-shPtpn13
TGF-β1 - + - - + -
TGF-β2 - - + - - +
55kDa
β-Actin

Figure 6F.

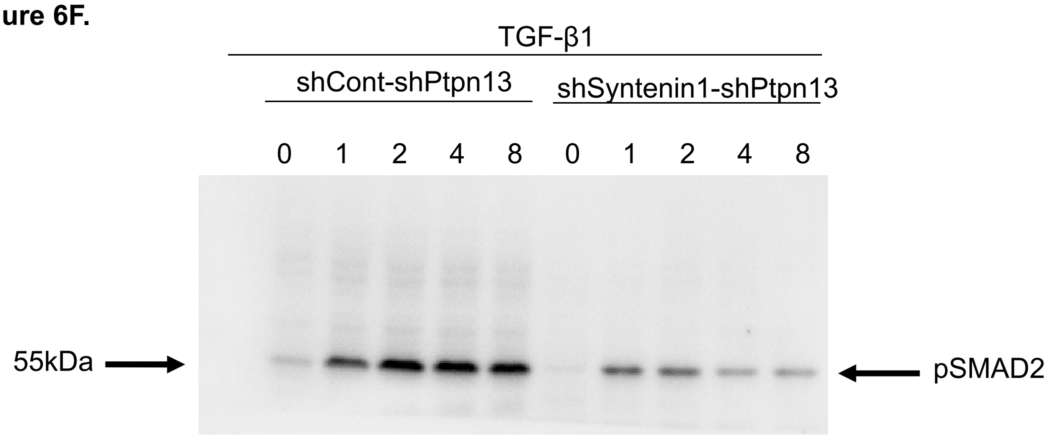
TGF-β1
shCont-shPtpn13
shSyntenin1-shPtpn13
0 1 2 4 8 0 1 2 4 8
55kDa
pSMAD2

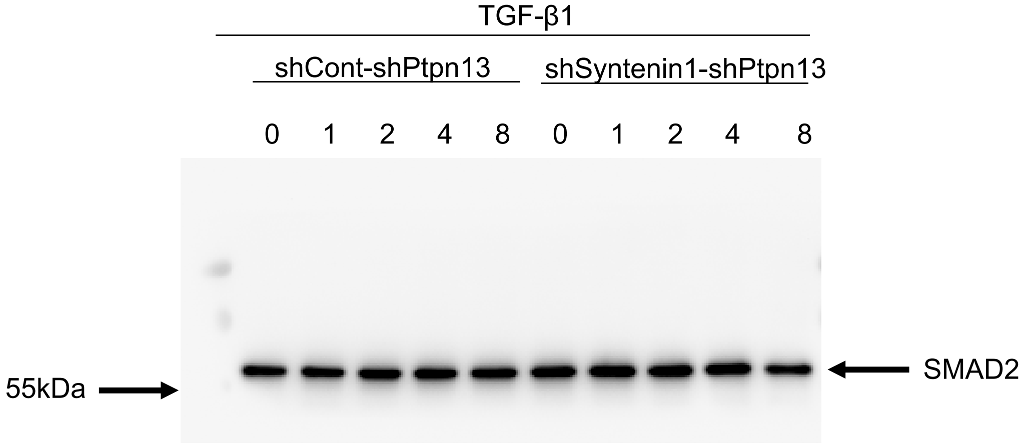
TGF-β1
shCont-shPtpn13
shSyntenin1-shPtpn13
0 1 2 4 8 0 1 2 4 8
55kDa
SMAD2

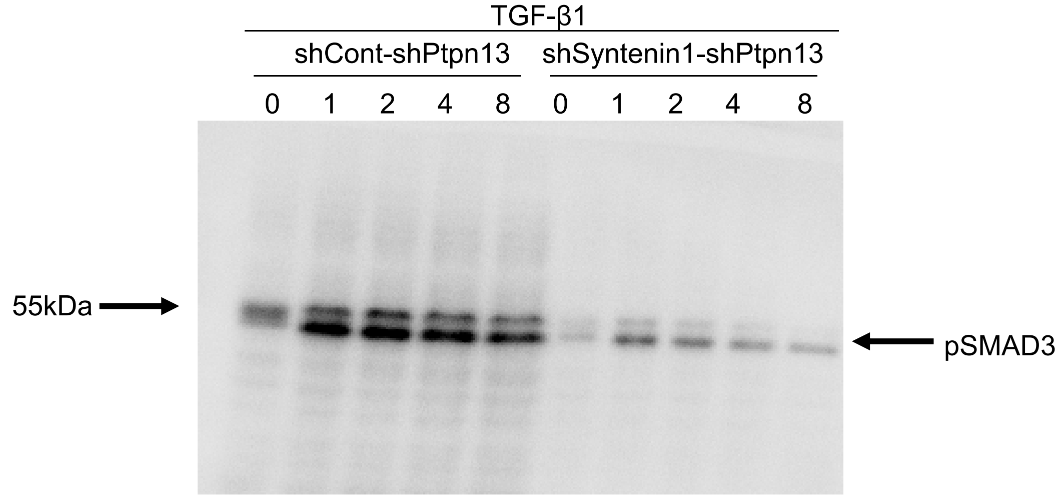
TGF-β1
shCont-shPtpn13
shSyntenin1-shPtpn13
0 1 2 4 8 0 1 2 4 8
55kDa
pSMAD3

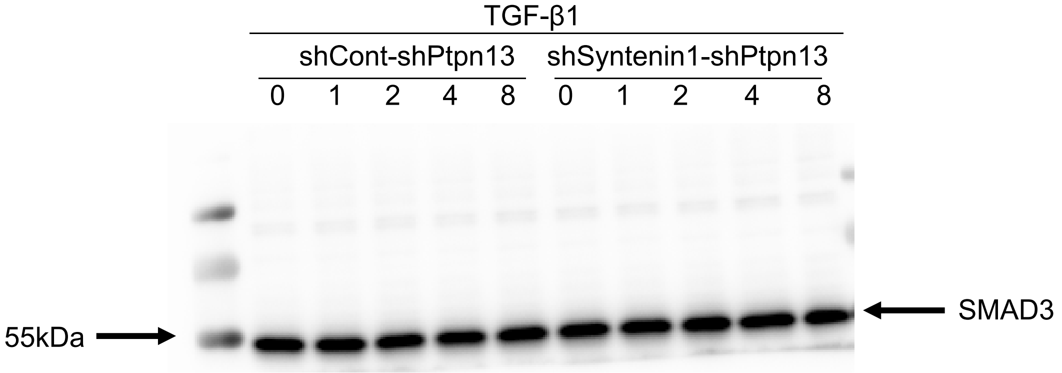
TGF-β1
shCont-shPtpn13
shSyntenin1-shPtpn13
0 1 2 4 8 0 1 2 4 8
55kDa
SMAD3

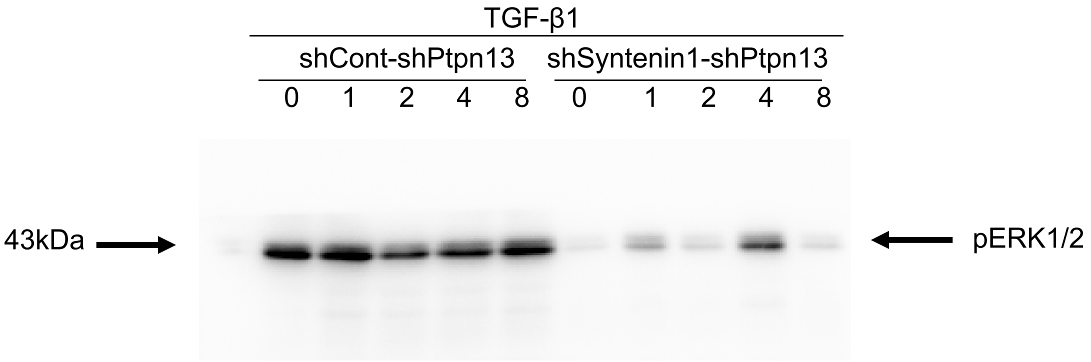
TGF-β1
shCont-shPtpn13
shSyntenin1-shPtpn13
0 1 2 4 8 0 1 2 4 8
43kDa
pERK1/2

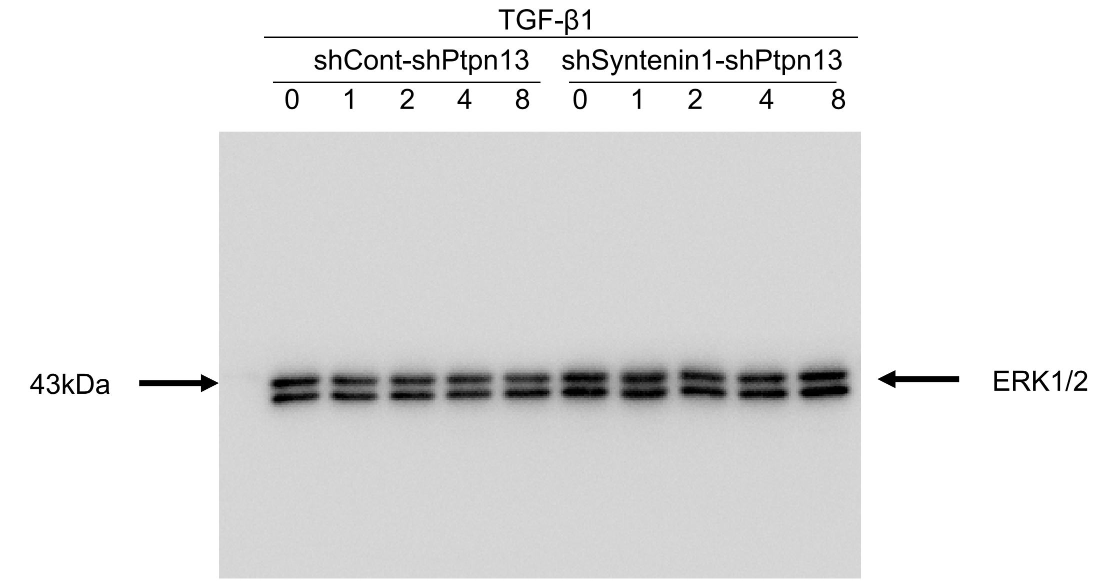
TGF-β1
shCont-shPtpn13
shSyntenin1-shPtpn13
0 1 2 4 8 0 1 2 4 8
43kDa
ERK1/2

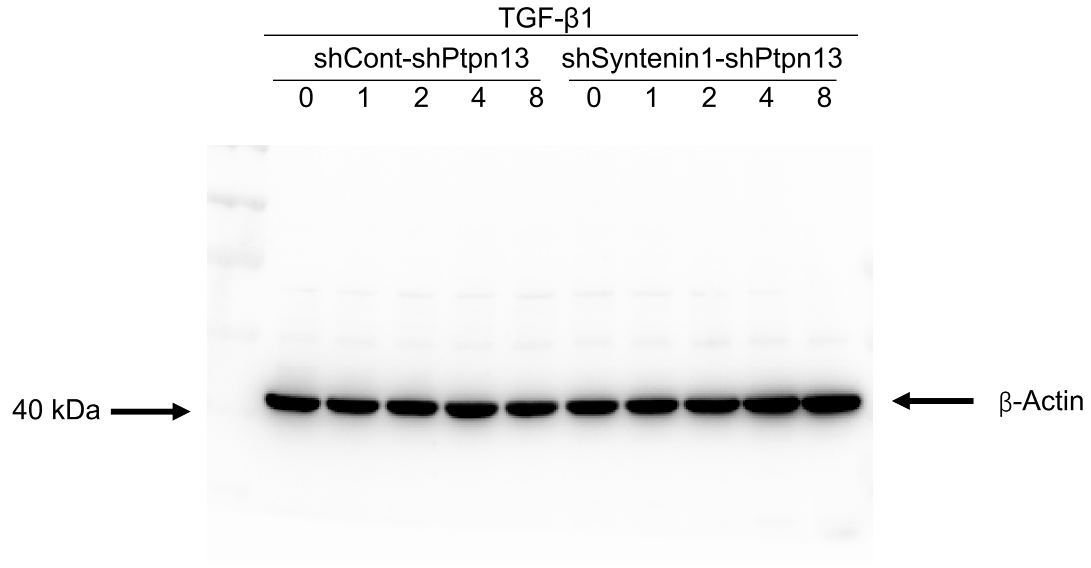
TGF-β1
shCont-shPtpn13
shSyntenin1-shPtpn13
0 1 2 4 8 0 1 2 4 8
40 kDa
β-Actin

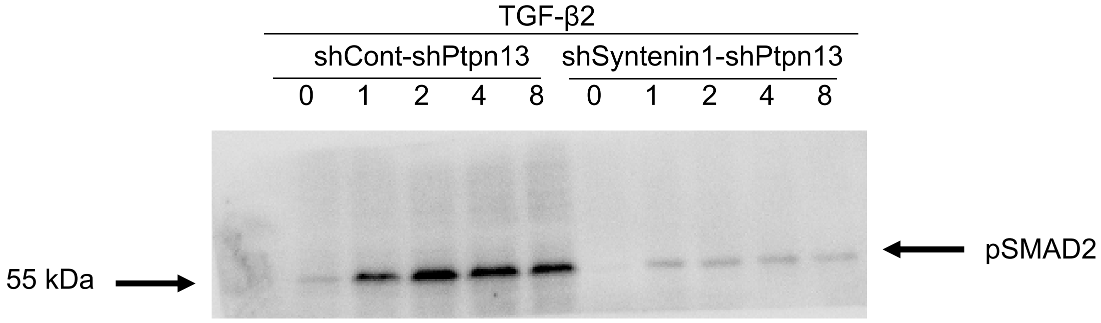
TGF-β2
shCont-shPtpn13
shSyntenin1-shPtpn13
0 1 2 4 8 0 1 2 4 8
55 kDa
pSMAD2

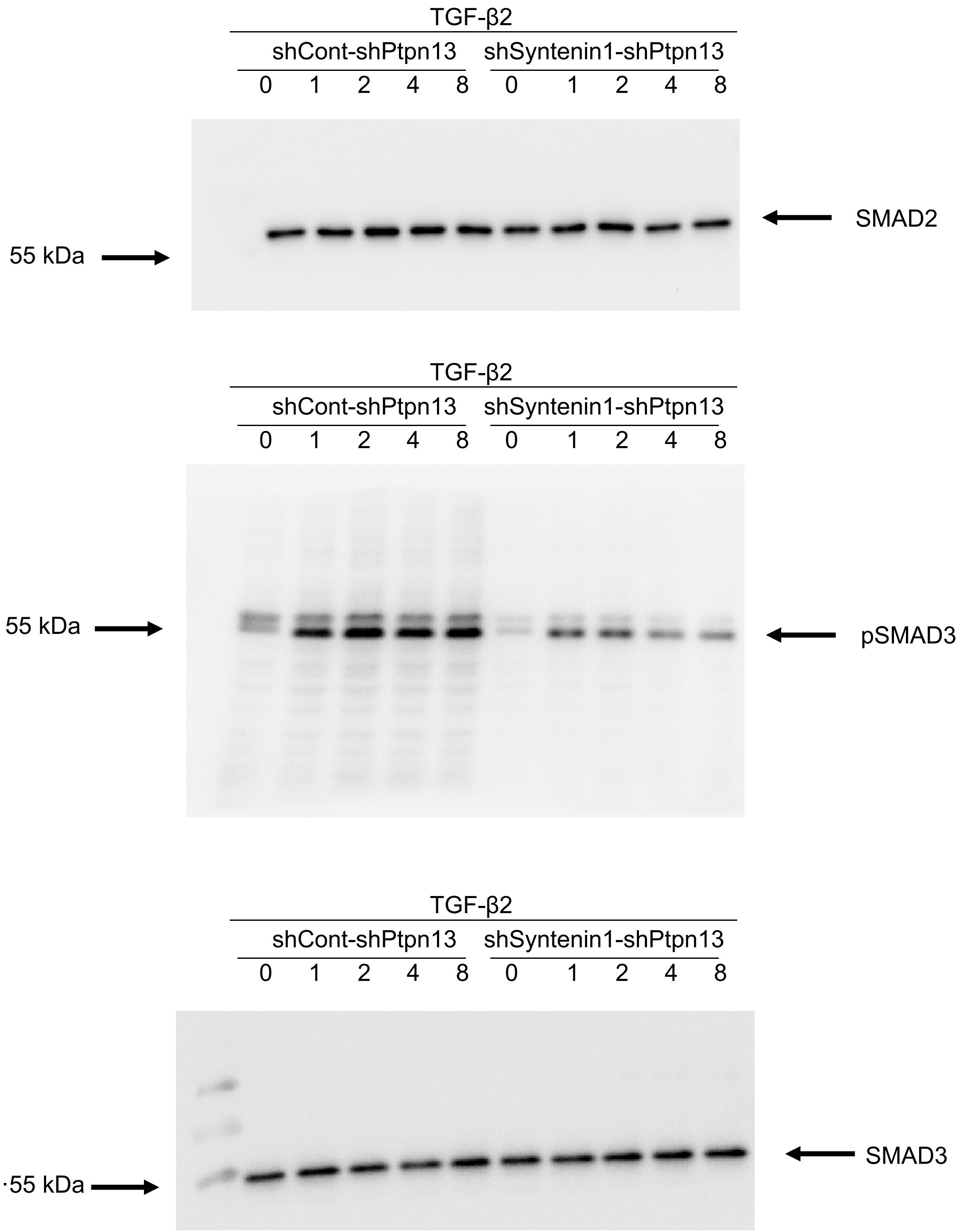
TGF-β2
shCont-shPtpn13
shSyntenin1-shPtpn13
0 1 2 4 8 0 1 2 4 8
55 kDa
SMAD2
TGF-β2
shCont-shPtpn13
shSyntenin1-shPtpn13
0 1 2 4 8 0 1 2 4 8
55 kDa
pSMAD3
TGF-β2
shCont-shPtpn13
shSyntenin1-shPtpn13
0 1 2 4 8 0 1 2 4 8
55 kDa
SMAD3

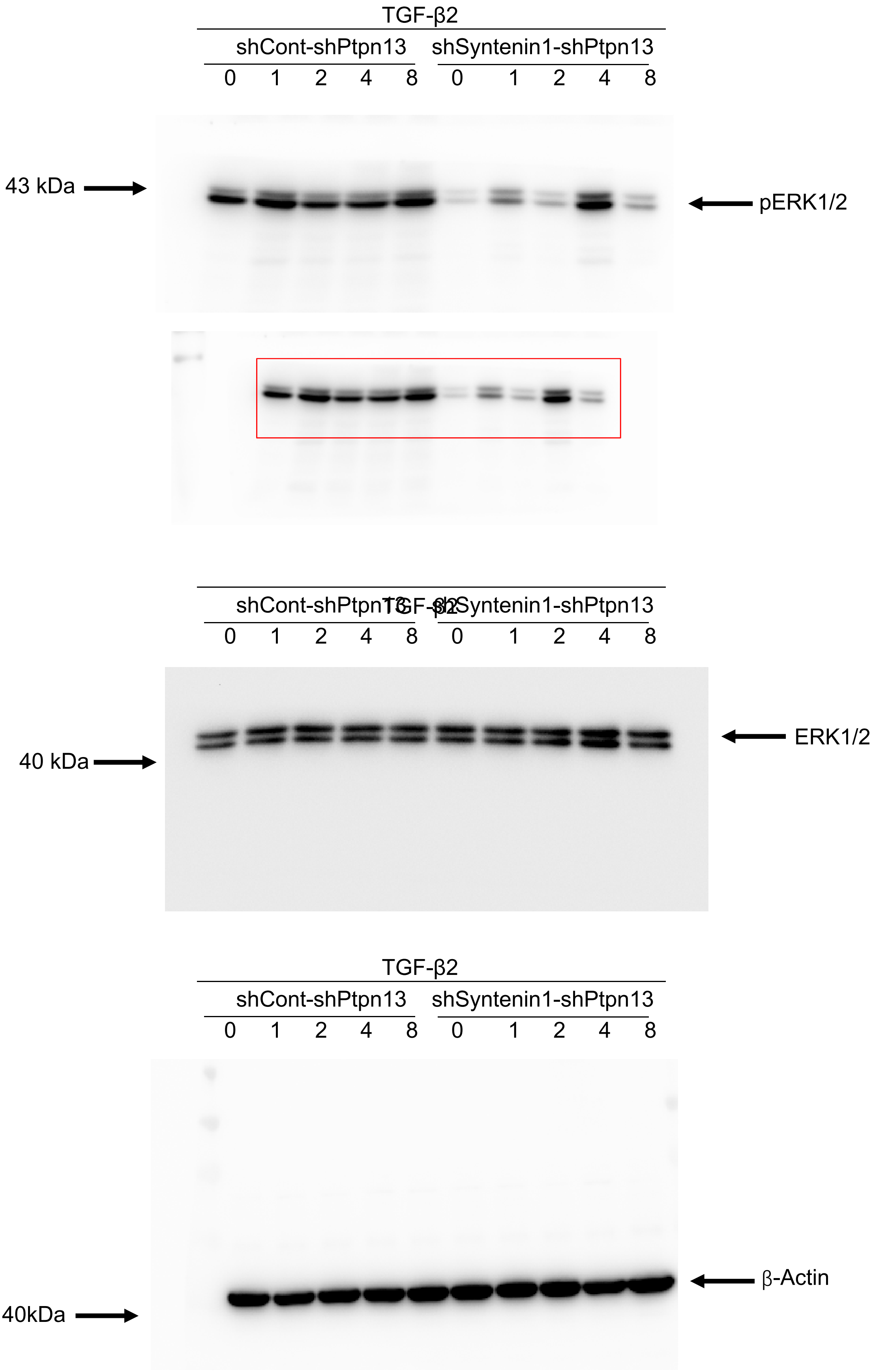
TGF-β2
shCont-shPtpn13
shSyntenin1-shPtpn13
0 1 2 4 8 0 1 2 4 8
43 kDa
pERK1/2
TGF-β2
shCont-shPtpn13
shSyntenin1-shPtpn13
0 1 2 4 8 0 1 2 4 8
40 kDa
ERK1/2
TGF-β2
shCont-shPtpn13
shSyntenin1-shPtpn13
0 1 2 4 8 0 1 2 4 8
40kDa
β-Actin

Supplementary figure 6B

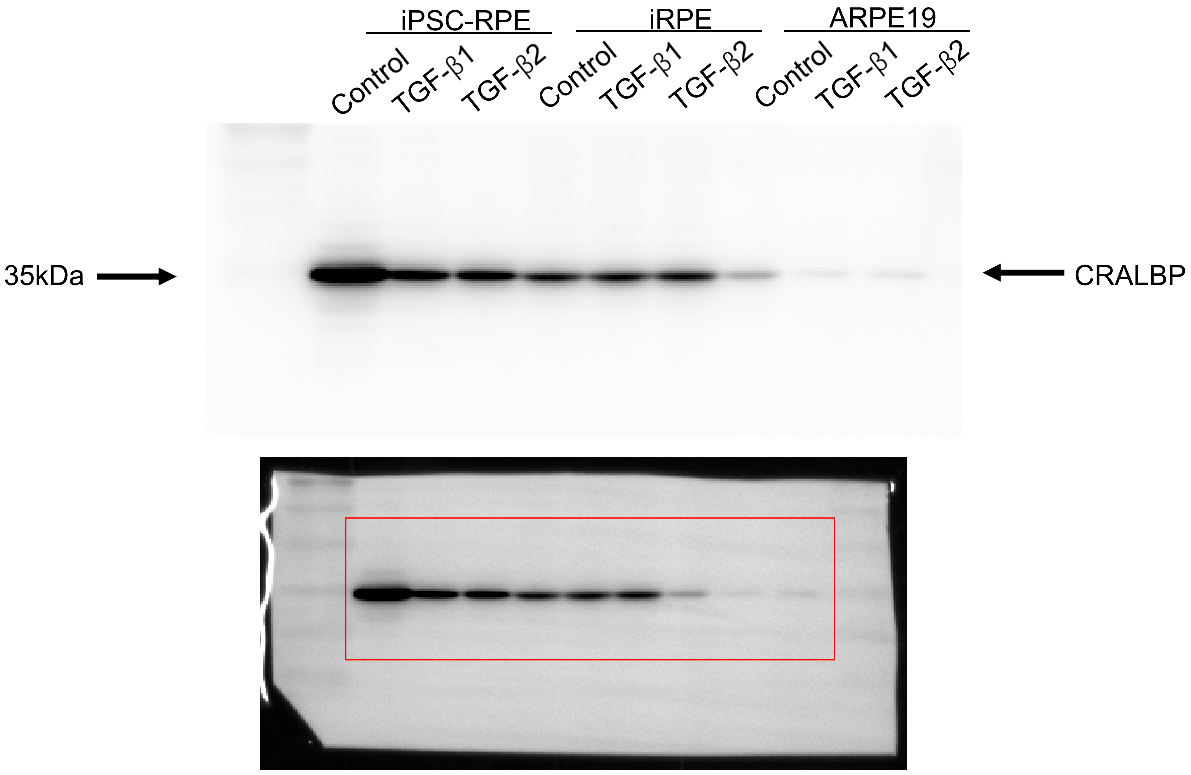

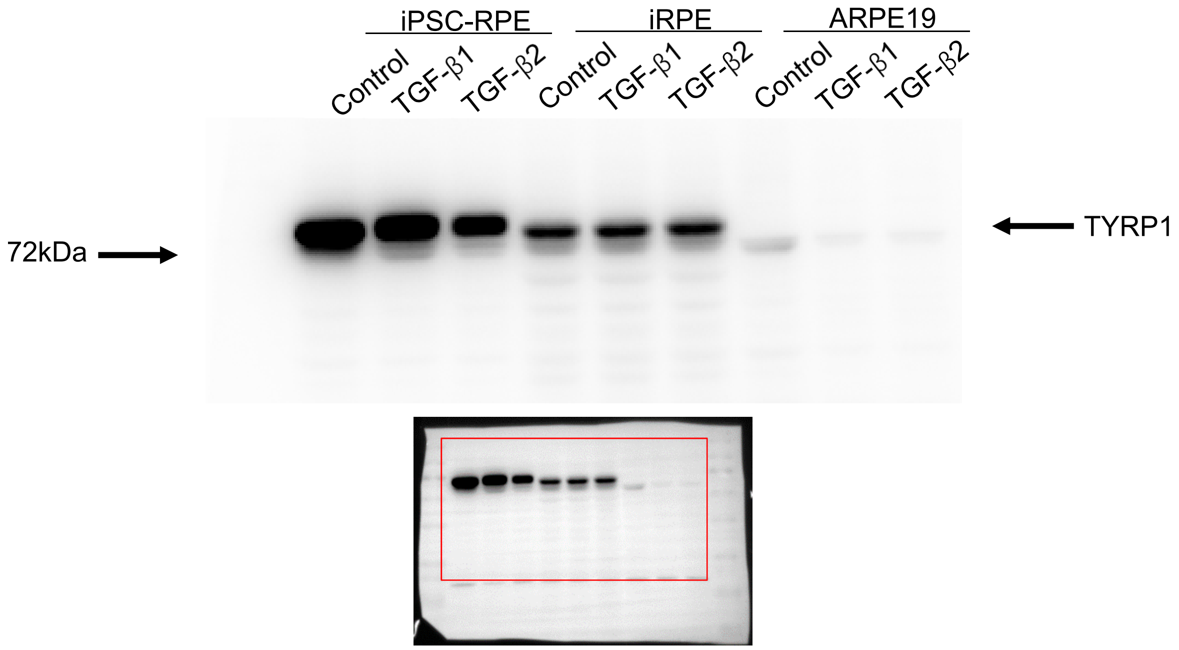

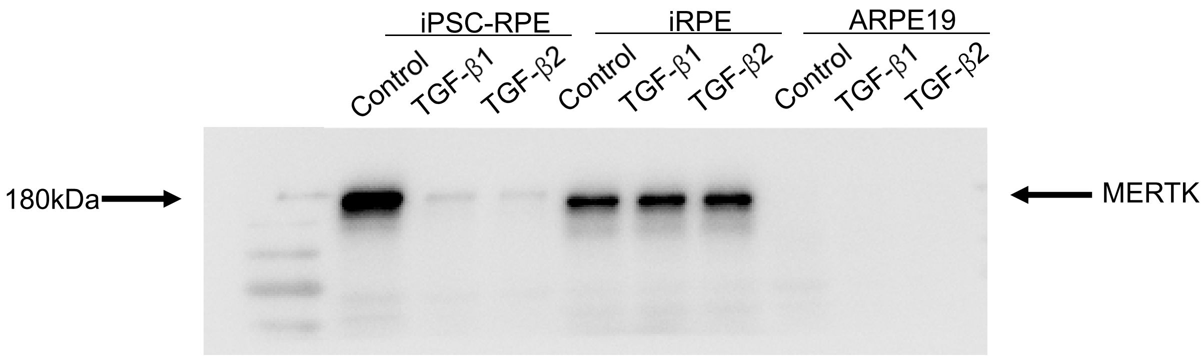
iPSC-RPE
iRPE
ARPE19
Control
TGF-β1
TGF-β2
Control
TGF-β1
TGF-β2
Control
TGF-β1
TGF-β2
180kDa
MERTK

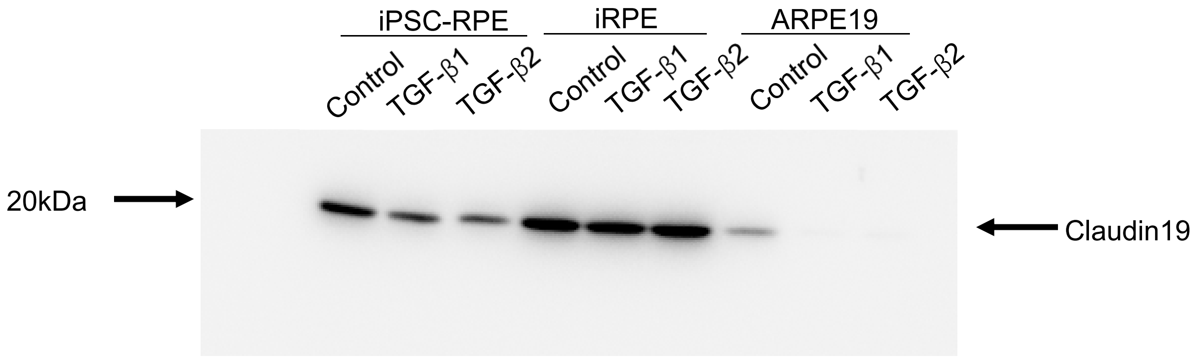
iPSC-RPE
iRPE
ARPE19
Control
TGF-β1
TGF-β2
Control
TGF-β1
TGF-β2
Control
TGF-β1
TGF-β2
20kDa
Claudin19

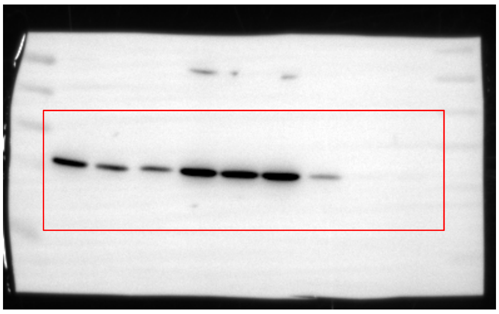

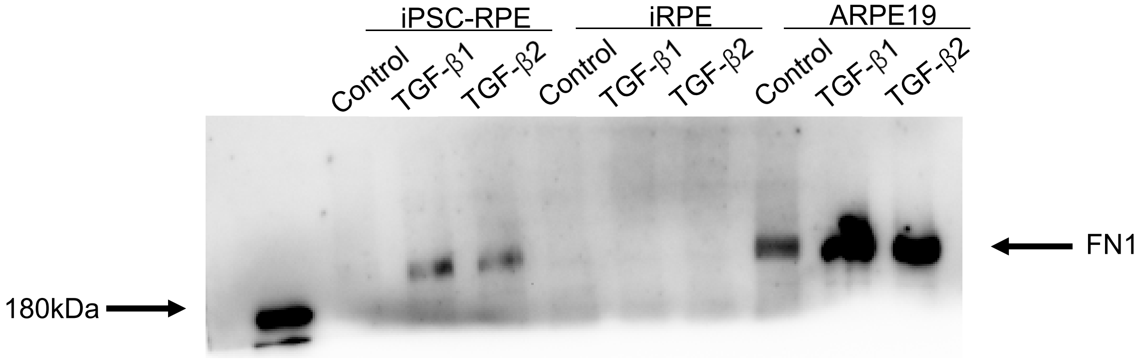
iPSC-RPE
iRPE
ARPE19
Control
TGF-β1
TGF-β2
Control
TGF-β1
TGF-β2
Control
TGF-β1
TGF-β2
FN1
180kDa

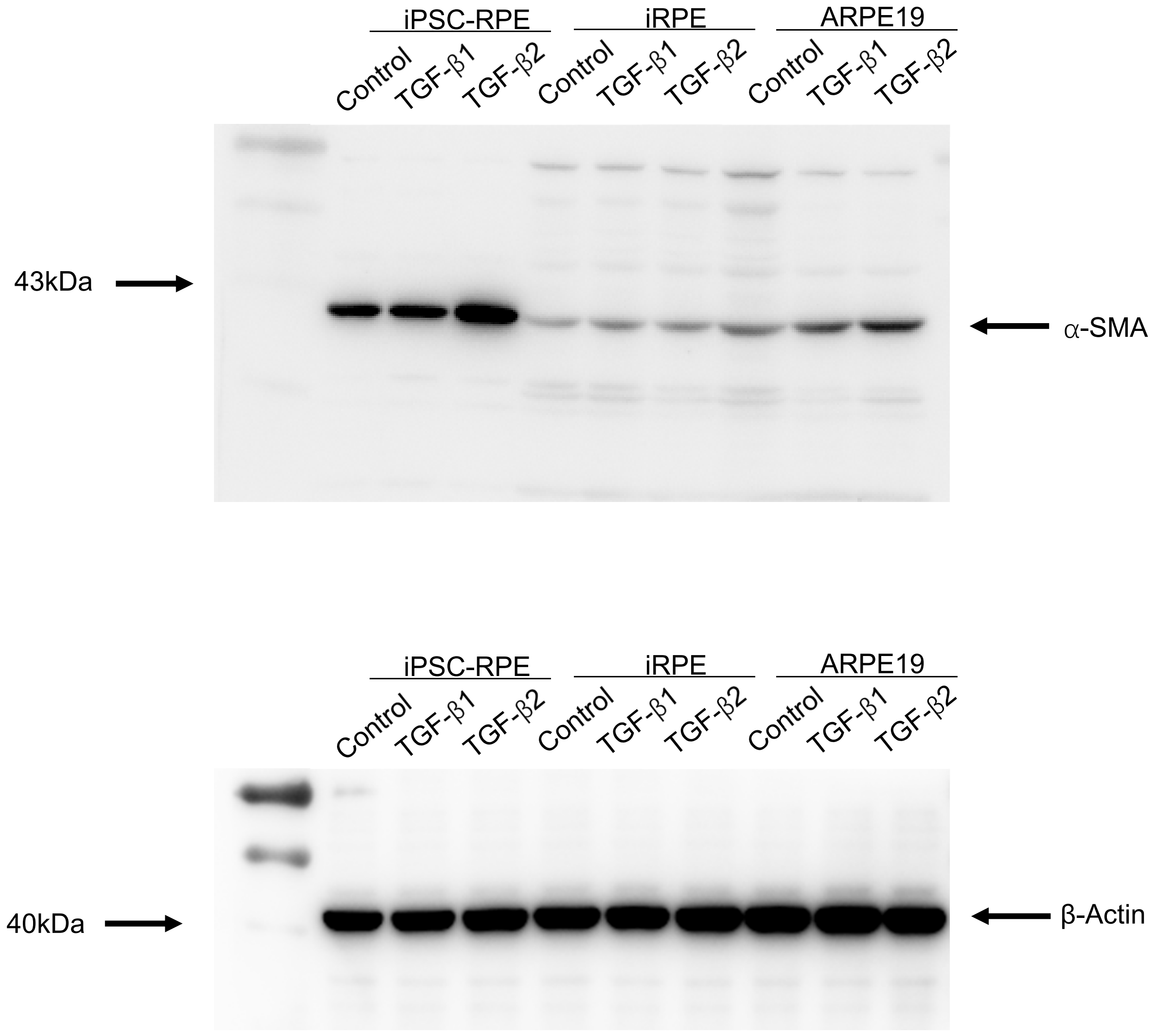

Supplementary figure 7A.

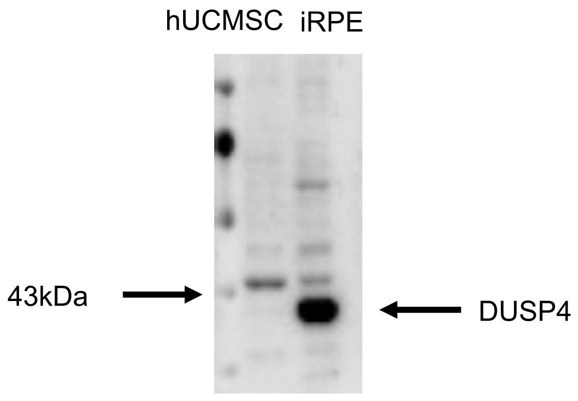

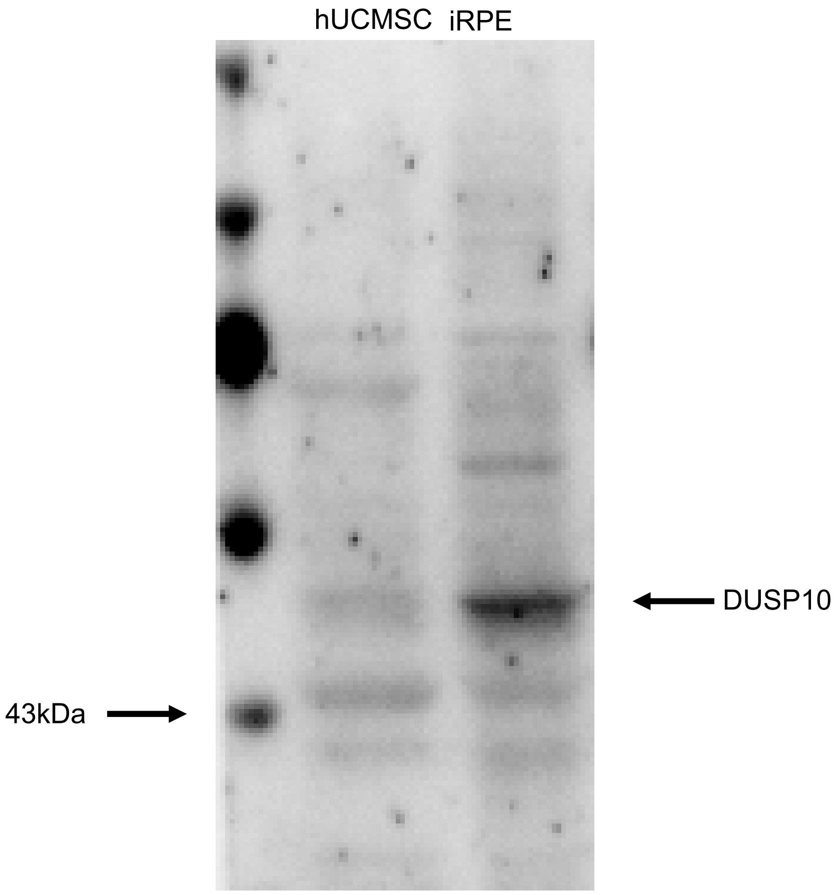
hUCMSC
iRPE
DUSP10
43kDa

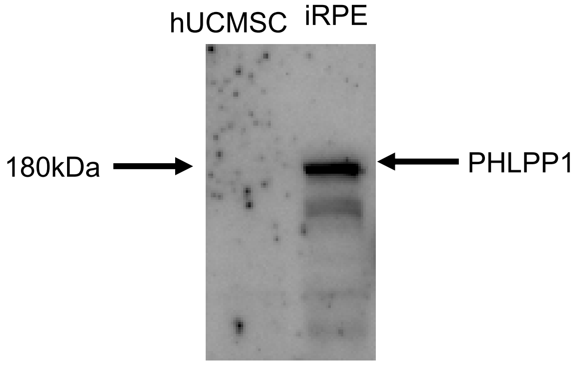
hUCMSC
iRPE
180kDa
PHLPP1

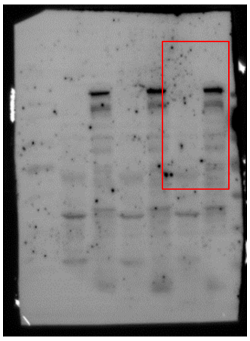

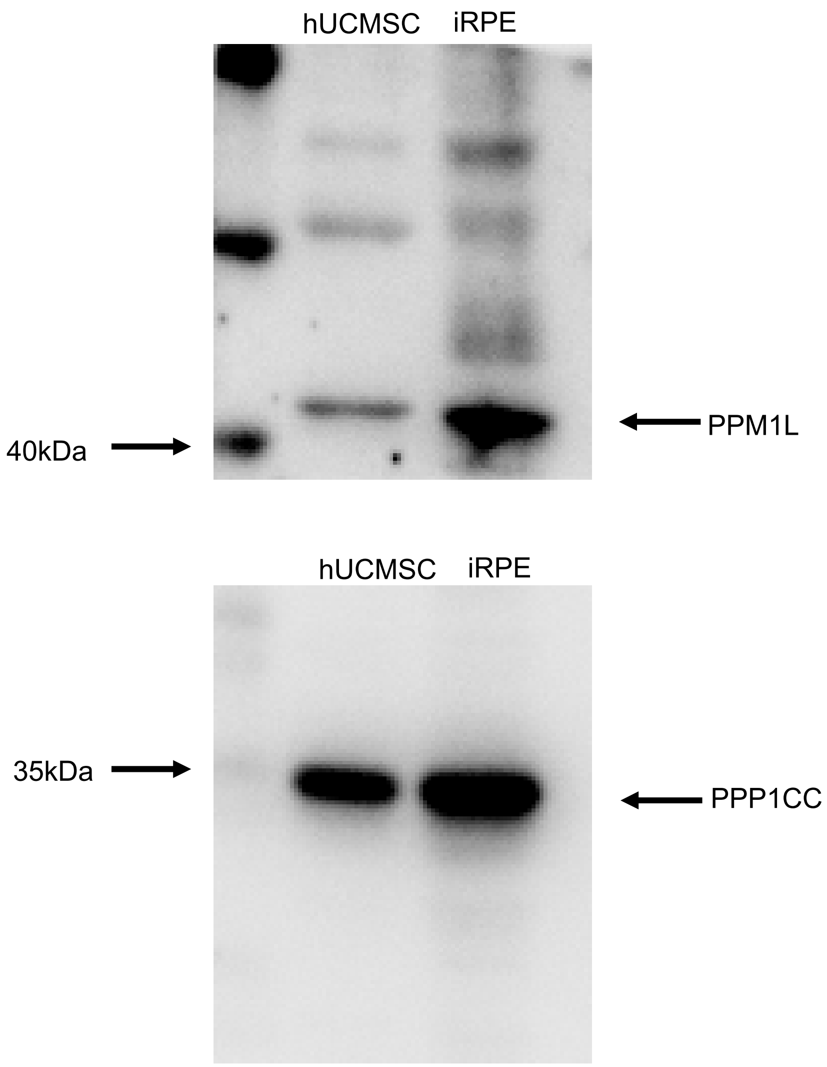
hUCMSC
iRPE
40kDa
PPM1L
hUCMSC
iRPE
35kDa
PPP1CC

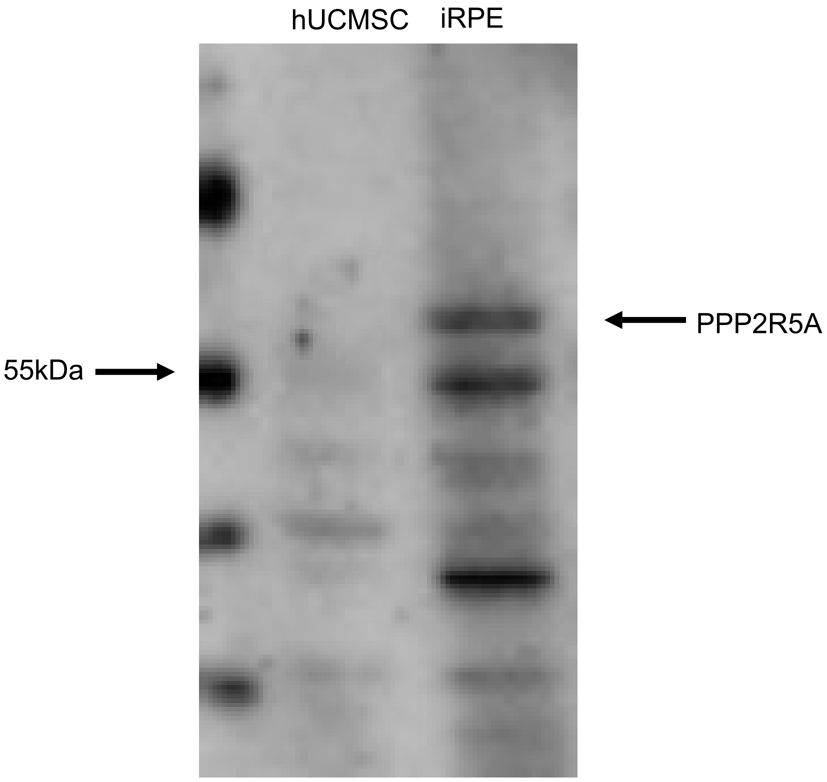
hUCMSC
iRPE
PPP2R5A
55kDa

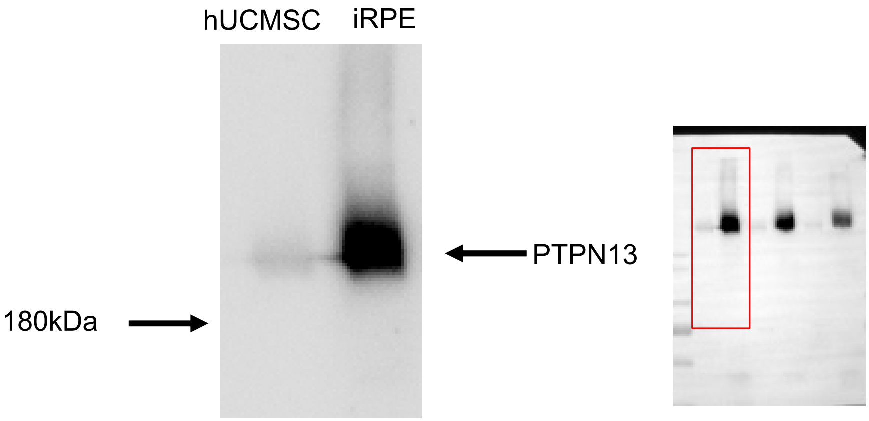
hUCMSC
iRPE
PTPN13
180kDa

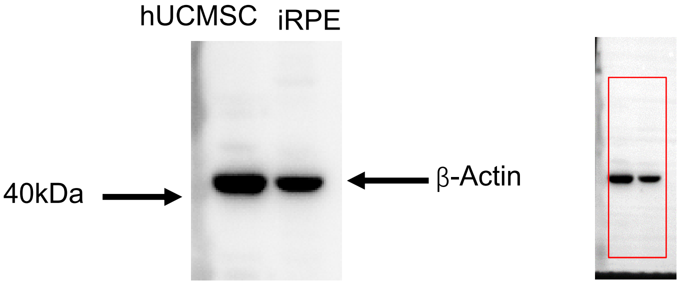
hUCMSC
iRPE
β-Actin
40kDa
